# Global, regional, and national burden of heatwave-related mortality from 1990 to 2019: A three-stage modelling study

**DOI:** 10.1371/journal.pmed.1004364

**Published:** 2024-05-14

**Authors:** Qi Zhao, Shanshan Li, Tingting Ye, Yao Wu, Antonio Gasparrini, Shilu Tong, Aleš Urban, Ana Maria Vicedo-Cabrera, Aurelio Tobias, Ben Armstrong, Dominic Royé, Eric Lavigne, Francesca de’Donato, Francesco Sera, Haidong Kan, Joel Schwartz, Mathilde Pascal, Niilo Ryti, Patrick Goodman, Paulo Hilario Nascimento Saldiva, Michelle L. Bell, Yuming Guo, on behalf of the MCC Collaborative Research Network

**Affiliations:** 1 Department of Epidemiology, School of Public Health/Qilu hospital, Cheeloo College of Medicine, Shandong University, Jinan, China; 2 Climate, Air Quality Research Unit, School of Public Health and Preventive Medicine, Monash University, Melbourne, Australia; 3 Environment & Health Modelling (EHM) Lab, Department of Public Health Environments and Society, London School of Hygiene & Tropical Medicine, London, United Kingdom; 4 National Institute of Environmental Health, Chinese Center for Disease Control and Prevention, Beijing, China; 5 School of Public Health and Social Work, Queensland University of Technology, Brisbane, Australia; 6 Institute of Atmospheric Physics, Academy of Sciences of the Czech Republic, Prague, Czech Republic; 7 Faculty of Environmental Sciences, Czech University of Life Sciences, Prague, Czech Republic; 8 Institute of Social and Preventive Medicine, University of Bern, Bern, Switzerland; 9 Oeschger Center for Climate Change Research, University of Bern, Bern, Switzerland; 10 Institute of Environmental Assessment and Water Research (IDAEA), Spanish Council for Scientific Research (CSIC), Barcelona, Spain; 11 Department of Public Health Environments and Society, London School of Hygiene & Tropical Medicine, London, United Kingdom; 12 Climate Research Foundation (FIC), Madrid, Spain; 13 Spanish Consortium for Research and Public Health (CIBERESP), Madrid, Spain; 14 School of Epidemiology & Public Health, Faculty of Medicine, University of Ottawa, Ottawa, Canada; 15 Environmental Health Science and Research Bureau, Health Canada, Ottawa, Canada; 16 Department of Epidemiology, Lazio Regional Health Service, Asl Roma 1, Rome, Italy; 17 Department of Statistics, Computer Science and Applications “G. Parenti”, University of Florence, Florence, Italy; 18 Department of Environmental Health, School of Public Health, Fudan University, Shanghai, China; 19 Department of Environmental Health, Harvard T.H. Chan School of Public Health, Boston, Massachusetts, United States of America; 20 Santé Publique France, Department of Environmental and Occupational Health, French National Public Health Agency, Saint Maurice, France; 21 Center for Environmental and Respiratory Health Research (CERH), University of Oulu, Oulu, Finland; 22 Medical Research Center Oulu (MRC Oulu), Oulu University Hospital and University of Oulu, Oulu, Finland; 23 Department of Public Health, University of Helsinki, Helsinki, Finland; 24 Technological University, Dublin, Ireland; 25 INSPER, São Paulo, Brazil; 26 School of the Environment, Yale University, New Haven, Connecticut, United States of America; 27 Korea University, Seoul, South Korea

## Abstract

**Background:**

The regional disparity of heatwave-related mortality over a long period has not been sufficiently assessed across the globe, impeding the localisation of adaptation planning and risk management towards climate change. We quantified the global mortality burden associated with heatwaves at a spatial resolution of 0.5°×0.5° and the temporal change from 1990 to 2019.

**Methods and findings:**

We collected data on daily deaths and temperature from 750 locations of 43 countries or regions, and 5 meta-predictors in 0.5°×0.5° resolution across the world. Heatwaves were defined as location-specific daily mean temperature ≥95th percentiles of year-round temperature range with duration ≥2 days. We first estimated the location-specific heatwave-mortality association. Secondly, a multivariate meta-regression was fitted between location-specific associations and 5 meta-predictors, which was in the third stage used with grid cell-specific meta-predictors to predict grid cell-specific association. Heatwave-related excess deaths were calculated for each grid and aggregated. During 1990 to 2019, 0.94% (95% CI: 0.68–1.19) of deaths [i.e., 153,078 cases (95% eCI: 109,950–194,227)] per warm season were estimated to be from heatwaves, accounting for 236 (95% eCI: 170–300) deaths per 10 million residents. The ratio between heatwave-related excess deaths and all premature deaths per warm season remained relatively unchanged over the 30 years, while the number of heatwave-related excess deaths per 10 million residents per warm season declined by 7.2% per decade in comparison to the 30-year average. Locations with the highest heatwave-related death ratio and rate were in Southern and Eastern Europe or areas had polar and alpine climates, and/or their residents had high incomes. The temporal change of heatwave-related mortality burden showed geographic disparities, such that locations with tropical climate or low incomes were observed with the greatest decline. The main limitation of this study was the lack of data from certain regions, e.g., Arabian Peninsula and South Asia.

**Conclusions:**

Heatwaves were associated with substantial mortality burden that varied spatiotemporally over the globe in the past 30 years. The findings indicate the potential benefit of governmental actions to enhance health sector adaptation and resilience, accounting for inequalities across communities.

## Abstract 2

Qi Zhao and colleagues modelled the global, regional and national burden of heatwave-related mortality from 1990 to 2019.

## Author summary

**Why was this study done?:**

- Although exposure to heatwave has been associated with increased risk of excess deaths and certain temporal attenuation observed, the evidence mainly comes from limited locations.
- The regional disparity in heatwave-related mortality burden over a long period has not been adequately quantified across the globe.

**What did the researchers do and find?:**

- Heatwave-related excess deaths were predicted to each grid cell (0.5°×0.5°) across the globe between 1990 and 2019 using a three-stage modelling framework.
- During 1990 to 2019 warm seasons, 153,078 deaths were associated with heatwaves (nearly half in Asia), which accounted for 0.94% of all deaths and equated 236 deaths per 10 million residents. The global heatwave-related excess death rate declined by 7.2% per decade in comparison to the 30-year average.
- Heatwave-related mortality showed complex regional disparities, such that Southern and Eastern Europe or areas had polar and alpine climates, and/or their residents had high incomes had the greatest cumulative burden while certain locations in Oceania or those with tropical climate or low incomes were observed with the greatest decline over decades.

**What do these findings mean?:**

- These regional disparities suggest the necessity of considering the cumulation and temporal change of mortality burden for evaluating the historical health impact of climate change and developing time-efficient adaptation strategies for a certain area.
- Localised adaptation planning and risk management should be encouraged across all levels of government, with considering subnational capacities and inequalities.
- Lack of time-series death data from certain regions, e.g., Arabian Peninsula and South Asia, may reduce the prediction accuracy in those regions.

## Introduction

Heatwaves are periods of extremely high ambient temperatures that last for a few days. In comparison to moderate weather events, heatwaves are particularly impactful for population health by imposing overwhelming thermal stress on human body and triggering dysfunction of multiple organs. Direct outcomes include heat exhaustion, heat cramps, and heatstroke [1]. The decompensated heat stress can also aggravate preexisting chronic conditions, leading to premature deaths, psychiatric disorders, and other outcomes [2]. As the result, the occurrence of a single heatwave is linked to a substantial disease burden and thus of important public health concerns across countries and regions.

In comparison to 1850 to 1990, the global surface temperature has increased by 1.14°C in 2013 to 2022 and is expected to increase by another 0.41 to 3.41°C by 2081 to 2100 [3]. Despite the worldwide overall warming trend, the frequency, strength, and duration of heatwaves have been changing in various spatial patterns within and across countries. Estimating heatwave-related mortality over an extended historical period is important for quantifying how long-term climate change has affected population health. However, previous long-term estimations mainly came from limited locations within single countries [4,5]. The lack of regional and temporal evaluation limits the comprehensive understanding of heatwave-related health burden and its change across the world.

The Multi-Country Multi-City (MCC) Collaborative Research Network was established in 2014 [6]. By 2021, the MCC had collected time-series data on mortality and weather conditions from 43 countries or regions. These countries or regions account for over 46% of the global population and 75% of gross domestic product (GDP). Using the MCC database, this study aims to assess the dynamic change in heatwave-related excess deaths at a spatial resolution of 0.5°×0.5° from 1990 to 2019. This study will provide extensive global scale evidence, which can be used to inform inter-governmental actions aiming at mitigating the impacts of extremely high temperature on human health.

## Methods

### Data sources

**Fig 1:**
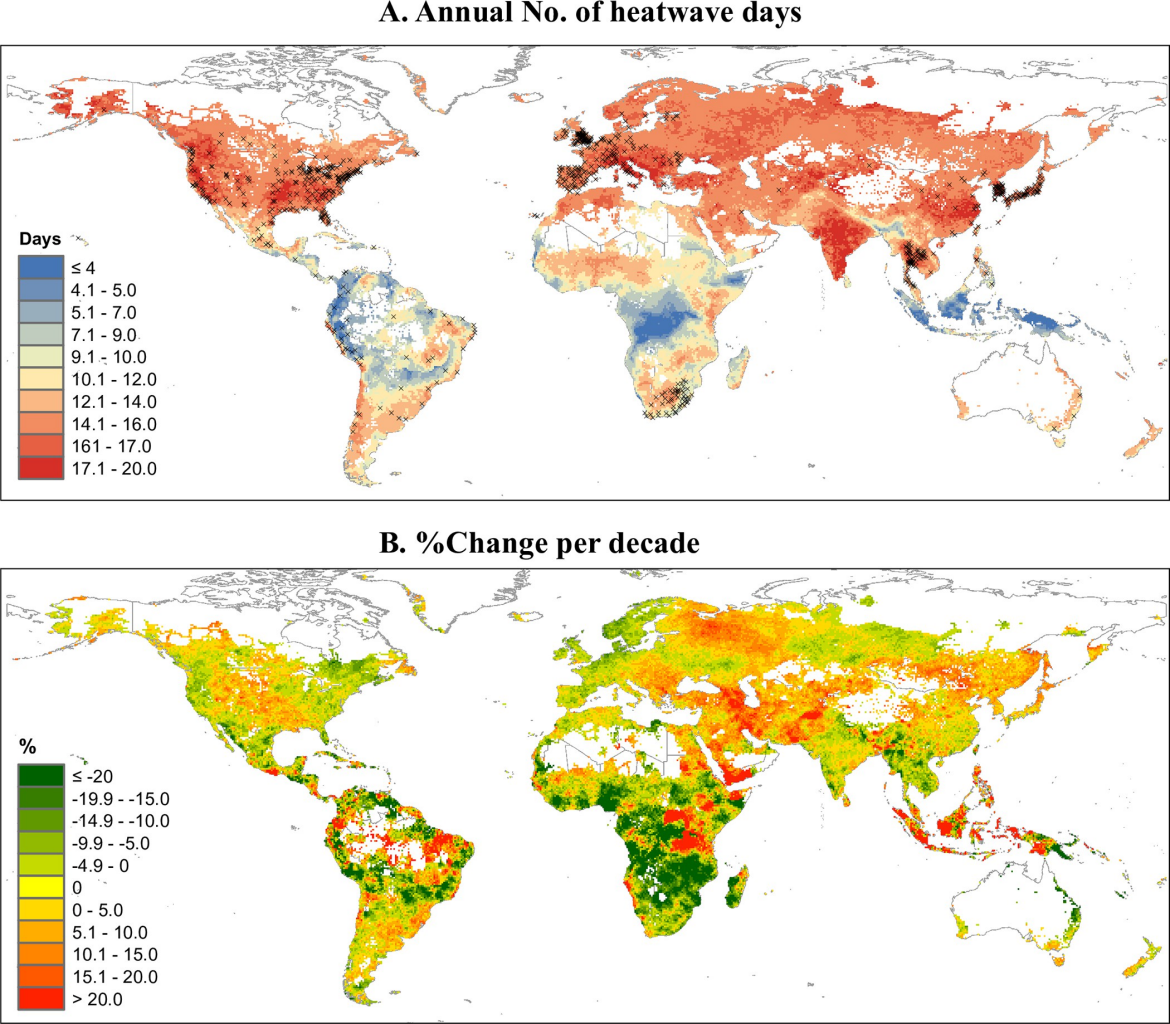
**Heatwave days (A) and the change per decade (B) at a spatial resolution of 0.5°×0.5° across the globe per warm season during 1990–2019.** The black crosses in Fig 1A represent the 750 locations from the 43 countries or regions used in the first-stage analysis. Only grid cells with at least 1 annual death were included. For each grid cell, heatwaves were defined as at least 2 consecutive days with mean temperatures above the 95th percentile of the year-round daily mean temperatures. $\%Change\;per\;decade=\frac{Change\;per\;decade}{The\;mean\;value\;in\;1990-2019}\times 100\%$. Change per decade is calculated using a linear regression. The base layer of the world map was imported from the public domain Natural Earth project (source: https://www.naturalearthdata.com/downloads/; terms of use: www.naturalearthdata.com/about/terms-of-use/). Summarised data at the regional level are provided in S5 Table.

In line with previous MCC research, this is a time-series modelling study. MCC data has been described in our previous publications [7,8], with details provided in S2 Text and S1 Table. Briefly, the MCC network collects daily time-series data on deaths and weather conditions at the city or community level from a range of countries/regions. This study used the dataset covering daily death counts for all causes or when such data unavailable, for non-external causes [International Classification of Diseases (ICD)-9: 0–799 or ICD-10: A00-R99] from 750 locations of 43 countries or regions (marked with black crosses in Fig 1A). The period of data collection overlapped largely, ranging from 1969 to 2018.

Data on daily minimum and maximum temperatures between 1990 and 2019 at a spatial resolution of 0.5°×0.5° were obtained from the Climate Prediction Center Global Unified Temperature Data (https://psl.noaa.gov), with the daily mean temperature calculated as the mean. Yearly GDP data (calibrated to the 2005 inflation rate) and population size in 1990, 2000, 2010, and 2020 were obtained from the Global Carbon Project at a spatial resolution of 0.5°×0.5° [9]. The annual data were interpolated linearly for each grid. Age-specific mortality rate and population size for each country were obtained for the period 1990 to 2019 from the Global Burden of Disease Study 2019 [10]. The standard population age structure was collected from the World Health Organization (WHO) [11]. A detailed description is provided in S3–S5 Texts on the interpolation of annual GDP and population size, the calculation of GDP per capita, and the calculation of grid-specific daily mean deaths (crude and age-standardised) in the warm season.

### Definition of heatwaves

Globally, there is no standard heatwave definition either in scientific research or in policy, but are defined based on temperature intensity and duration. In line with our previous publications [4,12], heatwaves were defined for each location as daily mean temperature ≥95th percentiles of year-round temperature range with duration ≥2 days (see details in S6 Text). All analyses were limited to the warm season—the hottest 4 consecutive months.

### Statistical analysis

Following previous MCC studies, a three-stage meta-analytical strategy was used to estimate the excess deaths associated with heatwaves [13,14]. The strength of heatwave-mortality association may vary temporally due to long-term acclimatisation [15]. We considered it using time-varied effect estimates of heatwaves and heatwave definitions (see details in S7 and S8 Texts). In addition, the heatwave-related excess deaths may be also influenced by population factors, which were considered using time-varied mortality and population. First, we quantified the heatwave-mortality association separately in the 750 locations. Then, a meta-regression model was built between the location-specific association and predictors. Finally, this regression with grid cell-specific predictors was used to predict the association for each grid cell per decade, and then calculate the excess deaths without and with adjusting mortality rate by the age structure of WHO standard population. This standardisation allowed to compare the impact of heatwave across regions after controlling for population ageing (see explanation in S7 Text).

In the first stage, for each of the 750 locations, a quasi-Poisson regression with constrained distributed lag model was used to estimate the heatwave-mortality association. Long-term trend was controlled using a natural cubic spline with 1 degree of freedom (df) per 10 years. Seasonality was controlled using a natural cubic spline with 4 df for day of the season. The assumption of a constant seasonal trend was relaxed using an interaction between this natural cubic spline and an indicator of year. Day of the week was controlled using a categorical variable. The distributed lag effects of heatwaves (0–1 variable; where 0 describes “non-heatwave days” and 1 describes “heatwave days”) on mortality were captured using a natural cubic spline with 4 df for up to 10 days, with 2 internal knots placed equally spaced values in the log scale of lag days [4,12].

In the second stage, a meta-regression was built between the reduced overall association and a set of predictors for each location. Following our previous studies [8,13], 5 predictors were collected that could potentially explain certain heterogeneity in the heatwave-mortality association across the 750 locations: continent, Köppen–Geiger climate classification, GDP per capita, and the average and the range of daily mean temperature in the warm season (see details of predictor selection in S7 Text and S2 and S3 Tables).

In the third stage, we predicted the grid cell-specific association using the meta-regression in the second stage and the 5 meta-predictors by each decade. For grid cell *i*, the expected excess deaths in the warm season of year *t* in a certain decade was calculated as follows:

$${ED}_{it}={N.D}_{it}\times ({RR}_{it}-1)\times {N.HW}_{it}$$

$${N.D}_{it}={N.POP}_{it}\times {MR}_{it}\times {S.R}_{it}$$

where *ED_it_* is the excess deaths explainable by heatwaves; *N*.*D_it_* is the daily average number of deaths during the warm season for the decade; *RR_it_* is the relative risk of mortality associated with heatwave; *N*.*HW_it_* is the heatwave days in the warm season of year t, which is defined using the year-round distribution of temperature in a certain decade; *N*.*POP_it_* is the annual population size; *MR_it_* is the mean daily mortality rate (crude and standardised by WHO population structure) calculated by averaging the annual mortality rate (see details in S4 and S5 Texts); *S*. *R_it_* is the ratio between hot season and year-round deaths. Excess deaths for each grid cell were then aggregated at the national, regional, and global levels.

In addition, we also quantified the ratio (%) between heatwave-related excess deaths and total deaths in the warm season (i.e., excess death ratio), and the heatwave-related excess deaths per 10 million residents (i.e., excess death rate). The empirical confidence intervals (eCIs) were calculated using Monte Carlo simulations (500 samples) to quantify the uncertainty in estimating the excess mortality burden by assuming a normal distribution for the coefficient of heatwave effect estimate. Analyses were restricted to grid cells with at least 1 annual death, which in total accounted for 99.995% of the global population.

The robustness of findings was tested via sensitivity analyses. Differences in the effect sizes of heatwaves fitted using all-cause mortality and non-external cause mortality were compared for locations with both data available. The variation in the heatwave-mortality association was examined by changing the maximum lag days of heatwave to 15 days, the df of lag days from 3 to 5, the df for seasonality from 3 to 7, and by additionally adjusting for relative humidity. Meta-regression was used to examine the significance of inter-group difference.

Analyses were conducted in R language (version 4.0.2). “dlnm” and “mixmeta” packages were used to perform the quasi-Poisson regression with constrained distributed lag models and multivariate meta-regressions, respectively [16,17].

## Results

**Fig 2:**
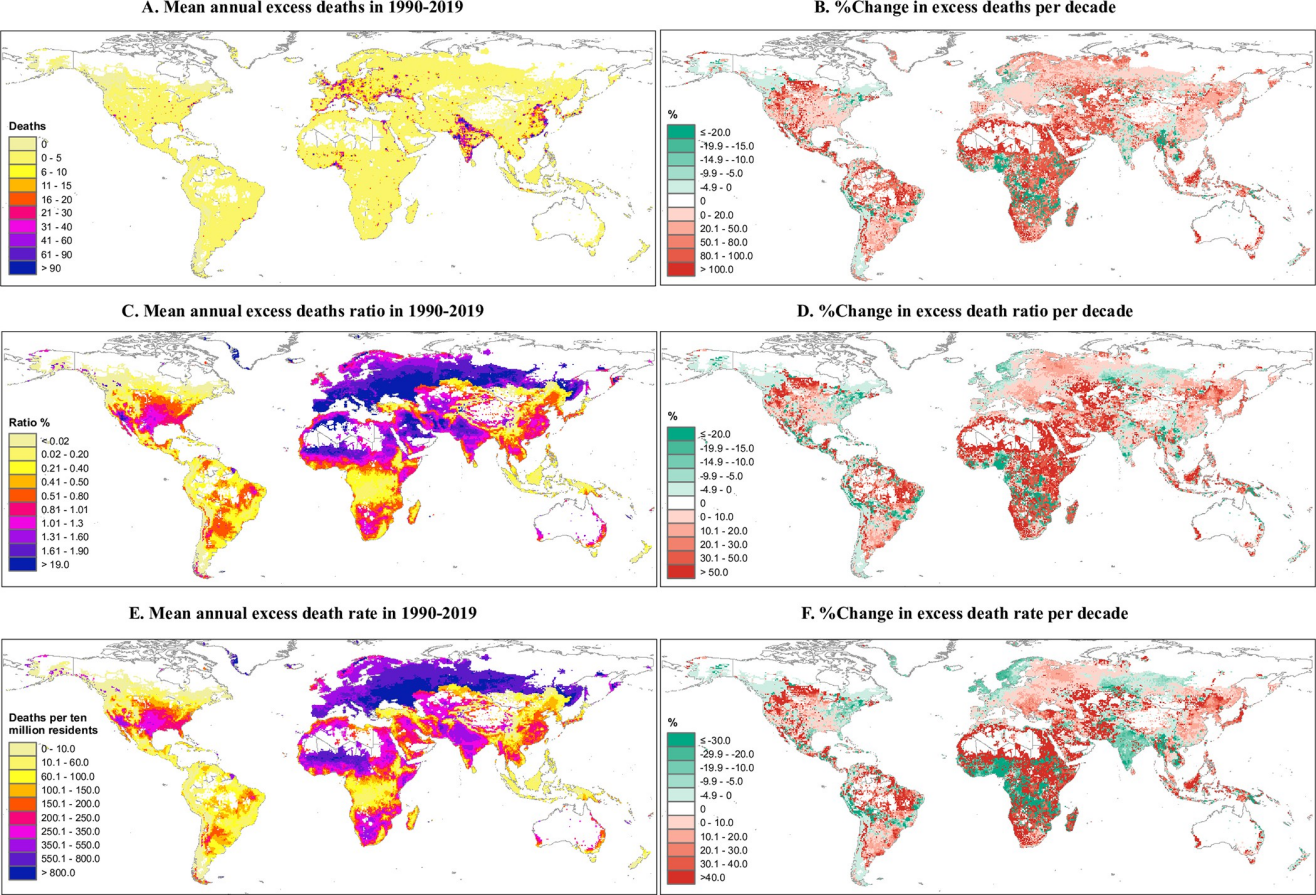
**Average and the change per decade of excess deaths (A, B), death ratio (%, C, D), and deaths per 10 million residents (E, F) associated with heatwaves in 1990–2019 at a spatial resolution of 0.5°×0.5°.** Only grid cells with at least 1 annual death were included. $\%Change\;per\;decade=\frac{Change\;per\;decade}{The\;mean\;value\;in\;1990-2019}\times 100\%$. Change per decade is calculated using a linear regression. The base layer of the world map was imported from the public domain Natural Earth project (source: https://www.naturalearthdata.com/downloads/; terms of use: www.naturalearthdata.com/about/terms-of-use/). Summarised data at the country level are provided in S6, S8, and S10 Tables.

From 1990–1999 to 2010–2019, annual heatwaves increased from 13.4 to 13.7 days across all grid cells, with the ambient mean temperature warming by 0.35°C per decade (Fig 1 and S4 and S5 Tables). Globally, average heatwave-related excess deaths were 153,078 (95% eCI: 109,950–194,227) per warm season in the scenario without considering mortality standardisation. Of these, 48.95% of deaths occurred in Asia, 31.56% in Europe, 13.82% in Africa, 5.37% in America, and 0.28% in Oceania (Table 1). Clusters of high excess deaths were observed in Eastern and Southern Asia, Eastern and Southern Europe, and areas close to Gulf of Guinea in Africa (see more details in Fig 2A). India (20.74%), China (13.82%), and Russia (7.89%) were the leading countries experiencing heatwave-related excess deaths (S6 Table). These regional differences still existed after adjusting country-specific mortality by WHO standard population (S7 Table and S1 Fig).

**Table 1:** Average excess deaths, death ratio, and deaths per 10 million residents associated with heatwaves per warm season during 1990–2019 by continent and region.

	Excess deaths (eCIs)		Excess death ratio% (eCIs)	Excess deaths per 10 million residents (eCIs)
	Cases	Proportion%		
**Global**	153,078 (109,950 to 194,227)	100	0.94 (0.68 to 1.19)	236 (170 to 300)
**Americas**	8,227 (4,093 to 12,240)	5.37	0.44 (0.22 to 0.65)	94 (47 to 139)
North America	4,823 (2,785 to 6,827)	3.15	0.58 (0.33 to 0.82)	147 (85 to 208)
Latin American and Caribbean	3,405 (1,311 to 5,452)	2.22	0.33 (0.13 to 0.52)	62 (24 to 99)
**Europe**	48,318 (41,907 to 54,526)	31.56	1.96 (1.70 to 2.21)	655 (568 to 739)
Northern Europe	3,960 (3,278 to 4,567)	2.59	1.35 (1.12 to 1.56)	407 (337 to 469)
Southern Europe	10,170 (8,945 to 11,269)	6.64	2.32 (2.04 to 2.57)	668 (588 to 741)
Western Europe	9,478 (8,250 to 10,599)	6.19	1.75 (1.52 to 1.95)	507 (441 to 567)
Eastern Europe	24,709 (21,153 to 28,101)	16.14	2.08 (1.78 to 2.36)	820 (702 to 933)
**Africa**	21,160 (12,125 to 29,557)	13.82	0.69 (0.39 to 0.96)	229 (131 to 320)
Northern Africa	4,539 (3,156 to 5,798)	2.97	1.20 (0.83 to 1.53)	239 (166 to 305)
Sub-Saharan Africa	16,622 (8,969 to 23,782)	10.86	0.62 (0.33 to 0.88)	227 (122 to 324)
**Asia**	74,939 (51,261 to 97,419)	48.95	0.85 (0.58 to 1.11)	192 (131 to 249)
Central Asia	1,260 (810 to 1,675)	0.82	0.94 (0.60 to 1.24)	217 (140 to 289)
Southern Asia	40,731 (28,869 to 52,207)	26.61	1.06 (0.75 to 1.36)	257 (182 to 330)
Western Asia	3,161 (2,151 to 4,064)	2.06	0.90 (0.61 to 1.16)	151 (103 to 194)
Eastern Asia	24,080 (15,892 to 31,270)	15.73	0.75 (0.49 to 0.97)	161 (106 to 209)
South-eastern Asia	5,708 (3,141 to 8,092)	3.73	0.46 (0.25 to 0.65)	101 (55 to 143)
**Oceania**	433 (−68 to 933)	0.28	0.61 (−0.10 to 1.32)	133 (−21 to 286)
Australia and New Zealand	342 (−62 to 749)	0.22	0.67 (−0.12 to 1.46)	137 (−25 to 300)
Other regions in Oceania*	91 (8 to 182)	0.06	0.46 (0.04 to 0.93)	118 (10 to 237)

*Other regions in Oceania are defined as all areas outside of Australia and New Zealand in Oceania. All other regions in the table are defined according to the UN Statistics Division (M49) regional groupings.

eCIs, empirical CIs.

Heatwave-related excess deaths accounted for 0.94% (95% eCI: 0.68–1.19) of global deaths during the warm seasons of 1990 to 2019, equating to 236 (95% eCI: 170–300) deaths per 10 million residents. When the spatial distribution of the heatwave-related excess death ratio and rate were examined for the 5 continents, Europe had the highest ratio (1.96%) and rate (655 deaths per 10 million residents), with a substantial burden observed in southern and eastern Europe (Table 1 and Fig 2C and 2E). The area between Northern Africa, Arabian Peninsula, and Southern Asia was another region with high vulnerability. In contrast, the excess death ratio and rate in South America were one-third (or less) of the global average. Similar geographic patterns aforementioned were also observed after mortality standardisation (S1 Fig). At the national level, Greece, Malta, and Italy had the highest excess death ratios during the 30-year period for both mortality calculation scenarios (2.47% to 2.59%, S8 and S9 Tables). When the excess death rate was compared at the national level, the highest rates before mortality standardisation were observed in Ukraine, Bulgaria, and Hungary (S10 Table). Following mortality standardisation, the highest rates were observed in Niger, Chad, and Ukraine (S11 Table). Of note, in general locations with the highest unstandardised heatwave-related excess death ratio and rate had polar and alpine climates, and/or their residents had high incomes. In contrast, after standardisation, the largest burdens were observed in locations with dry climates and/or their residents had lower-middle incomes (S12–S17 Tables).

**Fig 3:**
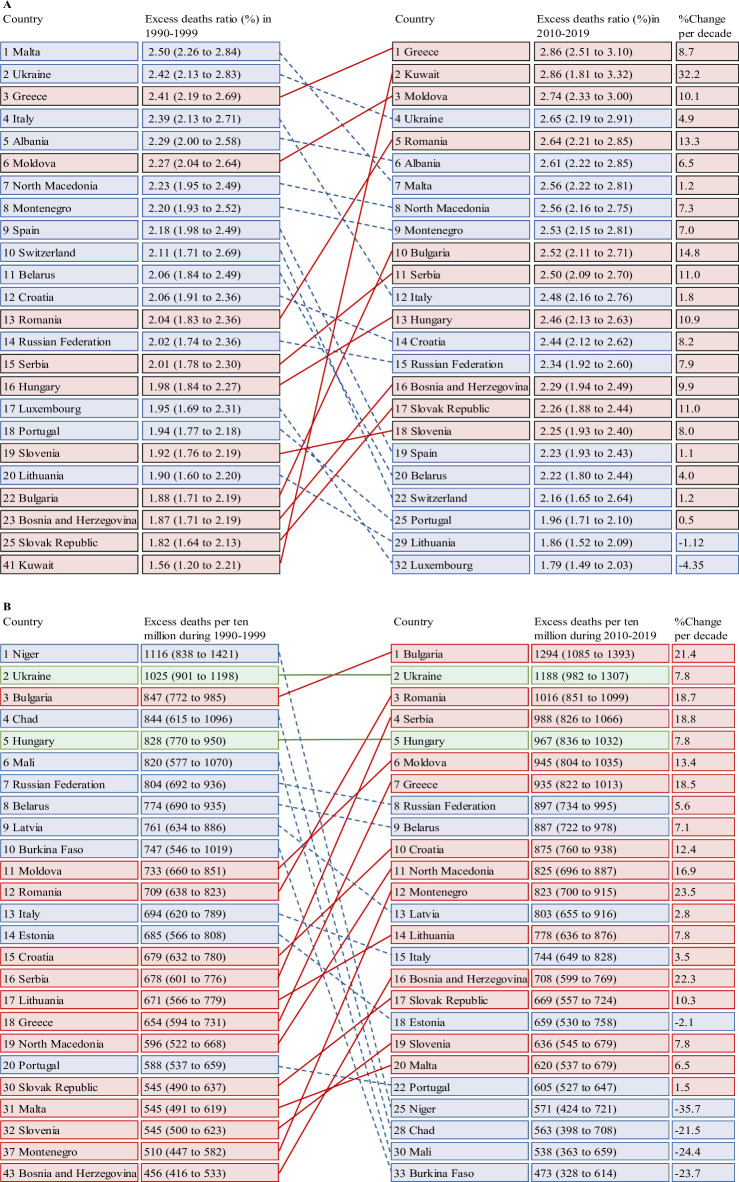
Leading 20 countries for excess death ratio (%) and deaths per 10 million residents associated with heatwaves per warm season from 1990–1999 to 2010–2019. $\%Change\;per\;decade=\frac{Change\;per\;decade}{The\;mean\;value\;in\;1990-2019}\times 100\%$. Change per decade is calculated using a linear regression. Some countries might drop out or new countries may appear from the first to the last decade due to the changing burden. The underlying data are provided in S8 and S10 Tables.

From 1990–1999 to 2010–2019, the global heatwave-related excess death ratio remained relatively constant, while the excess death rate (unstandardised) declined by 7.2% per decade in comparison to the 30-year average (Table 2). Regionally, the greatest growth occurred in Western Asia and Eastern Europe, although the burden in certain sites declined (Fig 2D and 2F). The greatest decline in heatwave-related mortality burdens were observed in other regions in Oceania, the central-west of sub-Saharan Africa, and certain locations of Southern Asia. After mortality standardisation, the observed spatial distribution changed. More regions were observed with declining heatwave-related mortality burdens, resulting in a 13.9% global reduction per decade, in comparison to the 30-year average (S1 Fig and S11 Table). As shown in Figs 3 and S2–S4, when the 20 countries with the highest heatwave-related mortality burden were listed, for the period 1990 to 1999 and the period 2010 to 2019, the order in which the countries were ranked varied considerable across time. Locations or countries with tropical climates and/or low incomes had the greatest decline in their heatwave-related excess death rates, while the excess death ratio varied slightly (S14–S17 Tables). Country-specific relative risk of heatwave-related mortality per decade pooled from grid cells was provided in S18 Table.

**Table 2:** Average excess death ratio and deaths per 10 million residents associated with heatwaves per warm season from 1990–1999 to 2010–2019 by continent and region.

	Excess death ratio (%)			Excess deaths per 10 million residents		
	1990–1999 (eCIs)	2010–2019 (eCIs)	Change per decade%	1990–1999 (eCIs)	2010–2019 (eCIs)	Change per decade%
**Global**	0.96 (0.70 to 1.24)	0.97 (0.68 to 1.21)	0.53	261 (191 to 337)	227 (159 to 283)	−7.2
**Americas**	0.46 (0.23 to 0.69)	0.45 (0.22 to 0.66)	−1.14	100 (50 to 150)	97 (48 to 143)	−1.6
Northern America	0.61 (0.35 to 0.86)	0.60 (0.35 to 0.84)	−0.86	158 (90 to 222)	153 (88 to 214)	−1.7
Latin American and Caribbean	0.34 (0.13 to 0.55)	0.33 (0.13 to 0.53)	−1.52	65 (24 to 106)	64 (25 to 102)	−0.81
**Europe**	1.94 (1.72 to 2.24)	2.10 (1.78 to 2.31)	4.08	648 (576 to 750)	687 (581 to 756)	2.98
Northern Europe	1.39 (1.16 to 1.62)	1.34 (1.10 to 1.53)	−1.85	456 (382 to 532)	373 (307 to 428)	−10.20
Southern Europe	2.23 (2.01 to 2.52)	2.40 (2.08 to 2.62)	3.66	628 (565 to 711)	713 (618 to 778)	6.36
Western Europe	1.79 (1.60 to 2.06)	1.71 (1.46 to 1.88)	−2.29	534 (478 to 613)	500 (427 to 549)	−3.35
Eastern Europe	2.04 (1.80 to 2.39)	2.36 (1.96 to 2.60)	7.69	783 (689 to 914)	904 (748 to 995)	7.38
**Africa**	0.76 (0.43 to 1.08)	0.71 (0.41 to 0.97)	−3.62	314 (181 to 450)	185 (107 to 252)	−28.17
Northern Africa	1.16 (0.83 to 1.52)	1.27 (0.87 to 1.60)	4.58	268 (192 to 352)	230 (158 to 289)	−7.95
Sub-Saharan Africa	0.70 (0.38 to 1.02)	0.62 (0.34 to 0.87)	−6.45	327 (178 to 478)	173 (94 to 243)	−33.92
**Asia**	0.85 (0.59 to 1.12)	0.89 (0.59 to 1.13)	2.35	205 (143 to 270)	190 (126 to 242)	−3.91
Central Asia	0.84 (0.56 to 1.17)	1.07 (0.66 to 1.36)	12.23	209 (139 to 289)	222 (137 to 283)	3.00
Southern Asia	1.07 (0.77 to 1.38)	1.08 (0.76 to 1.38)	0.47	308 (219 to 394)	230 (163 to 294)	−15.18
Western Asia	0.75 (0.53 to 1.01)	1.04 (0.69 to 1.31)	16.11	142 (100 to 191)	167 (111 to 210)	8.28
Eastern Asia	0.71 (0.49 to 0.96)	0.79 (0.50 to 0.99)	5.33	148 (102 to 200)	178 (112 to 222)	9.32
South-eastern Asia	0.50 (0.28 to 0.70)	0.50 (0.26 to 0.69)	0.00	114 (65 to 160)	106 (55 to 147)	−3.96
**Oceania**	0.68 (−0.09 to 1.49)	0.59 (−0.10 to 1.27)	−7.38	150 (−21 to 331)	126 (−22 to 272)	−9.02
Australia and New Zealand	0.67 (−0.13 to 1.54)	0.67 (−0.13 to 1.42)	0.00	142 (−27 to 328)	137 (−26 to 288)	−1.82
Other regions in Oceania*	0.71 (0.10 to 1.31)	0.38 (0.03 to 0.88)	−35.87	181 (26 to 336)	95 (7 to 219)	−36.44

*Other regions in Oceania are defined as all areas outside of Australia and New Zealand in Oceania. All other regions in the table are defined according to the UN Statistics Division (M49) regional groupings. $\%Change\;per\;decade=\frac{Change\;per\;decade}{The\;mean\;value\;in\;1990-2019}\times 100\%$. Change per decade is calculated using a linear regression.

eCIs, empirical CIs.

There was no significant difference in the fitted association using all-cause mortality or non-external cause mortality, changing length of maximum lag days, df of lag days, and df of seasonality, and additionally adjusting for relative humidity (S19 Table).

## Discussion

This study quantified the excess mortality burden associated with heatwaves at a spatial resolution of 0.5°×0.5°, and measured the temporal change from 1990 to 2019. In comparison to our previous global estimation, the excess deaths associated with heatwaves may explain approximately 30% of excess deaths associated with all temperatures above the optimum temperature threshold (i.e., including moderate heat and extreme heat) per year [13]. This suggests the cost-effectiveness of developing specific protective strategies against heatwaves considering the limited heatwave days (average 13.7 days per year in 2010 to 2019). Under the heatwave definition of this study, the heatwave-related excess death ratio remained relatively unchanged at the global level, while the death rate declined persistently. The cumulation and temporal change of heatwave-related mortality burden showed complex climatic and economic disparities across the world, especially after adjusting mortality by the age structure of WHO standard population.

Previously, several studies have explored the adverse impact of heatwaves on population health. For example, the risk of mortality in 272 Chinese cities increased by 7% during the 2013 to 2015 heatwave days, compared to non-heatwave days [5]. In Australia, mortality increased by 2% on heatwave days in 2007 to 2017 [18]. Our previous MCC study observed that the relative risk of mortality related to heatwave ranged from insignificant to 2.2 across the 400 locations of 18 countries or regions [4]. The Lancet Countdown 2022 Report estimated that adults older than 65 years in 2012 to 2021 experienced 3.2 more heatwave days per person than in 1986 to 2005 [19]. The substantial exposure risk to heatwave highlights the necessity of our study by providing the global overview (0.5°×0.5°) of heatwave-related mortality burden and the temporal change over decades.

In this study, we observed major clusters of heatwave-related excess deaths over the 30 years in Eastern and Southern Asia, Eastern and Southern Europe, and areas close to Gulf of Guinea in Africa. These clusters still existed after removing population ageing factor with the use of standardised mortality across the world, suggesting the high impact of heatwave exposure to local residents. In the perspective of planetary health, this finding indicates the arduous task for the international communities to cooperate with local governments in developing specific adaptation strategies to reduce the global heatwave vulnerability. From 1990 to 2019, the global population increased by over 2.3 billion, with the annual mortality rate declining from 0.87% to 0.73% [10]. Taking into account the time-varying population size and mortality, our study assessed the influence of heatwaves using the ratio between excess deaths and all deaths, and the excess deaths per 10 million residents. The 2 indicators were also the greatest in Europe (especially in the Southern and Eastern Europe), marking it the most affected region by heatwaves worldwide. In addition, the excess death ratio and rate were also substantial in the broad area between Northern Africa, Arabian Peninsula, and Southern Asia, which should not be overlooked.

Evidence shows that the global warming trend is accelerating, which has resulted in 19 of the 20 hottest years since 1880 occurring after 2000 [20]. In line with climate change, the Lancet Countdown 2022 Report estimated that the heat-related mortality for the elderly over 65 years increased by 68% from 2000–2004 to 2017–2021 [19]. Our study explored the temporal change of heatwave-related mortality burden over a longer period and by considering long-term acclimatisation into the analyses. Taking the temporal variation of annual mortality rate and population size into account, the global heatwave-related excess death ratio was unchanged, and the deaths per 10 million residents (unstandardised) declined by 7.2% per decade. Several studies considering heat adaptation also observed attenuation in effect of heatwaves over time [21,22]. From 1990 to 2019, the mean age of population at the global level increased by 18.8%, with the proportion of elderly aged over 70 years doubled. Parallelling with the ageing trend over time, we observed the decline pace of heatwave-related excess death rate doubled in the scenario of mortality standardisation. It is speculated that population ageing may explain certain proportion of the temporal change of heatwave-related deaths. These findings suggest the importance of considering heat adaptation, varied population structure, and other demographic factors during the estimation of disease burden associated with extreme temperature events over a long study period.

This study revealed the complex regional disparity in the cumulation and temporal change of heatwave-related mortality burden by climate and economic level. Over past 30 years, cumulative heatwave-related excess death ratio and rate were the highest in locations with polar and alpine climates and the lowest in tropical areas. In addition, locations with tropical climate also experienced the largest decline in heatwave-related mortality burden. This regional pattern still existed after adjusting mortality by the age structure of WHO standard population. Numerous research has reported that the same extreme high temperature may be less harmful in hot regions than in cold regions, and explained this phenomenon as due to long-term acclimatisation to local heat [6,7]. However, this theory may not fully explain the largest increment of heatwave-related mortality burden in areas with continental climate. The substantial difference in temporal change of heatwave-related excess death ratio between unstandardised and standardised mortality scenarios suggests population ageing may be a contributor. Nevertheless, more research is still necessary to illustrate the varied changing patterns across climate zones over decades.

Stratified results by income groups were more complicated. For example, low-income countries had the largest decline in heatwave-related excess death rate over the 30 years while the death ratio increased slightly. The global maps provided more information on geographic disparity. Although some regions (e.g., Southern Asia) had substantial cumulative excess deaths in the 30 years, the local heatwave-related burden was declining at a considerable pace. By contrast, certain regions were least affected cumulatively but with repaid increase burden over time. These regional disparities suggest the importance of considering the mean mortality burden and its temporal change for calculating the historical impact of climate change and developing time-efficient adaptation strategies for a certain area.

Findings of this study suggest that no area of the world is immune from the heatwave-mortality impact in the context of climate change, even as specific locations are experiencing more heat acclimatisation that other places. The socioeconomic costs of heatwaves are very likely to rise in the future [8]. However, the latest survey from the WHO shows that only half of 101 countries have developed national plans on climate change and health, with 4 countries having sufficient national funds to implement plans [23]. Even worse, the COVID-19 pandemic since early 2020 has disclosed the fragility of the health systems of numerous nations in responding to large emergency demands [24]. With a growing understanding of climate change’s threat and the inadequate preparation, intergovernmental actions should prioritise building adaptation and resilience, with consideration of national/subnational inequalities and the distribution of vulnerable populations.

This study has several key strengths. First, to the best of our knowledge, this is the largest study to quantify the mortality burden associated with heatwaves. In comparison to previous estimations at the national or subnational levels, our gridded maps improve the understanding of heatwave impact at the global scale and its regional disparity. Our findings may thus inform localised adaptation planning and risk management to address the warming climate across all levels of government. Another strength of our study is to measure the temporal change of heatwave-related mortality burden over a 30-year period. The findings help quantify how global warming has influenced population health across the world and benefit the projection of future burden under climate change scenarios. Finally, our analyses were based on observed data from 43 countries or regions that are located in 5 continents and with various climatic and socioeconomic conditions. The large data size and the various locations for urban settings strengthen the robustness of findings at the global scale.

This study also has some limitations. In this study, we built the models using 5 meta-predictors that have been used to explain certain heterogeneity in the mortality risks associated with heatwaves across locations [4,8,12]. However, previous research observed heterogeneity in health responses to extreme weather events (e.g., heatwaves), including differences in relation to greenspace, socioeconomics, urbanicity, and other individual and community characteristics [25]. We acknowledge the potential over or underestimations in somewhat finer resolution scales and that the populations and community characteristics vary dramatically in ways that could affect the relationship between heatwaves and mortality. This research limitation is expected to reduce if grid cell-specific mortality rate and other data (e.g., urbanicity, population characteristics) are being available in the future. We also had no access to daily death data from certain regions, e.g., Arabian Peninsula and South Asia. This may reduce the accuracy of findings in those regions. However, the prediction model may partly reduce the uncertainty by borrowing data from locations with similar temperature range, climate, and socioeconomic development.

To conclude, heatwave exposure was associated with substantial global burden of mortality, which varied from national to subnational levels, and changed in a complex temporal pattern. The findings call for action from local to intergovernmental local policy-makers to design effective adaptation and mitigation strategies to address climate change.

## Supporting information

S1 STROBE Checklist Strengthening the Reporting of Observational Studies in Epidemiology (STROBE) checklist. (DOCX)

S1 Text MCC collaborators. (DOCX)

S2 Text Updated information of the MCC data set. (DOCX)

S3 Text Data collection of annual GDP, population, and GDP per capita (0.5°×0.5°). (DOCX)

S4 Text Data collection of annual mortality rate per country in 1990–2019. (DOCX)

S5 Text Modelling grid cell-specific deaths (0.5°×0.5°) in 1990–2019 warm seasons. (DOCX)

S6 Text Heatwave definition. (DOCX)

S7 Text Explanation of the three-stage strategy (0.5°×0.5°). (DOCX)

S8 Text Long-term heat acclimation in the three-stage modelling. (DOCX)

S1 Table Basic characteristics of 750 MCC locations. (DOCX)

S2 Table Significance test for predictors (*p*-value) and I^2^ statistic (%) in multivariate random-effects meta-regression models. (DOCX)

S3 Table I^2^ statistic (%) in multivariate random-effects meta-regression models and significance test comparing the fitness between models. (DOCX)

S4 Table Grid cell-specific average daily mean temperature (with SD) during the warm season from 1990–1999 to 2010–2019 by continent and region. (DOCX)

S5 Table Grid cell-specific heatwave days with interquartile range (25th and 75th) during the warm season from 1990–1999 to 2010–2019 by continent and region. (DOCX)

S6 Table Overall and average annual excess deaths (based on country-specific population structure) associated with heatwaves in warm seasons from 1990–1999 to 2010–2019 by continent, region, and countries. (DOCX)

S7 Table Overall and average annual excess deaths (based on the age structure of WHO standard population) associated with heatwaves in warm seasons from 1990–1999 to 2010–2019 by continent, region, and countries. (DOCX)

S8 Table Average excess death ratio (%, based on country-specific population structure) associated with heatwaves per warm season from 1990–1999 to 2010–2019 by continent, region, and countries. (DOCX)

S9 Table Average excess death ratio (%, based on the age structure of WHO standard population) associated with heatwaves per warm season from 1990–1999 to 2010–2019 by continent, region, and countries. (DOCX)

S10 Table Average excess deaths per 10 million residents (based on country-specific population structure) associated with heatwaves per warm season from 1990–1999 to 2010–2019 by continent, region, and countries. (DOCX)

S11 Table Average excess deaths per 10 million residents (based on the age structure of WHO standard population) associated with heatwaves per warm season from 1990–1999 to 2010–2019 by continent, region, and countries. (DOCX)

S12 Table Average excess deaths (based on country-specific population structure) associated with heatwaves per warm season from 1990–1999 to 2010–2019 by the indicators of Köppen–Geiger climate classification and World Bank income groups. (DOCX)

S13 Table Average excess deaths (based on the age structure of WHO standard population) associated with heatwaves per warm season from 1990–1999 to 2010–2019 by the indicators of Köppen–Geiger climate classification and World Bank income groups. (DOCX)

S14 Table Average excess deaths per 10 million residents (based on country-specific population structure) associated with heatwaves per warm season from 1990–1999 to 2010–2019 by the indicators of Köppen–Geiger climate classification and World Bank income groups. (DOCX)

S15 Table Average excess deaths per 10 million residents (based on the age structure of WHO standard population) associated with heatwaves per warm season from 1990–1999 to 2010–2019 by the indicators of Köppen–Geiger climate classification and World Bank income groups. (DOCX)

S16 Table Average excess death ratio (based on country-specific population structure) associated with heatwaves per warm season from 1990–1999 to 2010–2019 by the indicators of Köppen–Geiger climate classification and World Bank income groups. (DOCX)

S17 Table Average excess death ratio (based on the age structure of WHO standard population) associated with heatwaves per warm season from 1990–1999 to 2010–2019 by the indicators of Köppen–Geiger climate classification and World Bank income groups. (DOCX)

S18 Table Relative risk (RR) of heatwave-related mortality with 95%confidence interval (CI) for each country from 1990–1999 to 2010–2019. (DOCX)

S19 Table Results of sensitivity analyses on the effect size of heatwave event in comparison to non-heatwave days. (DOCX)

S1 Fig Average and the change per decade of excess deaths (A, B), death ratio (%, C, D), and deaths per 10 million residents (E, F) (based on the age structure of WHO standard population) associated with heatwaves per warm season during 1990–2019 at a spatial resolution of 0.5°×0.5°.
(DOCX)

S2 Fig Leading 20 countries for excess deaths (based on country-specific population structure) associated with heatwaves per warm season from 1990–1999 to 2010–2019. (DOCX)

S3 Fig Leading 20 countries for excess deaths (based on the age structure of WHO standard population) associated with heatwaves per warm season from 1990–1999 to 2010–2019. (DOCX)

S4 Fig Leading 20 countries for excess death ratio (%) and deaths per 10 million residents (based on the age structure of WHO standard population) associated with heatwaves per warm season from 1990–1999 to 2010–2019. (DOCX)

## Abbreviations

df: degree of freedom
eCI: empirical confidence interval
GDP: gross domestic product
MCC: Multi-Country Multi-City
WHO: World Health Organization
