# Supplementary material for: Global, regional, and national burden of heatwave-related mortality from 1990 to 2019: A three-stage modelling study
Source: PLoS Med. 2024 May 14;21(5):e1004364. doi: 10.1371/journal.pmed.1004364 (PMC11093289; doi:10.1371/journal.pmed.1004364)
Supplement: S1 STROBE Checklist — (DOCX) [file pmed.1004364.s001.docx]

**S1 Strobe checklist.** Strengthening the Reporting of Observational Studies in Epidemiology (STROBE) checklist.

|  | Item No | Recommendation | Relevant content in this paper |
| --- | --- | --- | --- |
| **Title and abstract** | 1 | (*a*) Indicate the study’s design with a commonly used term in the title or the abstract | In Title: ‘A three-stage modelling study’ |
|  |  | (*b*) Provide in the abstract an informative and balanced summary of what was done and what was found | In ‘Methods and findings’ section |
| Introduction | | |  |
| Background/rationale | 2 | Explain the scientific background and rationale for the investigation being reported | In para 1-2, Introduction |
| Objectives | 3 | State specific objectives, including any prespecified hypotheses | In para 3, Introduction |
| Methods | | |  |
| Study design | 4 | Present key elements of study design early in the paper | In para 1, Methods: ‘a time-series modelling study’. Details of study design are presented in the Statistical analysis section, Methods. |
| Setting | 5 | Describe the setting, locations, and relevant dates, including periods of recruitment, exposure, follow-up, and data collection | In Methods: ‘This study used the dataset covering daily death counts for all causes or when such data unavailable, for non-external causes [International Classification of Diseases (ICD)-9: 0-799 or ICD-10: A00-R99] from 750 locations of 43 countries or regions (marked with black crosses in Fig 1A). The period of data collection overlapped largely, ranging from 1969 to 2018.’  The heatwave-mortality association was separately in the 750 locations, which was used with a series of predictors to calculate heatwave-related deaths across the globe at a spatial resolution of 0.5˚×0.5˚ between 1990 and 2019. |
| Participants | 6 | (*a*) *Cohort study*—Give the eligibility criteria, and the sources and methods of selection of participants. Describe methods of follow-up  *Case-control study*—Give the eligibility criteria, and the sources and methods of case ascertainment and control selection. Give the rationale for the choice of cases and controls  *Cross-sectional study*—Give the eligibility criteria, and the sources and methods of selection of participants | In Methods: ‘This study used the dataset covering daily death counts for all causes or when such data unavailable, for non-external causes [International Classification of Diseases (ICD)-9: 0-799 or ICD-10: A00-R99] from 750 locations of 43 countries or regions (Fig 1A). The period of data collection overlapped largely, ranging from 1969 to 2018.’  Details were provided in Text A of S2 Text. |
|  |  | (*b*) *Cohort study*—For matched studies, give matching criteria and number of exposed and unexposed  *Case-control study*—For matched studies, give matching criteria and the number of controls per case | NA  NA |
| Variables | 7 | Clearly define all outcomes, exposures, predictors, potential confounders, and effect modifiers. Give diagnostic criteria, if applicable | These are defined and presented in the ‘Data sources’ section, ‘Definition of heatwaves’ section and ‘Statistical analysis’ section, Methods. **Outcomes** are the grid cell-specific heatwave-related excess deaths, the ratio (%) between heatwave-related excess deaths and total deaths in the warm season (i.e., excess death ratio), and the heatwave-related excess deaths per ten million residents (i.e., excess death rate). **Exposures**, i.e. heatwaves are: ‘heatwaves were defined for each location as daily mean temperature ≥95th percentiles of year-round temperature range with duration ≥2 days.’ **Covariates** are ‘continent, Köppen–Geiger climate classification, GDP per capita, and the average and the range of daily mean temperature in the warm season’. |
| Data sources/ measurement | 8* | For each variable of interest, give sources of data and details of methods of assessment (measurement). Describe comparability of assessment methods if there is more than one group | These are presented in the ‘Data sources’ section, Methods. |
| Bias | 9 | Describe any efforts to address potential sources of bias | The empirical confidence intervals (eCIs) were calculated using Monte Carlo simulations (500 samples) to quantify the uncertainty in estimating the excess mortality burden by assuming a normal distribution for the coefficient of heatwave effect estimate. This study applied methodology (and parameters) that has been well justified by Previous MCC studies via a series of sensitivity analyses. In addition, a series of sensitivity analyses were performed to verify the robustness of modelling and findings |
| Study size | 10 | Explain how the study size was arrived at | Analyses were restricted to grid cells with at least one annual death, which in total accounted for 99.995% of the global population. |
| Quantitative variables | 11 | Explain how quantitative variables were handled in the analyses. If applicable, describe which groupings were chosen and why | These are presented in the ‘Data sources’ section, Methods. |
| Statistical methods | 12 | (*a*) Describe all statistical methods, including those used to control for confounding | In Statistical analysis section, Methods. |
|  |  | (*b*) Describe any methods used to examine subgroups and interactions | In Statistical analysis section, Methods. |
|  |  | (*c*) Explain how missing data were addressed | Analyses were based on daily death counts for all causes or when such data unavailable, for non-external causes [International Classification of Diseases (ICD)-9: 0-799 or ICD-10: A00-R99]. Explanation of missing data of predictors was provided in Text C of S2 Text. |
|  |  | (*d*) *Cohort study*—If applicable, explain how loss to follow-up was addressed  *Case-control study*—If applicable, explain how matching of cases and controls was addressed  *Cross-sectional study*—If applicable, describe analytical methods taking account of sampling strategy | NA |
|  |  | (*e*) Describe any sensitivity analyses | In Statistical analysis section, Methods and Table R in S2 Text. |

| Results | | |  |
| --- | --- | --- | --- |
| Participants | 13* | (a) Report numbers of individuals at each stage of study—eg numbers potentially eligible, examined for eligibility, confirmed eligible, included in the study, completing follow-up, and analysed | NA. This study only used aggregate anonymized data on daily deaths from 750 locations of 43 countries or regions. No individual participants were involved. |
|  |  | (b) Give reasons for non-participation at each stage | NA |
|  |  | (c) Consider use of a flow diagram | NA |
| Descriptive data | 14* | (a) Give characteristics of study participants (eg demographic, clinical, social) and information on exposures and potential confounders | In para 1, Results and Tables A, D, E in S2 Text |
|  |  | (b) Indicate number of participants with missing data for each variable of interest | NA |
|  |  | (c) *Cohort study*—Summarise follow-up time (eg, average and total amount) | NA |
| Outcome data | 15* | *Cohort study*—Report numbers of outcome events or summary measures over time | NA |
|  |  | *Case-control study—*Report numbers in each exposure category, or summary measures of exposure | NA |
|  |  | *Cross-sectional study—*Report numbers of outcome events or summary measures | In para 1, Results |
| Main results | 16 | (*a*) Give unadjusted estimates and, if applicable, confounder-adjusted estimates and their precision (eg, 95% confidence interval). Make clear which confounders were adjusted for and why they were included | In para 2, Results |
|  |  | (*b*) Report category boundaries when continuous variables were categorized | In para 1-3, Results, Tables 1-2, and Tables F-R in S2 Text. |
|  |  | (*c*) If relevant, consider translating estimates of relative risk into absolute risk for a meaningful time period | NA |
| Other analyses | 17 | Report other analyses done—eg analyses of subgroups and interactions, and sensitivity analyses | In para 1-4, Results |
| Discussion | | |  |
| Key results | 18 | Summarise key results with reference to study objectives | In para 1, Discussion |
| Limitations | 19 | Discuss limitations of the study, taking into account sources of potential bias or imprecision. Discuss both direction and magnitude of any potential bias | In para 9, Discussion |
| Interpretation | 20 | Give a cautious overall interpretation of results considering objectives, limitations, multiplicity of analyses, results from similar studies, and other relevant evidence | In para 2-9, Discussion |
| Generalisability | 21 | Discuss the generalisability (external validity) of the study results | In para 78, Discussion |
| Other information | | |  |
| Funding | 22 | Give the source of funding and the role of the funders for the present study and, if applicable, for the original study on which the present article is based | In Funding |

*Give information separately for cases and controls in case-control studies and, if applicable, for exposed and unexposed groups in cohort and cross-sectional studies.

**Note:** An Explanation and Elaboration article discusses each checklist item and gives methodological background and published examples of transparent reporting. The STROBE checklist is best used in conjunction with this article (freely available on the Web sites of PLoS Medicine at http://www.plosmedicine.org/, Annals of Internal Medicine at http://www.annals.org/, and Epidemiology at http://www.epidem.com/). Information on the STROBE Initiative is available at www.strobe-statement.org.
