# Supplementary material for: Global, regional, and national burden of heatwave-related mortality from 1990 to 2019: A three-stage modelling study
Source: PLoS Med. 2024 May 14;21(5):e1004364. doi: 10.1371/journal.pmed.1004364 (PMC11093289; doi:10.1371/journal.pmed.1004364)
Supplement: S1 Text — (DOCX) [file pmed.1004364.s002.docx]

**S1 Text. MCC collaborators**

Antonella Zanobetti^1^, Magali Hurtado Diaz^2^, Eunice Elizabeth Félix Arellano^2^, Micheline de Sousa Zanotti Stagliorio Coelho^3^, Patricia Matus Correa^4^, Nicolás Valdés Ortega^5^, Samuel Osorio^6^, Gabriel Carrasco^7^, Valentina Colistro^8^, Hans Orru^9^, Ene Indermitte^9^, Jouni J. K. Jaakkola^10,11^, Ariana Zeka^12^, Shilpa Rao^13^, Bertil Forsberg^14^, Klea Katsouyanni^15,16^, Antonis Analitis^15^, Evangelia Samoli^15^, Paola Michelozzi^17^, Massimo Stafoggia^17^, Susana das Neves Pereira da Silva^18^, Joana Madureira^19,20,21^, Carmen Íñiguez^22,23^, Alexandra Schneider^24^, Veronika Huber^24,25^ , Susanne Breitner^24,25^, Martina S. Ragettli^26,27^, Danny Houthuijs^28^, Caroline Ameling^28^, Jan Kyselý^29,30^, Ala Overcenco^31^, Iulian-Horia Holobaca^32^, Noah Scovronick^33^, Fiorella Acquaotta^34^, Fatemeh Mayvaneh^35^, Hematollah Roradeh^35^, Barrak Alahmad^1^, Yasushi Honda^36^, Masahiro Hashizume^37^, Chris Fook Sheng Ng^37^, Yoonhee Kim^38^, Ho Kim^39^, Whanhee Lee^40^, Xerxes Seposo^41,42^, Paul Lester Carlos Chua^37^, Tran Ngoc Dang^43^, Do Van Dung^44^, Yue Leon Guo^45,46,47^, Shih-Chun Pan^46^, Rosana Abrutzky^48^, Marek Maasikmets^49^, Rebecca M. Garland^50^, Pierre Masselot^51^

**Affiliations:**

^1^Department of Environmental Health, Harvard T.H. Chan School of Public Health, Boston, MA, USA;

^2^Department of Environmental Health, National Institute of Public Health, Cuernavaca, Morelos, Mexico;

^3^Department of Pathology, Faculty of Medicine, University of São Paulo, São Paulo, Brazil;

^4^Department of Public Health, Universidad de los Andes, Santiago, Chile;

^5^Centro Interdisciplinario de Cambio Global, Pontificia, Universidad Católica de Chile, Santiago, Chile;

^6^Department of Environmental Health, University of São Paulo, São Paulo, Brazil;

^7^Institute of Tropical Medicine "Alexander von Humboldt", Universidad Peruana Cayetano Heredia, Lima, Peru;

^8^Department of Quantitative Methods, School of Medicine, University of the Republic, Montevideo, Uruguay;

^9^Department of Family Medicine and Public Health, University of Tartu, Tartu, Estonia;

^10^Center for Environmental and Respiratory Health Research (CERH), University of Oulu, Oulu, Finland;

^11^Medical Research Center Oulu (MRC Oulu), Oulu University Hospital and University of Oulu, Oulu, Finland;

^12^UK Health Security Agency, London, UK;

^13^Norwegian institute of Public Health, Oslo, Norway;

^14^Department of Public Health and Clinical Medicine, Umeå University, Sweden;

^15^Department of Hygiene, Epidemiology and Medical Statistics, National and Kapodistrian University of Athens, Greece;

^16^Environmental Research Group, School of Public Health, Imperial College, London, UK;

^17^Department of Epidemiology, Lazio Regional Health Service, Asl Roma 1, Rome, Italy;

^18^Department of Epidemiology, Instituto Nacional de Saúde Dr. Ricardo Jorge, Lisbon, Portugal;

^19^Department of Environmental Health, Instituto Nacional de Saúde Dr. Ricardo Jorge, Porto, Portugal;

^20^EPIUnit - Instituto de Saúde Pública, Universidade do Porto, Porto, Portugal;

^21^Laboratório para a Investigação Integrativa e Translacional em Saúde Populacional (ITR), Porto, Portugal

^22^Department of Statistics and Computational Research, Universitat de València, València, Spain;

^23^CIBERESP, Madrid, Spain;

^24^Institute of Epidemiology, Helmholtz Zentrum München – German Research Center for Environmental Health (GmbH), Neuherberg, Germany

^25^IBE-Chair of Epidemiology, Faculty of Medicine, LMU Munich, Munich, Germany

^26^Swiss Tropical and Public Health Institute, Allschwil, Switzerland;

^27^University of Basel, Basel;

^28^National Institute for Public Health and the Environment (RIVM), Centre for Sustainability and Environmental Health, Bilthoven, Netherlands;

^29^Institute of Atmospheric Physics, Academy of Sciences of the Czech Republic, Prague, Czech Republic;

^30^Faculty of Environmental Sciences, Czech University of Life Sciences, Prague, Czech Republic;

^31^National Agency for Public Health of the Ministry of Health, Labour and Social Protection of the Republic of Moldova;

^32^Faculty of Geography, Babes-Bolay University, Cluj-Napoca, Romania

^33^Department of Environmental Health, Rollins School of Public Health, Emory University, Atlanta, USA

^34^Department of Earth Sciences, University of Torino, Italy

^35^Geography and Urban Planning Department, University of Mazandaran, Babolsar, Iran

^36^Center for Climate Change Adaptation, National Institute for Environmental Studies, Tsukuba, Japan

^37^Department of Global Health Policy, Graduate School of Medicine, The University of Tokyo, Tokyo, Japan

^38^Department of Global Environmental Health, Graduate School of Medicine, University of Tokyo, Tokyo, Japan

^39^Graduate School of Public Health, Seoul National University, Seoul, South Korea

^40^School of Biomedical Convergence Engineering, College of Information and Biomedical Engineering, Pusan National University, Yangsan, South Korea

^41^Department of Hygiene, Graduate School of Medicine, Hokkaido University, Sapporo, Japan;

^42^School of Tropical Medicine and Global Health, Nagasaki University, Nagasaki, Japan;

^43^Department of Environmental Health, Faculty of Public Health, Department of Environmental Health, University of Medicine and Pharmacy at Ho Chi Minh City, Ho Chi Minh City, Vietnam;

^44^Department of Environmental Health, Faculty of Public Health, University of Medicine and Pharmacy at Ho Chi Minh City, Ho Chi Minh City, Vietnam;

^45^Environmental and Occupational Medicine, National Taiwan University (NTU) College of Medicine and NTU Hospital, Taipei, Taiwan;

^46^National Institute of Environmental Health Science, National Health Research Institutes, Zhunan, Taiwan;

^47^Graduate Institute of Environmental and Occupational Health Sciences, NTU College of Public Health, Taipei, Taiwan;

^48^Universidad de Buenos Aires, Facultad de Ciencias Sociales, Instituto de Investigaciones Gino Germani;

^49^Estonian Environmental Research Centre, Tallinn, Estonia;

^50^Department of Geography, Geoinformatics and Meteorology, University of Pretoria, Pretoria, South Africa;

^51^Environment & Health Modelling (EHM) Lab, Department of Public Health Environments and Society, London School of Hygiene & Tropical Medicine, London, UK
