# Supplementary material for: Global, regional, and national burden of heatwave-related mortality from 1990 to 2019: A three-stage modelling study
Source: PLoS Med. 2024 May 14;21(5):e1004364. doi: 10.1371/journal.pmed.1004364 (PMC11093289; doi:10.1371/journal.pmed.1004364)
Supplement: S2 Text — (DOCX) [file pmed.1004364.s003.docx]

# **S2 Text.** Updated information of the MCC data set

The Multi-Country Multi-City (MCC) Collaborative Research Network has been collecting time-series data on daily deaths and environmental variables (e.g. daily mean temperature, relative humidity and air pollution) since 2014. It is an international collaboration of research teams working on a program aiming to produce epidemiological evidence on associations between environmental stressors, climate, and health (<http://mccstudy.lshtm.ac.uk/>). The earliest MCC data set collected data from 306 locations in 12 countries/territories: Australia (3 cities during 1988–2008), Brazil (18 cities during 1997–2011), Thailand (62 provinces during 1999–2008), mainland China (6 cities during 2002–2011), Taiwan (3 cities during 1994–2007), South Korea (7 cities during 1992–2010), Japan (7 cities during 1972–2009), Italy (10 cities during 1987–2010), Spain (51 cities during 1990–2010), United Kingdom (10 regions during 1993–2006), United States (108 cities during 1987–2000), and Canada (21 cities during 1986–2009).

The MCC Collaborative Research Network has developed during the years, with the data set extended frequently. In this version of data set, data from 1364 locations in 43 countries/territories during different periods has been included. In this study, the data set with more locations or more recent data set is selected for each country. After removing data from 612 locations due to duplication and two locations due to quality issue, a total of 130,217,521 deaths from 750 locations in 43 countries or territories are finally included in the analysis. In summary, 590 and 335 out of the 750 locations had death data for all causes and non-external causes, respectively. In addition, 175 out of the 750 locations had both death data. The longitudes and latitudes of the 750 locations vary between -157.9 to 153.0 and -38.7 to 61.2, respectively. Basic characteristics of these locations have been summarized in Supplementary Table 1 by country or territory.
