# Supplementary material for: Global, regional, and national burden of heatwave-related mortality from 1990 to 2019: A three-stage modelling study
Source: PLoS Med. 2024 May 14;21(5):e1004364. doi: 10.1371/journal.pmed.1004364 (PMC11093289; doi:10.1371/journal.pmed.1004364)
Supplement: S3 Text — (DOCX) [file pmed.1004364.s004.docx]

# **S3 Text.** Data collection of annual GDP, population, and GDP per capita (0.5˚×0.5˚)

In this study, the yearly data on Gross Domestic Product (GDP) (calibrated to the 2005 inflation rate) and population size were provided in 1990, 2000, 2010, and 2020 by the Global Carbon Project [1]. The annual data between each two time slots, e.g., 1990 and 2000, were interpolated linearly for each grid cell. The Global Carbon Project estimated the GDP and population per grid cell by downscaling observed population and GDP by country, while those in 2020 were estimated by downscaling projected data under three shared socioeconomic pathways (SSP) –SSP1, SSP2, and SSP3, by country. The final GDP and population data in 2020 were calculated by averaging the projections under SSP1-3. Annual GDP per capita for each grid cell was calculated using the interpolated GDP and population. For each of the 750 locations, the average GDP per capita during the data collection period was calculated using the interpolated GDP and population in the middle year of the data collection period from the grid cell where the central coordinate of this location was located in.

**Reference**

1. Murakami D, Yamagata Y. Estimation of gridded population and GDP scenarios with spatially explicit statistical downscaling. Sustainability. 2019;11(7):2106.
