# Supplementary material for: Global, regional, and national burden of heatwave-related mortality from 1990 to 2019: A three-stage modelling study
Source: PLoS Med. 2024 May 14;21(5):e1004364. doi: 10.1371/journal.pmed.1004364 (PMC11093289; doi:10.1371/journal.pmed.1004364)
Supplement: S4 Text — (DOCX) [file pmed.1004364.s005.docx]

# **S4 Text.** Data collection of annual mortality rate per country in 1990–2019

The annual data on age-specific population size and death for each country between 1990 and 2019 were collected from the Global Burden of Disease Study 2019 (GBD 2019) [1], which were used to calculate the crude mortality rate and the age-standardized mortality rate. To reduce uncertainty due to the use of estimated data on age-specific population size and death, the age-standardized mortality rate was calculated using five age-groups (0-4yr, 5-14yr, 15-49yr, 50-69yr and 70+yr). There were three countries or regions without data on age-specific population size and death between 1990 and 2019, where the annual mortality rates were replaced using the average annual mortality rate of the same World Bank income-level groups (low, lower-middle, upper-middle, and high-income countries). These three countries or regions, i.e., Holy See (Vatican City State), Liechtenstein and Western Sahara, accounted for 0.00572% of the world’s population size in 2010, which should have minimal effect on the main results.

**Reference**

1. GBD 2019 Demographics Collaborators. Global age-sex-specific fertility, mortality, healthy life expectancy (HALE), and population estimates in 204 countries and territories, 1950-2019: a comprehensive demographic analysis for the Global Burden of Disease Study 2019. Lancet (London, England). 2020;396(10258):1160-203. Epub 2020/10/19. doi: 10.1016/s0140-6736(20)30977-6. PubMed PMID: 33069325; PubMed Central PMCID: PMCPMC7566045.
