# Supplementary material for: Global, regional, and national burden of heatwave-related mortality from 1990 to 2019: A three-stage modelling study
Source: PLoS Med. 2024 May 14;21(5):e1004364. doi: 10.1371/journal.pmed.1004364 (PMC11093289; doi:10.1371/journal.pmed.1004364)
Supplement: S5 Text — (DOCX) [file pmed.1004364.s006.docx]

# **S5 Text.** Modelling grid cell-specific deaths (0.5˚×0.5˚) in 1990–2019 warm seasons

Annual deaths in the warm season for each grid cell was calculated based on the grid cell-specific annual mortality rate, the ratio between warm-season and year-round deaths (seasonal ratio for short), and the annual population size. Briefly, the grid cell-specific annual mortality rate was replaced using the country-specific rate where this grid cell was located in, assuming a same rate across grids in this country. The grid cell-specific seasonal ratio was calculated via the following two-stage strategy: First, the seasonal ratio was calculated for each of the 750 locations. A linear model was built between the season ratio and location-specific predictors during the data collection period, including continent, GDP per capita, indicators for Köppen–Geiger climate classification, the yearly average and the range of daily mean temperature, and the seasonal average and the range of daily mean temperature in the warm season. Second, the grid cell-specific seasonal ratio was predicted using the model built and the grid cell-specific predictors. In this study, we assumed the temporal consistency in grid cell-specific seasonal ratio from 1990 to 2019.
