# Supplementary material for: Global, regional, and national burden of heatwave-related mortality from 1990 to 2019: A three-stage modelling study
Source: PLoS Med. 2024 May 14;21(5):e1004364. doi: 10.1371/journal.pmed.1004364 (PMC11093289; doi:10.1371/journal.pmed.1004364)
Supplement: S6 Text — (DOCX) [file pmed.1004364.s007.docx]

# **S6 Text.** Heatwave definition

Heatwave is usually defined as a few consecutive days with high temperatures above a certain threshold while currently there is no standard definition worldwide by numerous studies and documents. The temperature threshold can be either physiologically based (absolute threshold) or location based (relative threshold). Most studies covering large areas commonly applied certain relative temperature threshold based on each location’s long-term daily mean temperature, as this definition considers local climate acclimatization. To examine the adverse effect of intensity of heatwaves, the relative thresholds were commonly set at the daily mean temperatures of 90th, 92.5th, 95th, 97.5th, or even 99th percentiles of each location’s year-round temperature distribution. To examine the adverse effect of duration of heatwaves, durations of ≥2 (two or more adjacent days), 3 (three or more adjacent days), or 4d (four or more adjacent days) have been applied by previous studies to define heatwaves.

For the intensity of heatwaves, we initially applied 95th, 97.5th and 99th percentiles of each location’s year-round temperature distribution, considering their relatively extreme heat. For the duration of heatwave, we initially considered the duration of ≥2, ≥3 and ≥4 days. Namely, nine types of heatwaves were considered to use in total. However, the modeling for certain grid cells failed using the heatwaves defined by combining duration of ≥3 days and the three intensities (95th, 97.5th and 99th percentiles) due to very limited events in certain years (caused by climate fluctuation). As a result, we finally defined heatwave events across the gird cells worldwide using the duration of ≥2 days. The median numbers of heatwave days per year were 12 days for the definition combining 95th percentile temperature and ≥2 days, 4 days for the definition combining 975th percentile temperature and ≥2 days, and 0 days for the definition combining 99th percentile temperature and ≥2 days, respectively across global grid cells. To improve modeling stability, we finally decided to define the heatwave for each location as daily mean temperature ≥95th percentiles of year-round temperature range with duration ≥2 days.
