# Supplementary material for: Global, regional, and national burden of heatwave-related mortality from 1990 to 2019: A three-stage modelling study
Source: PLoS Med. 2024 May 14;21(5):e1004364. doi: 10.1371/journal.pmed.1004364 (PMC11093289; doi:10.1371/journal.pmed.1004364)
Supplement: S7 Text — (DOCX) [file pmed.1004364.s008.docx]

# **S7 Text.** Explanation of the three-stage strategy (0.5˚×0.5˚)

In this study, we estimated the heatwave-related mortality burden at a spatial resolution of 0.5˚×0.5˚. The key methodological innovation of this study is to predict the heatwave-mortality association for areas without daily time-series mortality data. Numerous studies have indicated a strong geographic variation in the association between non-optimal temperatures (including heatwaves) and health outcomes, which is linked with climatic, geographic, socio-economic and demographic factors of each location [1-3]. This observation provides the theoretical solution for predicting heatwave-mortality association in areas without daily mortality data. We applied a three-stage analysis strategy to achieve this:

First stage: We estimated the heatwave-mortality association for each of the 750 MCC locations.

Second stage: We collected location-specific predictors that could explain the majority of heterogeneity in the heatwave-mortality associations across the 750 locations. We selected predictors (the continents, indicators for Köppen–Geiger climate classification, GDP per capita, and the average and the range of daily mean temperature in the warm season). We then built a meta-regression between location-specific association and those location-specific predictors. Continents included Americas, Europe, Africa, Asia and Oceania. Indicators for Köppen-Geiger climate classification had five categories: group A for tropical climates, group B for dry climates, group C for temperate climates, group D for continental climates, group E for polar and alpine climates.

Third stage: We collected data on predictors aforementioned at the grid cell level and used the coefficients of each predictor from the meta-regression of the second stage to predict the grid cell-specific heatwave-mortality association. The grid cell-specific excess deaths associated with heatwave were calculated under two mortality scenarios, i.e., by using the real country-specific mortality rate and by adjusting country-specific mortality by the age distribution of WHO standard population.

Additional explanation for the predictor selection in the second stage: We applied a parsimonious strategy for predictor selection, i.e., to minimize the number of investigated predictors, considering the higher chance of overfitting for meta-regression that conventional regression models. Briefly, two steps were performed. In the first step, we selected five predictors in the meta-regression model, including the continents, Köppen–Geiger climate classification, GDP per capita, the average and the range of daily mean temperature in warm season. These variables were selected considering they (1) belonged to various fields of geography, climate, meteorology, and socio-economy; (2) had been recommended by literature and our previous studies as major effect modifiers for the effect size of heatwave across locations [4-6]. Both single-predictor and full models of meta-regression suggested their statistical significance (P values <0.05, Supplementary table 2). The I^2^ value declined from 61.2% for the meta-analysis (with intercept only) to 47.5% for the meta-regression of the five predictors across the 750 locations, suggesting the good performance of our model in reducing inter-location heterogeneity (Supplementary table 2). In the second step, more potential effect modifiers were considered to test the reliability of the meta-regression model built in the first step. These potential effect modifiers belonged to the fields of climate and meteorology (variability of daily mean temperature in warm season), socio-disparity (GINI coefficient), demography (proportion of elder population ≥65 years), public health (life expectancy), and urbanization (proportion of population living in urban area). Data were also collected from the World Bank (<https://data.worldbank.org/indicator>). First, the multi-collinearity among continuous variables was evaluated by calculating intercorrelation matrix. Variables with moderate-to-high correlations (r > 0.7) to the continuous variables in the first step were removed, including variability of daily mean temperature in warm season, GINI coefficient and life expectancy. Second, all rest variables passed the correlation analysis (i.e., proportion of elder population and urban population) were entered into the multivariate meta-regression. The I^2^ value declined slightly from 47.5% to 46.3% (Supplementary table 3). Likelihood ratio test suggest insignificant difference between the model fitted using the five predictors in the first step and the model fitted by additionally considering two variables in the second step (P>0.05, Supplementary table 3).

Thus, the meta-regression model built using these five predictors should be reliable.

Additional explanation for the third stage: The aim of calculating heatwave-related mortality burden using mortality rate adjusted by the age structure of WHO standard population was to roughly evaluate the impact of heatwave across regions after controlling for population ageing. The results should not be fully interpreted as the traditional ‘age-standardized mortality burden’ that calculated based on age-specific association between heatwave and mortality.

**Reference**

1. Gasparrini A, Armstrong B, Kenward MG. Multivariate meta‐analysis for non‐linear and other multi‐parameter associations. Statistics in medicine. 2012;31(29):3821-39.

2. McMichael AJ, Wilkinson P, Kovats RS, Pattenden S, Hajat S, Armstrong B, et al. International study of temperature, heat and urban mortality: the ‘ISOTHURM’project. Int J Epidemiol. 2008;37(5):1121-31.

3. Lee JY, Kim H, Gasparrini A, Armstrong B, Bell ML, Sera F, et al. Predicted temperature-increase-induced global health burden and its regional variability. Environ Int. 2019;131:105027.

4. Gasparrini A, Guo Y, Hashizume M, Lavigne E, Zanobetti A, Schwartz J, et al. Mortality risk attributable to high and low ambient temperature: a multicountry observational study. The Lancet. 2015;386(9991):369-75.

5. Gasparrini A, Guo Y, Sera F, Vicedo-Cabrera AM, Huber V, Tong S, et al. Projections of temperature-related excess mortality under climate change scenarios. Lancet Planet Health. 2017;1(9):e360-e7.

6. Zhao Q, Guo Y, Ye T, Gasparrini A, Tong S, Overcenco A, et al. Global, regional, and national burden of mortality associated with non-optimal ambient temperatures from 2000 to 2019: a three-stage modelling study. 2021;5(7):e415-e25.
