# Supplementary material for: Global, regional, and national burden of heatwave-related mortality from 1990 to 2019: A three-stage modelling study
Source: PLoS Med. 2024 May 14;21(5):e1004364. doi: 10.1371/journal.pmed.1004364 (PMC11093289; doi:10.1371/journal.pmed.1004364)
Supplement: S8 Text — (DOCX) [file pmed.1004364.s009.docx]

# **S8 Text.** Long-term heat acclimation in the three-stage modelling

Some studies modeled the health burden associated with heatwaves and other extreme temperature events by assuming an unchanged strength in the association [1, 2]. This strategy is plausible to capturing the temperature-related health burden on average in a short study period. However, there is increasing evidence that suggests a decline in the heatwave-health association due to the long-term acclimation to the local climate [3-5]. As a result, the consistent heatwave-health association may overestimate the related disease burden and its temporal change throughout a long period such as 30 years. It is a challenge to estimate the accurate change pattern in the heatwave-related disease burden for each grid cell. We considered the long-term heat acclimation by using a time-varied strategy. First, “adaptation” of heatwave definitions, i.e., the heatwaves were defined for each decade, considering temperature values of defining heatwaves in the early years may be different from the temperature values in later years. Second, “adaptation” of the association strength, i.e., the heatwave-mortality association for each grid cell was predicted for each decade by using meta-predictors in the same decade. Third, changes in mortality rate and population size were also considered during the calculation of excess deaths, death ratio and death rate. These steps improved the accuracy in estimating the mean heatwave-related mortality burden for each decade. However, time variation within each decade may still exist, particularly considering extreme fluctuation of climate in a single year. To simplify this issue, the temporal change from 1990 to 2019 was estimated as the % change per decade in comparison to the 1990–2019 average.

**Reference**

1. Liu J, Varghese BM, Hansen A, Zhang Y, Driscoll T, Morgan G, et al. Heat exposure and cardiovascular health outcomes: a systematic review and meta-analysis. The Lancet Planetary Health. 2022;6(6):e484-e95.

2. Son J-Y, Liu JC, Bell ML. Temperature-related mortality: a systematic review and investigation of effect modifiers. Environmental Research Letters. 2019;14(7):073004.

3. Gasparrini A, Guo Y, Hashizume M, Kinney PL, Petkova EP, Lavigne E, et al. Temporal variation in heat–mortality associations: a multicountry study. 2015;123(11):1200-7.

4. Lee W, Choi HM, Lee JY, Kim DH, Honda Y, Kim H. Temporal changes in mortality impacts of heat wave and cold spell in Korea and Japan. Environment international. 2018;116:136-46.

5. Sheridan SC, Allen MJ. Temporal trends in human vulnerability to excessive heat. Environmental research letters. 2018;13(4):043001.
