# Supplementary material for: Global, regional, and national burden of heatwave-related mortality from 1990 to 2019: A three-stage modelling study
Source: PLoS Med. 2024 May 14;21(5):e1004364. doi: 10.1371/journal.pmed.1004364 (PMC11093289; doi:10.1371/journal.pmed.1004364)
Supplement: S1 Table — (DOCX) [file pmed.1004364.s010.docx]

**S1 Table.** Basic characteristics of 750 MCC locations.

|  |  | **Country** | **No. of locations** | **Period of data collection** | **Coverage of the study period** | | | |
| --- | --- | --- | --- | --- | --- | --- | --- | --- |
|  |  |  |  |  | **1980-1989** | **1990-1999** | **2000-2009** | **2010–2019** |
| Americas | North America | Canada | 26 | 1986–2015 | √ | √ | √ | √ |
|  |  | United States | 211 | 1973–2006 | √ | √ | √ |  |
|  | Latin American and Caribbean | Guatemala | 1 | 2009–2016 |  |  | √ | √ |
|  |  | Mexico | 10 | 1998–2014 |  | √ | √ | √ |
|  |  | Panama | 1 | 2013–2016 |  |  |  | √ |
|  |  | Puertorico | 1 | 2009–2016 |  |  | √ | √ |
|  |  | Argentina | 3 | 2005–2015 |  |  | √ | √ |
|  |  | Brazil | 18 | 1997–2011 |  | √ | √ | √ |
|  |  | Chile | 4 | 1986–2015 | √ | √ | √ | √ |
|  |  | Colombia | 5 | 1998–2013 |  | √ | √ | √ |
|  |  | Costa Rica | 1 | 2000–2017 |  |  | √ | √ |
|  |  | Ecuador | 2 | 2014–2018 |  |  |  | √ |
|  |  | Paraguay | 1 | 2004–2016 |  |  | √ | √ |
|  |  | Peru | 18 | 2008–2014 |  |  | √ | √ |
|  |  | Uruguay | 1 | 2012–2016 |  |  |  | √ |
| Europe | Northern Europe | Estonia | 5 | 1997–2015 |  | √ | √ | √ |
|  |  | Finland | 1 | 1994–2014 |  | √ | √ | √ |
|  |  | Ireland | 6 | 1984–2007 | √ | √ | √ |  |
|  |  | Norway | 1 | 1969–2016 | √ | √ | √ | √ |
|  |  | Sweden | 3 | 1990–2016 |  | √ | √ | √ |
|  |  | United Kingdom | 70 | 1990–2016 |  | √ | √ | √ |
|  | Southern Europe | Greece | 1 | 2001–2010 |  |  | √ | √ |
|  |  | Italy | 18 | 2006–2015 |  |  | √ | √ |
|  |  | Portugal | 5 | 1980–2018 | √ | √ | √ | √ |
|  |  | Spain | 52 | 1990–2014 |  | √ | √ | √ |
|  | Western Europe | France | 18 | 2000–2014 |  |  | √ | √ |
|  |  | Germany | 12 | 1993–2015 |  | √ | √ | √ |
|  |  | Switzerland | 8 | 1995–2013 |  | √ | √ | √ |
|  |  | Netherlands | 5 | 1995–2016 |  | √ | √ | √ |
|  | Eastern Europe | Czech Republic | 4 | 1994–2015 |  | √ | √ | √ |
|  |  | Moldova | 4 | 2001–2010 |  |  | √ | √ |
|  |  | Romania | 8 | 1994–2016 |  | √ | √ | √ |
| Africa | Northern Africa | – | – | – | – | – | – | – |
|  | Sub-Saharan Africa | South Africa | 52 | 1997–2013 |  | √ | √ | √ |
| Asia | Central Asia | – | – | – | – | – | – | – |
|  | Southern Asia | Iran | 1 | 2004–2013 |  |  | √ | √ |
|  | Western Asia | Kuwait | 1 | 2000–2016 |  |  | √ | √ |
|  | Eastern Asia | China | 18 | 1994–2015 |  | √ | √ | √ |
|  |  | Japan | 47 | 1972–2015 | √ | √ | √ | √ |
|  |  | South Korea | 36 | 1997–2018 |  | √ | √ | √ |
|  | South-eastern Asia | Philippines | 4 | 2006–2010 |  |  | √ | √ |
|  |  | Thailand | 62 | 1999–2008 |  | √ | √ |  |
|  |  | Vietnam | 2 | 2009–2013 |  |  | √ | √ |
| Oceania | Australia and New Zealand | Australia | 3 | 1988–2009 | √ | √ | √ |  |
|  | Other regions in Oceania | – | – | – | – | – | – | – |
