# Supplementary material for: Global, regional, and national burden of heatwave-related mortality from 1990 to 2019: A three-stage modelling study
Source: PLoS Med. 2024 May 14;21(5):e1004364. doi: 10.1371/journal.pmed.1004364 (PMC11093289; doi:10.1371/journal.pmed.1004364)
Supplement: S2 Table — (DOCX) [file pmed.1004364.s011.docx]

# **S2 Table.** Significance test for predictors (p-value) and I^2^ statistic (%) in multivariate random-effects meta-regression models.

| Model | Predictor | P value for test for predictor | I^2^ value |
| --- | --- | --- | --- |
| Intercept-only | - | - | 61.2% |
| Single predictor | Continent | 0.0014 | 52.9% |
|  | Köppen–Geiger climate classification | <0.001 | 60.8% |
|  | GDP per capita | <0.001 | 60.7% |
|  | Average daily mean temperature in warm season | <0.001 | 59.6% |
|  | Range of daily mean temperature in warm season | <0.001 | 59.7% |
| Full model | Continent | <0.001 | 47.5% |
|  | Köppen–Geiger climate classification | <0.001 |  |
|  | GDP per capita | 0.047 |  |
|  | Average daily mean temperature in warm season | <0.001 |  |
|  | Range of daily mean temperature in warm season | <0.001 |  |
