# Supplementary material for: Global, regional, and national burden of heatwave-related mortality from 1990 to 2019: A three-stage modelling study
Source: PLoS Med. 2024 May 14;21(5):e1004364. doi: 10.1371/journal.pmed.1004364 (PMC11093289; doi:10.1371/journal.pmed.1004364)
Supplement: S3 Table — (DOCX) [file pmed.1004364.s012.docx]

# **S3 Table.** I^2^ statistic (%) in multivariate random-effects meta-regression models and significance test comparing the fitness between models.

| Model | Predictor | I^2^ value | P value for test inter-model difference |
| --- | --- | --- | --- |
| Full model | Five predictors | 47.5% | Reference |
| Extended model 1 | Five predictors+ proportion of elder population | 46.8% | 0.41 |
| Extended model 2 | Five predictors+ proportion of urban population | 46.5% | 0.08 |
| Extended model 3 | Five predictors+ proportion of elder population+ proportion of urban population | 46.3% | 0.17 |
