# Supplementary material for: Global, regional, and national burden of heatwave-related mortality from 1990 to 2019: A three-stage modelling study
Source: PLoS Med. 2024 May 14;21(5):e1004364. doi: 10.1371/journal.pmed.1004364 (PMC11093289; doi:10.1371/journal.pmed.1004364)
Supplement: S4 Table — (DOCX) [file pmed.1004364.s013.docx]

# **S4 Table.** Grid cell-specific average daily mean temperature (with SD) during the warm season from 1990–1999 to 2010–2019 by continent and region.

|  | **1990-1999** | **2000-2009** | **2010–2019** | **Change per decade ^a^** |
| --- | --- | --- | --- | --- |
| Global | 21.74±6.94 | 22.11±6.88 | 22.45±6.88 | 0.35 |
| Americas | 20.73±6.44 | 20.98±6.45 | 21.36±6.47 | 0.31 |
| Northern America | 17.38±5.52 | 17.64±5.49 | 18.08±5.58 | 0.35 |
| Latin American and Caribbean | 23.72±5.69 | 23.97±5.74 | 24.30±5.76 | 0.29 |
| Europe | 14.96±4.07 | 15.51±3.98 | 15.90±4.18 | 0.47 |
| Northern Europe | 12.07±3.71 | 12.70±3.55 | 12.73±3.63 | 0.33 |
| Southern Europe | 20.78±3.03 | 21.24±3.1 | 21.78±2.98 | 0.50 |
| Western Europe | 17.94±3.69 | 18.38±3.63 | 18.76±3.60 | 0.41 |
| Eastern Europe | 14.53±3.28 | 15.09±3.21 | 15.54±3.45 | 0.50 |
| Africa | 27.10±3.76 | 27.28±3.98 | 27.56±3.94 | 0.23 |
| Northern Africa | 29.22±3.10 | 29.86±3.12 | 29.96±3.11 | 0.37 |
| Sub-Saharan Africa | 26.56±3.72 | 26.61±3.91 | 26.94±3.89 | 0.19 |
| Asia | 24.21±6.46 | 24.68±6.39 | 24.97±6.40 | 0.38 |
| Central Asia | 21.30±4.61 | 21.71±4.54 | 22.27±4.77 | 0.48 |
| Southern Asia | 27.44±6.04 | 27.98±6.09 | 27.98±6.00 | 0.27 |
| Western Asia | 28.37±5.93 | 29.09±5.84 | 29.71±5.80 | 0.67 |
| Eastern Asia | 19.19±5.68 | 19.81±5.59 | 20.00±5.58 | 0.40 |
| South-eastern Asia | 27.24±1.77 | 27.31±1.79 | 27.63±1.84 | 0.19 |
| Oceania | 22.32±4.84 | 22.84±4.84 | 23.23±4.76 | 0.45 |
| Australia and New Zealand | 20.02±4.80 | 20.48±4.74 | 20.84±4.56 | 0.41 |
| Other regions in Oceania^b^ | 25.87±1.83 | 26.48±1.75 | 26.93±1.78 | 0.53 |

^a^ Change per decade is calculated using a linear regression.

^b^ Other regions in Oceania are defined as all areas outside of Australia and New Zealand in Oceania. All other regions in the table are defined according to the UN Statistics Division (M49) regional groupings
