# Supplementary material for: Global, regional, and national burden of heatwave-related mortality from 1990 to 2019: A three-stage modelling study
Source: PLoS Med. 2024 May 14;21(5):e1004364. doi: 10.1371/journal.pmed.1004364 (PMC11093289; doi:10.1371/journal.pmed.1004364)
Supplement: S6 Table — (DOCX) [file pmed.1004364.s015.docx]

**S6 Table.** Overall and average annual excess deaths (based on country-specific population structure) associated with heatwaves in warm seasons from 1990–1999 to 2010–2019 by continent, region, and countries. eCIs=empirical CIs. For country-specific data: Countries were summarized for the number of average annual excess deaths from high to low, and only those among the top 80^th^ were showed.

|  | **Overall** | **Average** | **1990-1999** | **2000-2009** | **2010–2019** | **%Change per decade ^a^** |
| --- | --- | --- | --- | --- | --- | --- |
| **Global** | 4592326 (3298499 to 5826823) | 153078 (109950 to 194227) | 149170 (109274 to 192453) | 145927 (105483 to 185516) | 164136 (115093 to 204443) | 4.89 |
| **Americas** | 246822 (122786 to 367202) | 8227 (4093 to 12240) | 7794 (3881 to 11629) | 7460 (3744 to 11236) | 9429 (4653 to 13855) | 9.94 |
| **Northern America** | 144684 (83562 to 204806) | 4823 (2785 to 6827) | 4680 (2686 to 6587) | 4313 (2509 to 6246) | 5476 (3159 to 7644) | 8.25 |
| Canada | 3969 (-954 to 8945) | 132 (-32 to 298) | 126 (-26 to 277) | 125 (-36 to 275) | 146 (-33 to 342) | 7.58 |
| United States | 140715 (84412 to 195877) | 4690 (2814 to 6529) | 4554 (2718 to 6310) | 4188 (2537 to 5971) | 5329 (3187 to 7307) | 8.26 |
| **Latin American and Caribbean** | 102138 (39327 to 163550) | 3405 (1311 to 5452) | 3114 (1174 to 5079) | 3147 (1250 to 5026) | 3953 (1509 to 6250) | 12.32 |
| Argentina | 10304 (5175 to 15066) | 343 (172 to 502) | 270 (147 to 424) | 347 (177 to 515) | 413 (194 to 567) | 20.85 |
| Bolivia | 1202 (-999 to 3542) | 40 (-33 to 118) | 46 (-34 to 129) | 33 (-28 to 98) | 41 (-38 to 127) | -6.25 |
| Brazil | 38195 (16960 to 57653) | 1273 (565 to 1922) | 1100 (518 to 1761) | 1224 (514 to 1786) | 1496 (662 to 2220) | 15.55 |
| Colombia | 2928 (392 to 5344) | 98 (13 to 178) | 94 (18 to 175) | 77 (7 to 139) | 122 (14 to 221) | 14.29 |
| Costa Rica | 455 (126 to 779) | 15 (4 to 26) | 10 (2 to 16) | 12 (3 to 20) | 24 (7 to 42) | 46.67 |
| Cuba | 2972 (1351 to 4865) | 99 (45 to 162) | 104 (43 to 154) | 88 (40 to 142) | 106 (52 to 189) | 1.01 |
| Dominican Republic | 1198 (422 to 2011) | 40 (14 to 67) | 29 (9 to 44) | 47 (18 to 83) | 44 (15 to 74) | 18.75 |
| Ecuador | 807 (81 to 1549) | 27 (3 to 52) | 24 (2 to 44) | 19 (3 to 38) | 38 (3 to 73) | 25.93 |
| Guatemala | 1256 (59 to 2377) | 42 (2 to 79) | 51 (3 to 99) | 35 (2 to 64) | 40 (1 to 74) | -13.10 |
| Honduras | 940 (369 to 1518) | 31 (12 to 51) | 32 (13 to 51) | 28 (11 to 45) | 34 (13 to 57) | 3.23 |
| Haiti | 2229 (1095 to 3509) | 74 (36 to 117) | 61 (28 to 88) | 104 (52 to 169) | 58 (29 to 93) | -2.03 |
| Jamaica | 576 (199 to 934) | 19 (7 to 31) | 16 (6 to 27) | 20 (7 to 32) | 21 (7 to 35) | 13.16 |
| Mexico | 26222 (13970 to 38589) | 874 (466 to 1286) | 875 (438 to 1218) | 750 (437 to 1153) | 998 (522 to 1487) | 7.04 |
| Nicaragua | 1039 (608 to 1555) | 35 (20 to 52) | 32 (17 to 43) | 34 (19 to 49) | 38 (24 to 63) | 8.57 |
| Panama | 479 (212 to 730) | 16 (7 to 24) | 12 (6 to 20) | 12 (5 to 19) | 23 (10 to 34) | 34.38 |
| Peru | 2627 (-2507 to 7971) | 88 (-84 to 266) | 115 (-94 to 314) | 66 (-63 to 214) | 82 (-93 to 269) | -18.75 |
| Paraguay | 1453 (985 to 1855) | 48 (33 to 62) | 36 (25 to 48) | 45 (32 to 60) | 64 (42 to 78) | 29.17 |
| El Salvador | 891 (354 to 1447) | 30 (12 to 48) | 33 (14 to 58) | 31 (13 to 52) | 25 (9 to 35) | -13.33 |
| Uruguay | 1073 (580 to 1503) | 36 (19 to 50) | 29 (16 to 43) | 37 (21 to 54) | 42 (20 to 53) | 18.06 |
| Venezuela, RB | 4139 (1956 to 6145) | 138 (65 to 205) | 113 (61 to 190) | 105 (49 to 155) | 196 (86 to 269) | 30.07 |
| **Europe** | 1449529 (1257213 to 1635786) | 48318 (41907 to 54526) | 47410 (42128 to 54815) | 46607 (40469 to 52673) | 50936 (43124 to 56076) | 3.65 |
| **Northern Europe** | 118797 (98327 to 136996) | 3960 (3278 to 4567) | 4260 (3570 to 4967) | 3826 (3134 to 4377) | 3794 (3128 to 4354) | -5.88 |
| Denmark | 7468 (6351 to 8549) | 249 (212 to 285) | 279 (245 to 328) | 251 (212 to 285) | 216 (178 to 242) | -12.65 |
| Estonia | 2546 (2085 to 2980) | 85 (70 to 99) | 100 (83 to 119) | 66 (55 to 79) | 88 (70 to 101) | -7.06 |
| Finland | 7304 (5987 to 8496) | 243 (200 to 283) | 248 (208 to 294) | 197 (164 to 232) | 286 (228 to 323) | 7.82 |
| United Kingdom | 67644 (56365 to 77925) | 2255 (1879 to 2598) | 2408 (2023 to 2795) | 2219 (1824 to 2529) | 2137 (1790 to 2468) | -6.01 |
| Ireland | 2671 (2085 to 3223) | 89 (70 to 107) | 95 (75 to 116) | 88 (67 to 103) | 84 (67 to 103) | -6.18 |
| Lithuania | 6979 (5838 to 8033) | 233 (195 to 268) | 242 (204 to 281) | 202 (172 to 236) | 254 (207 to 286) | 2.58 |
| Latvia | 5129 (4253 to 5944) | 171 (142 to 198) | 193 (161 to 224) | 145 (122 to 170) | 175 (143 to 200) | -5.26 |
| Norway | 5943 (4725 to 7181) | 198 (158 to 239) | 234 (186 to 279) | 217 (171 to 260) | 144 (115 to 180) | -22.73 |
| Sweden | 12909 (10639 to 14962) | 430 (355 to 499) | 452 (387 to 543) | 436 (353 to 496) | 403 (324 to 457) | -5.70 |
| **Southern Europe** | 305115 (268360 to 338070) | 10170 (8945 to 11269) | 9238 (8301 to 10449) | 10091 (8851 to 11157) | 11182 (9684 to 12201) | 9.56 |
| Albania | 4656 (4046 to 5191) | 155 (135 to 173) | 133 (116 to 149) | 148 (130 to 167) | 185 (158 to 203) | 16.77 |
| Bosnia and Herzegovina | 7148 (6233 to 7990) | 238 (208 to 266) | 210 (192 to 246) | 240 (207 to 266) | 265 (224 to 288) | 11.55 |
| Spain | 74136 (65371 to 82452) | 2471 (2179 to 2748) | 2261 (2047 to 2578) | 2405 (2122 to 2674) | 2748 (2368 to 2992) | 9.85 |
| Greece | 25020 (22293 to 27464) | 834 (743 to 915) | 690 (626 to 771) | 751 (670 to 826) | 1061 (933 to 1150) | 22.24 |
| Croatia | 11385 (10177 to 12568) | 380 (339 to 419) | 348 (324 to 400) | 408 (362 to 447) | 382 (332 to 410) | 4.47 |
| Italy | 127911 (112072 to 142841) | 4264 (3736 to 4761) | 3946 (3520 to 4481) | 4309 (3729 to 4752) | 4536 (3958 to 5051) | 6.92 |
| North Macedonia | 4508 (3949 to 5038) | 150 (132 to 168) | 135 (118 to 151) | 143 (131 to 167) | 173 (146 to 186) | 12.67 |
| Malta | 713 (625 to 789) | 24 (21 to 26) | 21 (19 to 24) | 24 (20 to 26) | 26 (23 to 29) | 10.42 |
| Montenegro | 1334 (1151 to 1502) | 44 (38 to 50) | 35 (30 to 40) | 46 (40 to 52) | 53 (45 to 59) | 20.45 |
| Portugal | 18183 (16273 to 19979) | 606 (542 to 666) | 593 (541 to 664) | 576 (520 to 639) | 650 (566 to 695) | 4.70 |
| Serbia | 26430 (22949 to 29605) | 881 (765 to 987) | 753 (669 to 863) | 922 (818 to 1054) | 967 (808 to 1044) | 12.15 |
| Slovenia | 3549 (3159 to 3935) | 118 (105 to 131) | 109 (100 to 124) | 116 (104 to 130) | 131 (112 to 140) | 9.32 |
| **Western Europe** | 284349 (247485 to 317982) | 9478 (8250 to 10599) | 9692 (8667 to 11124) | 9180 (7921 to 10182) | 9562 (8160 to 10492) | -0.69 |
| Austria | 13977 (11579 to 16391) | 466 (386 to 546) | 446 (387 to 548) | 432 (355 to 501) | 520 (416 to 590) | 7.94 |
| Belgium | 15151 (13391 to 16785) | 505 (446 to 560) | 528 (479 to 601) | 504 (441 to 553) | 482 (418 to 525) | -4.55 |
| Switzerland | 12996 (10048 to 15969) | 433 (335 to 532) | 439 (355 to 559) | 456 (341 to 544) | 405 (309 to 494) | -3.93 |
| Germany | 132415 (116656 to 147037) | 4414 (3889 to 4901) | 4615 (4178 to 5263) | 4177 (3645 to 4593) | 4449 (3842 to 4846) | -1.88 |
| France | 89490 (78580 to 99075) | 2983 (2619 to 3302) | 2968 (2662 to 3357) | 2941 (2569 to 3239) | 3040 (2626 to 3312) | 1.21 |
| Luxembourg | 634 (535 to 731) | 21 (18 to 24) | 23 (20 to 27) | 20 (17 to 23) | 20 (17 to 23) | -7.14 |
| Netherlands | 19547 (16981 to 21980) | 652 (566 to 733) | 669 (594 to 768) | 645 (564 to 730) | 641 (541 to 701) | -2.15 |
| **Eastern Europe** | 741268 (634590 to 843022) | 24709 (21153 to 28101) | 24220 (21311 to 28287) | 23509 (20295 to 26966) | 26398 (21854 to 29049) | 4.41 |
| Bulgaria | 26274 (23017 to 29504) | 876 (767 to 983) | 788 (718 to 916) | 897 (794 to 1020) | 943 (790 to 1015) | 8.85 |
| Belarus | 23198 (19738 to 26748) | 773 (658 to 892) | 779 (694 to 940) | 704 (598 to 811) | 837 (681 to 923) | 3.75 |
| Czech Republic | 17681 (15465 to 19696) | 589 (516 to 657) | 575 (524 to 668) | 559 (483 to 616) | 634 (539 to 686) | 5.01 |
| Hungary | 26902 (24008 to 29629) | 897 (800 to 988) | 852 (792 to 978) | 886 (786 to 970) | 952 (822 to 1015) | 5.57 |
| Moldova | 9008 (7872 to 10143) | 300 (262 to 338) | 309 (278 to 358) | 271 (236 to 304) | 321 (273 to 352) | 2.00 |
| Poland | 59088 (51399 to 66549) | 1970 (1713 to 2218) | 1931 (1746 to 2260) | 1795 (1571 to 2034) | 2183 (1822 to 2360) | 6.40 |
| Romania | 58405 (50853 to 65593) | 1947 (1695 to 2186) | 1803 (1620 to 2092) | 1889 (1665 to 2144) | 2149 (1800 to 2323) | 8.89 |
| Russian Federation | 362430 (305591 to 413817) | 12081 (10186 to 13794) | 11767 (10138 to 13705) | 11717 (9977 to 13519) | 12759 (10444 to 14158) | 4.11 |
| Slovak Republic | 9750 (8458 to 10985) | 325 (282 to 366) | 290 (261 to 339) | 315 (277 to 359) | 370 (308 to 400) | 12.31 |
| Ukraine | 148532 (127160 to 169168) | 4951 (4239 to 5639) | 5127 (4505 to 5992) | 4476 (3873 to 5151) | 5251 (4338 to 5774) | 1.25 |
| **Africa** | 634805 (363751 to 886714) | 21160 (12125 to 29557) | 22470 (12923 to 32162) | 19934 (11276 to 27704) | 21077 (12176 to 28806) | -3.29 |
| **Northern Africa** | 136159 (94667 to 173949) | 4539 (3156 to 5798) | 4187 (2997 to 5484) | 4224 (2902 to 5361) | 5205 (3567 to 6550) | 11.21 |
| Algeria | 18176 (11940 to 23792) | 606 (398 to 793) | 530 (370 to 735) | 625 (401 to 804) | 662 (423 to 840) | 10.89 |
| Egypt, Arab Rep. | 51960 (35398 to 66903) | 1732 (1180 to 2230) | 1606 (1128 to 2125) | 1513 (1038 to 1960) | 2077 (1374 to 2605) | 13.60 |
| Libya | 2408 (1692 to 3090) | 80 (56 to 103) | 72 (52 to 95) | 66 (45 to 82) | 103 (72 to 132) | 19.37 |
| Morocco | 16620 (9603 to 22918) | 554 (320 to 764) | 479 (303 to 718) | 583 (321 to 770) | 600 (336 to 806) | 10.92 |
| Sudan | 40521 (31236 to 50036) | 1351 (1041 to 1668) | 1328 (1012 to 1617) | 1209 (931 to 1493) | 1515 (1181 to 1893) | 6.92 |
| Tunisia | 6181 (4176 to 8078) | 206 (139 to 269) | 163 (115 to 222) | 216 (141 to 274) | 239 (161 to 311) | 18.45 |
| **Sub-Saharan Africa** | 498646 (269083 to 713473) | 16622 (8969 to 23782) | 18283 (9932 to 26694) | 15710 (8374 to 22376) | 15872 (8609 to 22277) | -7.25 |
| Angola | 8224 (4825 to 11456) | 274 (161 to 382) | 361 (218 to 526) | 222 (128 to 302) | 240 (137 to 318) | -22.08 |
| Burundi | 1169 (-368 to 2524) | 39 (-12 to 84) | 56 (-12 to 87) | 50 (-18 to 115) | 11 (-7 to 50) | -57.69 |
| Benin | 7961 (4364 to 11318) | 265 (145 to 377) | 266 (150 to 391) | 228 (122 to 314) | 302 (164 to 427) | 6.79 |
| Burkina Faso | 24789 (17213 to 32135) | 826 (574 to 1071) | 758 (554 to 1034) | 820 (545 to 1012) | 900 (623 to 1168) | 8.60 |
| Botswana | 1989 (1253 to 2693) | 66 (42 to 90) | 66 (42 to 91) | 75 (50 to 106) | 58 (34 to 72) | -6.06 |
| Central African Republic | 4079 (2152 to 5932) | 136 (72 to 198) | 139 (76 to 210) | 126 (66 to 182) | 142 (73 to 202) | 1.10 |
| Cote d'Ivoire | 14104 (7321 to 20506) | 470 (244 to 684) | 462 (250 to 699) | 495 (247 to 685) | 454 (236 to 666) | -0.85 |
| Cameroon | 12947 (7267 to 18232) | 432 (242 to 608) | 404 (233 to 601) | 422 (237 to 587) | 468 (256 to 635) | 7.41 |
| Congo, Dem. Rep. | 16546 (6676 to 26404) | 552 (223 to 880) | 639 (250 to 988) | 479 (200 to 783) | 537 (219 to 874) | -9.24 |
| Congo, Rep. | 1210 (487 to 1904) | 40 (16 to 63) | 43 (19 to 72) | 33 (13 to 51) | 45 (17 to 68) | 2.50 |
| Djibouti | 891 (691 to 1109) | 30 (23 to 37) | 30 (23 to 37) | 24 (18 to 29) | 35 (28 to 45) | 8.33 |
| Eritrea | 2358 (1236 to 3407) | 79 (41 to 114) | 68 (32 to 110) | 77 (41 to 106) | 90 (51 to 125) | 13.92 |
| Ethiopia | 24121 (7825 to 39987) | 804 (261 to 1333) | 997 (321 to 1667) | 742 (242 to 1267) | 674 (220 to 1065) | -20.09 |
| Gabon | 464 (172 to 731) | 15 (6 to 24) | 16 (6 to 26) | 17 (6 to 26) | 13 (5 to 21) | -10.00 |
| Ghana | 11124 (6158 to 15789) | 371 (205 to 526) | 401 (229 to 589) | 323 (175 to 448) | 389 (211 to 542) | -1.62 |
| Guinea | 8793 (5331 to 12115) | 293 (178 to 404) | 270 (174 to 394) | 334 (194 to 444) | 275 (165 to 374) | 0.85 |
| Gambia, The | 656 (435 to 850) | 22 (14 to 28) | 18 (13 to 26) | 17 (11 to 22) | 31 (19 to 37) | 29.55 |
| Guinea-Bissau | 1272 (817 to 1774) | 42 (27 to 59) | 46 (33 to 72) | 28 (18 to 38) | 54 (31 to 67) | 9.52 |
| Kenya | 10278 (573 to 19498) | 343 (19 to 650) | 288 (20 to 570) | 344 (17 to 677) | 395 (20 to 701) | 15.60 |
| Liberia | 1725 (966 to 2477) | 58 (32 to 83) | 87 (48 to 123) | 35 (20 to 51) | 51 (29 to 74) | -31.03 |
| Lesotho | 949 (90 to 1752) | 32 (3 to 58) | 22 (4 to 46) | 32 (3 to 64) | 41 (2 to 65) | 29.69 |
| Madagascar | 5844 (1672 to 10134) | 195 (56 to 338) | 218 (55 to 341) | 188 (61 to 356) | 179 (51 to 316) | -10.00 |
| Mali | 22729 (15784 to 28863) | 758 (526 to 962) | 742 (522 to 968) | 573 (409 to 743) | 958 (647 to 1175) | 14.25 |
| Mozambique | 15379 (7337 to 22868) | 513 (245 to 762) | 540 (261 to 821) | 563 (263 to 812) | 435 (210 to 653) | -10.23 |
| Mauritania | 1834 (1385 to 2249) | 61 (46 to 75) | 65 (50 to 81) | 56 (42 to 69) | 62 (46 to 75) | -2.46 |
| Mauritius | 461 (89 to 815) | 15 (3 to 27) | 14 (3 to 25) | 13 (3 to 25) | 20 (4 to 32) | 20.00 |
| Malawi | 10141 (4684 to 15677) | 338 (156 to 523) | 485 (221 to 722) | 350 (158 to 540) | 179 (90 to 307) | -45.27 |
| Namibia | 1435 (867 to 2013) | 48 (29 to 67) | 39 (26 to 60) | 54 (34 to 78) | 50 (27 to 63) | 11.46 |
| Niger | 31600 (23176 to 39373) | 1053 (773 to 1312) | 1051 (790 to 1338) | 1047 (740 to 1258) | 1062 (788 to 1341) | 0.52 |
| Nigeria | 137075 (82182 to 189200) | 4569 (2739 to 6307) | 5989 (3616 to 8382) | 4098 (2435 to 5518) | 3620 (2168 to 5020) | -25.92 |
| Rwanda | 1247 (-732 to 2801) | 42 (-24 to 93) | 99 (-43 to 162) | 18 (-18 to 69) | 8 (-13 to 50) | -108.33 |
| Senegal | 6986 (4748 to 9029) | 233 (158 to 301) | 245 (174 to 331) | 187 (124 to 238) | 267 (176 to 334) | 4.72 |
| Sierra Leone | 4885 (2971 to 6684) | 163 (99 to 223) | 159 (100 to 225) | 181 (107 to 239) | 148 (91 to 204) | -3.37 |
| Somalia | 10687 (7521 to 13756) | 356 (251 to 459) | 379 (270 to 486) | 323 (221 to 408) | 367 (261 to 479) | -1.69 |
| Eswatini | 678 (362 to 953) | 23 (12 to 32) | 15 (8 to 22) | 34 (18 to 47) | 19 (10 to 27) | 8.70 |
| Chad | 19642 (13971 to 24885) | 655 (466 to 830) | 607 (442 to 788) | 633 (443 to 790) | 724 (512 to 910) | 8.93 |
| Togo | 4137 (2228 to 5935) | 138 (74 to 198) | 138 (77 to 206) | 123 (64 to 170) | 153 (82 to 218) | 5.43 |
| Tanzania | 21252 (7829 to 33030) | 708 (261 to 1101) | 566 (232 to 982) | 688 (249 to 1073) | 871 (308 to 1248) | 21.54 |
| Uganda | 7787 (-1001 to 16635) | 260 (-33 to 554) | 306 (-44 to 608) | 200 (-28 to 488) | 272 (-32 to 566) | -6.54 |
| South Africa | 24592 (10649 to 37250) | 820 (355 to 1242) | 592 (275 to 965) | 933 (416 to 1456) | 935 (375 to 1304) | 20.91 |
| Zambia | 7517 (3645 to 11204) | 251 (122 to 373) | 351 (156 to 484) | 233 (122 to 374) | 168 (86 to 263) | -36.45 |
| Zimbabwe | 6455 (3076 to 9671) | 215 (103 to 322) | 222 (105 to 340) | 275 (130 to 405) | 149 (73 to 222) | -16.98 |
| **Asia** | 2248174 (1537831 to 2922561) | 74939 (51261 to 97419) | 71079 (49635 to 93650) | 71520 (49435 to 93588) | 82218 (54714 to 104981) | 7.43 |
| **Central Asia** | 37785 (24315 to 50238) | 1260 (810 to 1675) | 1091 (729 to 1510) | 1270 (829 to 1712) | 1418 (873 to 1802) | 12.98 |
| Kazakhstan | 11031 (6670 to 14972) | 368 (222 to 499) | 341 (218 to 487) | 356 (221 to 498) | 406 (228 to 513) | 8.83 |
| Kyrgyz Republic | 1948 (752 to 3086) | 65 (25 to 103) | 54 (24 to 96) | 69 (27 to 111) | 72 (24 to 102) | 13.85 |
| Tajikistan | 3477 (2048 to 4851) | 116 (68 to 162) | 116 (70 to 164) | 117 (68 to 159) | 115 (68 to 163) | -0.43 |
| Turkmenistan | 3604 (2457 to 4605) | 120 (82 to 154) | 98 (69 to 130) | 114 (79 to 148) | 148 (98 to 183) | 20.83 |
| Uzbekistan | 17726 (12238 to 22815) | 591 (408 to 760) | 482 (343 to 638) | 614 (430 to 800) | 677 (451 to 844) | 16.50 |
| **Southern Asia** | 1221925 (866061 to 1566217) | 40731 (28869 to 52207) | 41153 (29329 to 52728) | 39096 (27634 to 50217) | 41943 (29644 to 53694) | 0.97 |
| Afghanistan | 23274 (15823 to 29718) | 776 (527 to 991) | 583 (426 to 773) | 821 (552 to 1034) | 924 (604 to 1166) | 21.97 |
| Bangladesh | 41303 (26559 to 55123) | 1377 (885 to 1837) | 1483 (988 to 2026) | 1308 (832 to 1724) | 1339 (836 to 1763) | -5.23 |
| India | 952453 (676686 to 1220276) | 31748 (22556 to 40676) | 32887 (23486 to 42085) | 29834 (21186 to 38491) | 32525 (22996 to 41451) | -0.57 |
| Iran, Islamic Rep. | 31470 (22126 to 40426) | 1049 (738 to 1348) | 838 (592 to 1078) | 1014 (705 to 1288) | 1295 (915 to 1678) | 21.78 |
| Sri Lanka | 3939 (1777 to 5796) | 131 (59 to 193) | 112 (57 to 183) | 100 (44 to 144) | 181 (77 to 252) | 26.34 |
| Nepal | 8988 (4808 to 13069) | 300 (160 to 436) | 340 (203 to 486) | 238 (119 to 351) | 322 (159 to 470) | -3.00 |
| Pakistan | 160331 (120257 to 202265) | 5344 (4009 to 6742) | 4906 (3636 to 6126) | 5776 (4256 to 7160) | 5351 (4134 to 6940) | 4.16 |
| **Western Asia** | 94841 (64531 to 121916) | 3161 (2151 to 4064) | 2353 (1657 to 3174) | 2870 (1952 to 3663) | 4261 (2844 to 5384) | 30.18 |
| United Arab Emirates | 2468 (1686 to 3144) | 82 (56 to 105) | 41 (28 to 53) | 60 (43 to 80) | 147 (98 to 182) | 64.63 |
| Armenia | 1036 (304 to 1736) | 35 (10 to 58) | 25 (8 to 46) | 34 (9 to 52) | 44 (13 to 76) | 27.14 |
| Azerbaijan | 4595 (2601 to 6337) | 153 (87 to 211) | 99 (66 to 161) | 143 (80 to 195) | 218 (113 to 278) | 38.89 |
| Cyprus | 491 (301 to 668) | 16 (10 to 22) | 14 (9 to 20) | 17 (10 to 23) | 18 (11 to 24) | 12.50 |
| Georgia | 2321 (889 to 3683) | 77 (30 to 123) | 56 (25 to 102) | 80 (29 to 119) | 96 (35 to 148) | 25.97 |
| Iraq | 26255 (20404 to 32268) | 875 (680 to 1076) | 633 (500 to 792) | 912 (678 to 1073) | 1080 (862 to 1362) | 25.54 |
| Israel | 2069 (1310 to 2780) | 69 (44 to 93) | 56 (36 to 76) | 67 (43 to 90) | 84 (53 to 112) | 20.29 |
| Jordan | 1161 (674 to 1608) | 39 (22 to 54) | 32 (19 to 45) | 38 (23 to 54) | 46 (26 to 62) | 17.95 |
| Kuwait | 1134 (790 to 1451) | 38 (26 to 48) | 25 (19 to 35) | 29 (22 to 41) | 60 (38 to 70) | 46.05 |
| Lebanon | 985 (532 to 1380) | 33 (18 to 46) | 21 (13 to 34) | 32 (18 to 47) | 46 (22 to 57) | 37.88 |
| Oman | 1303 (927 to 1666) | 43 (31 to 56) | 42 (30 to 54) | 44 (32 to 57) | 44 (31 to 55) | 2.33 |
| West Bank and Gaza | 812 (491 to 1108) | 27 (16 to 37) | 22 (14 to 30) | 29 (18 to 40) | 30 (18 to 40) | 14.81 |
| Saudi Arabia | 13956 (10157 to 17480) | 465 (339 to 583) | 374 (285 to 489) | 428 (315 to 541) | 594 (416 to 718) | 23.66 |
| Syrian Arab Republic | 7434 (4979 to 9481) | 248 (166 to 316) | 161 (116 to 221) | 164 (108 to 206) | 419 (274 to 520) | 52.02 |
| Turkey | 18120 (10564 to 24767) | 604 (352 to 826) | 497 (321 to 744) | 527 (306 to 711) | 788 (429 to 1023) | 24.09 |
| Yemen, Rep. | 9895 (6135 to 13750) | 330 (204 to 458) | 237 (137 to 300) | 240 (162 to 364) | 513 (314 to 710) | 41.82 |
| **Eastern Asia** | 722389 (476757 to 938092) | 24080 (15892 to 31270) | 20794 (14322 to 28117) | 23447 (15810 to 30806) | 27998 (17544 to 34905) | 14.96 |
| China | 634740 (425405 to 823966) | 21158 (14180 to 27466) | 18488 (12845 to 24823) | 20950 (14297 to 27474) | 24037 (15399 to 30099) | 13.11 |
| Japan | 61012 (38503 to 81843) | 2034 (1283 to 2728) | 1509 (1067 to 2260) | 1738 (1121 to 2355) | 2854 (1662 to 3571) | 33.06 |
| Korea, Rep. | 15548 (8834 to 21794) | 518 (294 to 726) | 462 (279 to 691) | 440 (270 to 665) | 652 (335 to 823) | 18.34 |
| Mongolia | 479 (-324 to 1241) | 16 (-11 to 41) | 12 (-9 to 36) | 20 (-11 to 43) | 16 (-12 to 45) | 12.50 |
| Korea, Dem. People's Rep. | 10609 (4362 to 15981) | 354 (145 to 533) | 323 (141 to 514) | 299 (135 to 495) | 439 (159 to 589) | 16.38 |
| **South-eastern Asia** | 171234 (94238 to 242762) | 5708 (3141 to 8092) | 5688 (3230 to 8015) | 4837 (2780 to 7101) | 6599 (3409 to 9160) | 7.98 |
| Indonesia | 29996 (9908 to 48663) | 1000 (330 to 1622) | 764 (251 to 1275) | 903 (306 to 1422) | 1333 (436 to 2169) | 28.45 |
| Cambodia | 7298 (4414 to 10209) | 243 (147 to 340) | 294 (174 to 398) | 222 (137 to 318) | 214 (131 to 305) | -16.46 |
| Lao PDR | 3529 (2131 to 4869) | 118 (71 to 162) | 158 (96 to 217) | 94 (59 to 135) | 100 (58 to 135) | -24.58 |
| Myanmar | 32219 (20671 to 43423) | 1074 (689 to 1447) | 1461 (938 to 1954) | 815 (558 to 1193) | 946 (570 to 1195) | -23.98 |
| Malaysia | 4232 (1598 to 6576) | 141 (53 to 219) | 106 (42 to 171) | 107 (43 to 171) | 211 (75 to 316) | 37.23 |
| Philippines | 20907 (8858 to 32044) | 697 (295 to 1068) | 547 (233 to 846) | 577 (260 to 926) | 966 (393 to 1432) | 30.06 |
| Singapore | 735 (237 to 1203) | 24 (8 to 40) | 18 (6 to 30) | 27 (9 to 44) | 29 (9 to 46) | 22.92 |
| Thailand | 35655 (22175 to 47711) | 1188 (739 to 1590) | 1109 (725 to 1562) | 968 (642 to 1376) | 1488 (850 to 1833) | 15.95 |
| Vietnam | 36438 (23973 to 49578) | 1215 (799 to 1653) | 1224 (784 to 1602) | 1116 (759 to 1562) | 1304 (850 to 1793) | 3.29 |
| **Oceania** | 12996 (-2026 to 28000) | 433 (-68 to 933) | 417 (-58 to 918) | 406 (-58 to 858) | 476 (-82 to 1024) | 6.81 |
| **Australia and New Zealand** | 10273 (-1856 to 22473) | 342 (-62 to 749) | 308 (-58 to 712) | 332 (-52 to 718) | 388 (-75 to 818) | 11.70 |
| Australia | 9622 (-194 to 19458) | 321 (-6 to 649) | 287 (-3 to 613) | 313 (-5 to 631) | 362 (-12 to 702) | 11.68 |
| New Zealand | 652 (-1648 to 2995) | 22 (-55 to 100) | 20 (-55 to 98) | 19 (-47 to 86) | 26 (-63 to 115) | 13.64 |
| **Other regions in Oceania** | 2723 (236 to 5473) | 91 (8 to 182) | 110 (16 to 204) | 74 (4 to 139) | 89 (7 to 204) | -11.54 |
| Fiji | 489 (-98 to 1033) | 16 (-3 to 34) | 9 (-2 to 22) | 18 (-4 to 41) | 22 (-4 to 41) | 40.62 |
| Papua New Guinea | 1998 (357 to 3931) | 67 (12 to 131) | 94 (16 to 167) | 50 (8 to 86) | 56 (12 to 140) | -28.36 |

^a^ $\%Change per decade=\frac{Change per decade}{The mean value in 1990-2019}\times100\%$. Change per decade is calculated using a linear regression.
