# Supplementary material for: Global, regional, and national burden of heatwave-related mortality from 1990 to 2019: A three-stage modelling study
Source: PLoS Med. 2024 May 14;21(5):e1004364. doi: 10.1371/journal.pmed.1004364 (PMC11093289; doi:10.1371/journal.pmed.1004364)
Supplement: S7 Table — (DOCX) [file pmed.1004364.s016.docx]

**S7 Table.** Overall and average annual excess deaths (based on the age structure of WHO standard population) associated with heatwaves in warm seasons from 1990–1999 to 2010–2019 by continent, region, and countries. eCIs=empirical CIs. For country-specific data: To allow comparison, only countries in S6 Table were showed.

|  | **Overall** | **Average** | **1990-1999** | **2000-2009** | **2010–2019** | **%Change per decade ^a^** |
| --- | --- | --- | --- | --- | --- | --- |
| **Global** | 4684543 (3260066 to 6069215) | 156151 (108669 to 202307) | 161866 (114967 to 212177) | 150376 (104998 to 195185) | 156211 (106004 to 199559) | -1.81 |
| **Americas** | 217588 (102803 to 329202) | 7253 (3427 to 10973) | 7525 (3533 to 11446) | 6719 (3217 to 10255) | 7515 (3531 to 11220) | -0.07 |
| **Northern America** | 99457 (57543 to 140709) | 3315 (1918 to 4690) | 3470 (1990 to 4885) | 3010 (1757 to 4355) | 3466 (2007 to 4830) | -0.06 |
| Canada | 2588 (-620 to 5818) | 86 (-21 to 194) | 95 (-20 to 210) | 81 (-24 to 179) | 83 (-19 to 193) | -6.98 |
| United States | 96869 (58109 to 134901) | 3229 (1937 to 4497) | 3374 (2014 to 4676) | 2930 (1774 to 4176) | 3383 (2023 to 4638) | 0.14 |
| **Latin American and Caribbean** | 118131 (45601 to 189287) | 3938 (1520 to 6310) | 4056 (1536 to 6588) | 3708 (1478 to 5925) | 4049 (1546 to 6416) | -0.09 |
| Argentina | 8995 (4532 to 13189) | 300 (151 to 440) | 256 (139 to 402) | 303 (154 to 450) | 340 (160 to 467) | 14.00 |
| Bolivia | 1535 (-1274 to 4524) | 51 (-42 to 151) | 59 (-44 to 165) | 43 (-37 to 128) | 51 (-47 to 160) | -7.84 |
| Brazil | 43379 (19309 to 65683) | 1446 (644 to 2189) | 1430 (674 to 2290) | 1423 (598 to 2076) | 1485 (658 to 2204) | 1.90 |
| Colombia | 3361 (465 to 6145) | 112 (16 to 205) | 127 (24 to 237) | 91 (9 to 164) | 118 (13 to 213) | -4.02 |
| Costa Rica | 518 (142 to 890) | 17 (5 to 30) | 14 (3 to 24) | 14 (4 to 24) | 24 (7 to 41) | 29.41 |
| Cuba | 2332 (1049 to 3770) | 78 (35 to 126) | 96 (40 to 142) | 70 (32 to 113) | 67 (33 to 120) | -18.59 |
| Dominican Republic | 1420 (500 to 2383) | 47 (17 to 79) | 36 (11 to 54) | 57 (22 to 100) | 50 (16 to 84) | 14.89 |
| Ecuador | 974 (97 to 1866) | 32 (3 to 62) | 32 (3 to 58) | 23 (4 to 46) | 43 (3 to 82) | 17.19 |
| Guatemala | 1722 (81 to 3255) | 57 (3 to 108) | 70 (4 to 136) | 49 (2 to 90) | 54 (2 to 100) | -14.04 |
| Honduras | 1383 (541 to 2234) | 46 (18 to 74) | 45 (18 to 72) | 43 (16 to 68) | 50 (20 to 84) | 5.43 |
| Haiti | 2895 (1424 to 4565) | 96 (47 to 152) | 76 (35 to 110) | 138 (68 to 223) | 76 (37 to 122) | 0.00 |
| Jamaica | 594 (205 to 963) | 20 (7 to 32) | 18 (6 to 30) | 21 (7 to 34) | 20 (7 to 33) | 5.00 |
| Mexico | 33678 (17926 to 49432) | 1123 (598 to 1648) | 1268 (635 to 1766) | 988 (576 to 1519) | 1112 (582 to 1658) | -6.95 |
| Nicaragua | 1607 (938 to 2400) | 54 (31 to 80) | 48 (26 to 66) | 56 (32 to 80) | 57 (36 to 93) | 8.33 |
| Panama | 533 (236 to 814) | 18 (8 to 27) | 16 (7 to 25) | 14 (6 to 22) | 23 (10 to 34) | 19.44 |
| Peru | 3098 (-2927 to 9346) | 103 (-98 to 312) | 145 (-118 to 394) | 78 (-75 to 255) | 87 (-99 to 286) | -28.16 |
| Paraguay | 1894 (1285 to 2423) | 63 (43 to 81) | 49 (35 to 66) | 60 (42 to 80) | 80 (51 to 96) | 24.60 |
| El Salvador | 1044 (418 to 1708) | 35 (14 to 57) | 41 (18 to 73) | 37 (15 to 62) | 26 (9 to 36) | -21.43 |
| Uruguay | 741 (402 to 1044) | 25 (13 to 35) | 22 (13 to 33) | 26 (15 to 38) | 26 (13 to 34) | 8.00 |
| Venezuela, RB | 5173 (2471 to 7761) | 172 (82 to 259) | 166 (89 to 278) | 137 (65 to 204) | 214 (94 to 294) | 13.95 |
| **Europe** | 914990 (794083 to 1035093) | 30500 (26469 to 34503) | 34098 (30280 to 39416) | 29130 (25296 to 32992) | 28271 (23832 to 31101) | -9.55 |
| **Northern Europe** | 67158 (55593 to 77500) | 2239 (1853 to 2583) | 2590 (2170 to 3020) | 2166 (1774 to 2479) | 1960 (1615 to 2251) | -14.07 |
| Denmark | 4211 (3586 to 4826) | 140 (120 to 161) | 166 (146 to 195) | 144 (122 to 164) | 110 (91 to 123) | -20.00 |
| Estonia | 1608 (1319 to 1886) | 54 (44 to 63) | 75 (62 to 88) | 41 (34 to 49) | 45 (36 to 52) | -27.78 |
| Finland | 4048 (3328 to 4720) | 135 (111 to 157) | 160 (134 to 191) | 110 (91 to 129) | 135 (107 to 152) | -9.26 |
| United Kingdom | 37746 (31454 to 43486) | 1258 (1048 to 1450) | 1410 (1184 to 1636) | 1244 (1022 to 1418) | 1121 (939 to 1295) | -11.49 |
| Ireland | 2000 (1561 to 2414) | 67 (52 to 80) | 75 (59 to 92) | 67 (51 to 79) | 58 (46 to 71) | -12.69 |
| Lithuania | 4577 (3839 to 5281) | 153 (128 to 176) | 192 (162 to 224) | 132 (113 to 155) | 133 (109 to 149) | -19.28 |
| Latvia | 3215 (2668 to 3730) | 107 (89 to 124) | 142 (118 to 165) | 90 (76 to 106) | 89 (73 to 102) | -24.77 |
| Norway | 3329 (2647 to 4021) | 111 (88 to 134) | 132 (105 to 158) | 122 (96 to 145) | 79 (63 to 99) | -23.87 |
| Sweden | 6279 (5180 to 7284) | 209 (173 to 243) | 230 (197 to 276) | 212 (172 to 242) | 185 (149 to 210) | -10.77 |
| **Southern Europe** | 166625 (146908 to 185240) | 5554 (4897 to 6175) | 6030 (5424 to 6830) | 5458 (4799 to 6050) | 5174 (4472 to 5644) | -7.71 |
| Albania | 4454 (3882 to 4980) | 148 (129 to 166) | 170 (149 to 191) | 145 (129 to 165) | 130 (110 to 142) | -13.51 |
| Bosnia and Herzegovina | 5516 (4840 to 6204) | 184 (161 to 207) | 212 (193 to 247) | 183 (159 to 203) | 157 (133 to 170) | -14.95 |
| Spain | 40406 (35711 to 45035) | 1347 (1190 to 1501) | 1427 (1292 to 1627) | 1296 (1144 to 1441) | 1318 (1136 to 1436) | -4.05 |
| Greece | 12914 (11530 to 14203) | 430 (384 to 473) | 435 (395 to 486) | 397 (354 to 437) | 460 (404 to 498) | 2.91 |
| Croatia | 6746 (6057 to 7481) | 225 (202 to 249) | 254 (236 to 291) | 236 (209 to 258) | 185 (161 to 199) | -15.33 |
| Italy | 61508 (53961 to 68766) | 2050 (1799 to 2292) | 2247 (2004 to 2552) | 2046 (1771 to 2257) | 1858 (1621 to 2068) | -9.49 |
| North Macedonia | 3803 (3338 to 4259) | 127 (111 to 142) | 138 (121 to 155) | 119 (108 to 138) | 123 (104 to 133) | -5.91 |
| Malta | 450 (395 to 499) | 15 (13 to 17) | 17 (15 to 19) | 15 (13 to 16) | 13 (12 to 15) | -13.33 |
| Montenegro | 1013 (874 to 1141) | 34 (29 to 38) | 33 (28 to 37) | 35 (30 to 39) | 34 (29 to 38) | 1.47 |
| Portugal | 10055 (9026 to 11080) | 335 (301 to 369) | 394 (360 to 441) | 318 (288 to 353) | 293 (256 to 314) | -15.07 |
| Serbia | 17503 (15252 to 19676) | 583 (508 to 656) | 621 (551 to 711) | 595 (528 to 680) | 535 (447 to 577) | -7.38 |
| Slovenia | 2167 (1938 to 2413) | 72 (65 to 80) | 82 (75 to 94) | 70 (63 to 79) | 65 (56 to 69) | -11.81 |
| **Western Europe** | 153331 (133713 to 171866) | 5111 (4457 to 5729) | 5955 (5324 to 6833) | 4944 (4266 to 5486) | 4434 (3782 to 4868) | -14.88 |
| Austria | 7601 (6316 to 8942) | 253 (211 to 298) | 271 (235 to 332) | 240 (197 to 278) | 250 (200 to 284) | -4.15 |
| Belgium | 8237 (7293 to 9141) | 275 (243 to 305) | 319 (289 to 362) | 268 (234 to 293) | 237 (206 to 258) | -14.91 |
| Switzerland | 7104 (5505 to 8742) | 237 (184 to 291) | 264 (214 to 336) | 249 (186 to 297) | 198 (151 to 241) | -13.92 |
| Germany | 67354 (59492 to 74978) | 2245 (1983 to 2499) | 2716 (2459 to 3097) | 2135 (1863 to 2348) | 1885 (1628 to 2053) | -18.51 |
| France | 50818 (44695 to 56354) | 1694 (1490 to 1878) | 1917 (1719 to 2168) | 1646 (1438 to 1812) | 1519 (1312 to 1655) | -11.75 |
| Luxembourg | 403 (341 to 464) | 13 (11 to 15) | 15 (13 to 18) | 13 (11 to 14) | 12 (10 to 14) | -11.54 |
| Netherlands | 11746 (10226 to 13235) | 392 (341 to 441) | 452 (400 to 518) | 392 (343 to 444) | 331 (279 to 362) | -15.43 |
| **Eastern Europe** | 527876 (452540 to 602050) | 17596 (15085 to 20068) | 19523 (17162 to 22802) | 16562 (14282 to 19007) | 16703 (13810 to 18396) | -8.01 |
| Bulgaria | 14961 (13160 to 16861) | 499 (439 to 562) | 526 (479 to 612) | 502 (445 to 571) | 468 (392 to 504) | -5.81 |
| Belarus | 16428 (14031 to 19017) | 548 (468 to 634) | 626 (558 to 756) | 492 (419 to 568) | 525 (427 to 579) | -9.22 |
| Czech Republic | 10849 (9522 to 12128) | 362 (317 to 404) | 409 (373 to 475) | 339 (293 to 373) | 337 (286 to 365) | -9.94 |
| Hungary | 16329 (14623 to 18047) | 544 (487 to 602) | 596 (554 to 684) | 535 (474 to 586) | 502 (434 to 535) | -8.64 |
| Moldova | 7492 (6570 to 8464) | 250 (219 to 282) | 302 (271 to 350) | 223 (194 to 250) | 225 (191 to 246) | -15.40 |
| Poland | 41604 (36365 to 47082) | 1387 (1212 to 1569) | 1629 (1474 to 1907) | 1236 (1082 to 1400) | 1295 (1081 to 1401) | -12.04 |
| Romania | 39178 (34265 to 44201) | 1306 (1142 to 1473) | 1471 (1322 to 1707) | 1256 (1107 to 1426) | 1191 (998 to 1288) | -10.72 |
| Russian Federation | 273617 (231117 to 312938) | 9121 (7704 to 10431) | 9917 (8544 to 11550) | 8750 (7451 to 10096) | 8695 (7117 to 9648) | -6.70 |
| Slovak Republic | 7098 (6180 to 8026) | 237 (206 to 268) | 245 (220 to 286) | 229 (202 to 261) | 236 (196 to 255) | -1.90 |
| Ukraine | 100324 (86058 to 114486) | 3344 (2869 to 3816) | 3804 (3342 to 4446) | 2999 (2595 to 3451) | 3230 (2668 to 3551) | -8.58 |
| **Africa** | 795691 (452206 to 1115541) | 26523 (15074 to 37185) | 25868 (14772 to 37180) | 24934 (13997 to 34847) | 28767 (16452 to 39526) | 5.47 |
| **Northern Africa** | 190958 (132825 to 244019) | 6365 (4428 to 8134) | 5578 (3986 to 7347) | 5986 (4114 to 7603) | 7533 (5182 to 9472) | 15.36 |
| Algeria | 25189 (16577 to 33037) | 840 (553 to 1101) | 787 (549 to 1091) | 894 (573 to 1150) | 838 (536 to 1063) | 3.04 |
| Egypt, Arab Rep. | 79131 (53870 to 101826) | 2638 (1796 to 3394) | 2321 (1630 to 3071) | 2362 (1620 to 3060) | 3230 (2136 to 4052) | 17.23 |
| Libya | 3600 (2531 to 4622) | 120 (84 to 154) | 112 (81 to 149) | 107 (73 to 134) | 141 (99 to 180) | 12.08 |
| Morocco | 21387 (12377 to 29534) | 713 (413 to 984) | 645 (408 to 966) | 758 (418 to 1001) | 735 (412 to 988) | 6.31 |
| Sudan | 54141 (41788 to 66949) | 1805 (1393 to 2232) | 1487 (1133 to 1811) | 1592 (1226 to 1966) | 2335 (1820 to 2918) | 23.49 |
| Tunisia | 7129 (4825 to 9333) | 238 (161 to 311) | 215 (152 to 294) | 257 (168 to 327) | 241 (162 to 313) | 5.46 |
| **Sub-Saharan Africa** | 604733 (319381 to 872353) | 20158 (10646 to 29078) | 20290 (10786 to 29869) | 18949 (9883 to 27288) | 21234 (11270 to 30077) | 2.34 |
| Angola | 10546 (6170 to 14622) | 352 (206 to 487) | 412 (248 to 602) | 280 (162 to 382) | 363 (206 to 480) | -6.96 |
| Burundi | 1521 (-491 to 3362) | 51 (-16 to 112) | 69 (-14 to 108) | 67 (-24 to 153) | 16 (-11 to 76) | -51.96 |
| Benin | 9837 (5385 to 13968) | 328 (180 to 466) | 298 (168 to 437) | 283 (151 to 390) | 403 (219 to 570) | 16.01 |
| Burkina Faso | 28617 (19844 to 37051) | 954 (661 to 1235) | 797 (582 to 1087) | 937 (622 to 1155) | 1128 (780 to 1463) | 17.35 |
| Botswana | 2833 (1784 to 3836) | 94 (59 to 128) | 97 (62 to 134) | 104 (69 to 148) | 82 (48 to 102) | -7.98 |
| Central African Republic | 5401 (2848 to 7847) | 180 (95 to 262) | 176 (96 to 265) | 168 (88 to 242) | 196 (100 to 278) | 5.56 |
| Cote d'Ivoire | 19067 (9891 to 27711) | 636 (330 to 924) | 591 (320 to 894) | 662 (330 to 916) | 654 (340 to 961) | 4.95 |
| Cameroon | 17312 (9705 to 24322) | 577 (324 to 811) | 499 (287 to 742) | 555 (312 to 770) | 678 (372 to 920) | 15.51 |
| Congo, Dem. Rep. | 21331 (8617 to 34101) | 711 (287 to 1137) | 737 (288 to 1139) | 601 (252 to 984) | 795 (324 to 1294) | 4.08 |
| Congo, Rep. | 1764 (709 to 2772) | 59 (24 to 92) | 60 (26 to 100) | 48 (18 to 72) | 69 (27 to 105) | 7.63 |
| Djibouti | 1262 (981 to 1574) | 42 (33 to 52) | 42 (31 to 50) | 33 (25 to 41) | 51 (41 to 66) | 10.71 |
| Eritrea | 3792 (2001 to 5454) | 126 (67 to 182) | 98 (45 to 158) | 126 (67 to 172) | 156 (88 to 215) | 23.02 |
| Ethiopia | 32777 (10639 to 54229) | 1093 (355 to 1808) | 1245 (401 to 2082) | 1002 (327 to 1712) | 1030 (336 to 1629) | -9.84 |
| Gabon | 640 (237 to 1009) | 21 (8 to 34) | 21 (8 to 34) | 24 (8 to 36) | 19 (7 to 31) | -4.76 |
| Ghana | 15407 (8521 to 21849) | 514 (284 to 728) | 528 (302 to 776) | 444 (241 to 616) | 569 (309 to 793) | 3.99 |
| Guinea | 9707 (5867 to 13334) | 324 (196 to 444) | 258 (166 to 377) | 363 (211 to 481) | 350 (210 to 475) | 14.20 |
| Gambia, The | 960 (634 to 1237) | 32 (21 to 41) | 24 (17 to 33) | 24 (16 to 32) | 48 (30 to 59) | 37.50 |
| Guinea-Bissau | 1762 (1119 to 2428) | 59 (37 to 81) | 55 (39 to 86) | 37 (24 to 52) | 84 (48 to 105) | 24.58 |
| Kenya | 15683 (868 to 29660) | 523 (29 to 989) | 403 (28 to 797) | 518 (26 to 1018) | 648 (33 to 1148) | 23.42 |
| Liberia | 2070 (1162 to 2979) | 69 (39 to 99) | 87 (48 to 124) | 43 (24 to 63) | 77 (43 to 112) | -7.25 |
| Lesotho | 1224 (115 to 2259) | 41 (4 to 75) | 29 (5 to 61) | 41 (4 to 82) | 52 (2 to 84) | 28.05 |
| Madagascar | 8364 (2408 to 14587) | 279 (80 to 486) | 274 (69 to 430) | 274 (89 to 521) | 288 (82 to 508) | 2.51 |
| Mali | 24046 (16670 to 30460) | 802 (556 to 1015) | 705 (496 to 920) | 590 (421 to 765) | 1110 (750 to 1361) | 25.25 |
| Mozambique | 19223 (9170 to 28570) | 641 (306 to 952) | 584 (282 to 888) | 700 (327 to 1011) | 638 (308 to 958) | 4.21 |
| Mauritania | 2455 (1852 to 3008) | 82 (62 to 100) | 81 (63 to 101) | 75 (56 to 92) | 90 (67 to 108) | 5.49 |
| Mauritius | 489 (95 to 872) | 16 (3 to 29) | 18 (4 to 32) | 14 (3 to 27) | 17 (3 to 28) | -3.13 |
| Malawi | 12772 (5924 to 19867) | 426 (197 to 662) | 548 (249 to 815) | 454 (204 to 698) | 276 (139 to 474) | -31.92 |
| Namibia | 2001 (1208 to 2807) | 67 (40 to 94) | 55 (37 to 85) | 75 (47 to 109) | 70 (38 to 88) | 11.19 |
| Niger | 32306 (23690 to 40255) | 1077 (790 to 1342) | 937 (704 to 1193) | 1049 (741 to 1259) | 1245 (924 to 1573) | 14.30 |
| Nigeria | 151716 (90934 to 209343) | 5057 (3031 to 6978) | 6116 (3692 to 8559) | 4536 (2695 to 6108) | 4520 (2706 to 6267) | -15.78 |
| Rwanda | 1603 (-979 to 3750) | 53 (-33 to 125) | 122 (-53 to 200) | 25 (-25 to 96) | 13 (-20 to 78) | -102.83 |
| Senegal | 9273 (6288 to 11957) | 309 (210 to 399) | 297 (211 to 400) | 245 (163 to 313) | 385 (254 to 482) | 14.24 |
| Sierra Leone | 5475 (3329 to 7487) | 182 (111 to 250) | 156 (98 to 221) | 201 (119 to 266) | 190 (116 to 262) | 9.34 |
| Somalia | 14431 (10158 to 18586) | 481 (339 to 620) | 482 (343 to 618) | 428 (294 to 542) | 533 (379 to 696) | 5.30 |
| Eswatini | 992 (532 to 1395) | 33 (18 to 46) | 24 (13 to 34) | 49 (26 to 67) | 27 (14 to 38) | 4.55 |
| Chad | 21880 (15550 to 27692) | 729 (518 to 923) | 606 (442 to 787) | 698 (489 to 871) | 884 (625 to 1111) | 19.07 |
| Togo | 5688 (3062 to 8156) | 190 (102 to 272) | 179 (99 to 266) | 168 (88 to 233) | 222 (119 to 317) | 11.32 |
| Tanzania | 27821 (10211 to 42994) | 927 (340 to 1433) | 666 (272 to 1155) | 886 (320 to 1382) | 1231 (435 to 1763) | 30.47 |
| Uganda | 10554 (-1352 to 22590) | 352 (-45 to 753) | 367 (-53 to 729) | 272 (-38 to 664) | 416 (-50 to 864) | 6.96 |
| South Africa | 29420 (12764 to 44652) | 981 (425 to 1488) | 749 (348 to 1222) | 1111 (495 to 1735) | 1082 (433 to 1508) | 16.97 |
| Zambia | 10498 (5117 to 15724) | 350 (171 to 524) | 449 (200 to 619) | 329 (173 to 528) | 272 (139 to 426) | -25.29 |
| Zimbabwe | 9399 (4481 to 14081) | 313 (149 to 469) | 323 (153 to 493) | 389 (183 to 573) | 228 (112 to 342) | -15.18 |
| **Asia** | 2745290 (1891436 to 3576010) | 91510 (63048 to 119200) | 93976 (65642 to 123560) | 89258 (61915 to 116768) | 91295 (61586 to 117224) | -1.46 |
| **Central Asia** | 55482 (36019 to 73537) | 1849 (1201 to 2451) | 1519 (1021 to 2094) | 1910 (1258 to 2562) | 2119 (1326 to 2698) | 16.22 |
| Kazakhstan | 12412 (7519 to 16877) | 414 (251 to 563) | 407 (260 to 581) | 396 (246 to 554) | 438 (246 to 553) | 3.74 |
| Kyrgyz Republic | 2619 (1009 to 4144) | 87 (34 to 138) | 70 (31 to 124) | 93 (37 to 150) | 99 (34 to 140) | 16.67 |
| Tajikistan | 5553 (3268 to 7745) | 185 (109 to 258) | 156 (94 to 220) | 191 (110 to 260) | 208 (123 to 295) | 14.05 |
| Turkmenistan | 5005 (3421 to 6408) | 167 (114 to 214) | 143 (102 to 191) | 166 (114 to 214) | 192 (126 to 237) | 14.67 |
| Uzbekistan | 29892 (20618 to 38439) | 996 (687 to 1281) | 742 (528 to 982) | 1065 (746 to 1388) | 1182 (788 to 1474) | 22.09 |
| **Southern Asia** | 1622114 (1150051 to 2079521) | 54070 (38335 to 69317) | 55989 (39891 to 71747) | 52908 (37387 to 67957) | 53314 (37727 to 68286) | -2.47 |
| Afghanistan | 34254 (23136 to 43618) | 1142 (771 to 1454) | 672 (492 to 891) | 1169 (786 to 1472) | 1585 (1036 to 2000) | 39.97 |
| Bangladesh | 55991 (36018 to 74726) | 1866 (1201 to 2491) | 2008 (1337 to 2743) | 1901 (1209 to 2504) | 1691 (1056 to 2226) | -8.49 |
| India | 1254630 (891541 to 1607644) | 41821 (29718 to 53588) | 45396 (32420 to 58093) | 40010 (28412 to 51621) | 40057 (28322 to 51050) | -6.38 |
| Iran, Islamic Rep. | 42432 (29828 to 54481) | 1414 (994 to 1816) | 1254 (887 to 1614) | 1413 (983 to 1795) | 1576 (1113 to 2043) | 11.39 |
| Sri Lanka | 4407 (2009 to 6541) | 147 (67 to 218) | 148 (75 to 242) | 116 (51 to 166) | 177 (75 to 246) | 9.86 |
| Nepal | 11813 (6300 to 17186) | 394 (210 to 573) | 425 (254 to 608) | 326 (163 to 482) | 430 (213 to 629) | 0.63 |
| Pakistan | 218344 (163923 to 275682) | 7278 (5464 to 9189) | 6079 (4506 to 7592) | 7965 (5869 to 9874) | 7790 (6018 to 10103) | 11.75 |
| **Western Asia** | 146378 (102796 to 186613) | 4879 (3427 to 6220) | 3650 (2614 to 4871) | 4556 (3193 to 5754) | 6432 (4417 to 8081) | 28.51 |
| United Arab Emirates | 7520 (5153 to 9614) | 251 (172 to 320) | 129 (90 to 168) | 217 (156 to 290) | 406 (270 to 504) | 55.18 |
| Armenia | 930 (276 to 1575) | 31 (9 to 52) | 30 (10 to 56) | 30 (8 to 46) | 33 (10 to 56) | 4.84 |
| Azerbaijan | 5877 (3338 to 8129) | 196 (111 to 271) | 131 (88 to 214) | 190 (107 to 260) | 266 (138 to 339) | 34.44 |
| Cyprus | 381 (236 to 519) | 13 (8 to 17) | 12 (8 to 17) | 14 (8 to 18) | 12 (8 to 16) | 0.00 |
| Georgia | 1637 (634 to 2620) | 55 (21 to 87) | 49 (22 to 88) | 56 (20 to 83) | 59 (21 to 91) | 9.09 |
| Iraq | 42291 (32855 to 51959) | 1410 (1095 to 1732) | 966 (763 to 1208) | 1494 (1111 to 1757) | 1769 (1412 to 2230) | 28.48 |
| Israel | 1744 (1105 to 2344) | 58 (37 to 78) | 51 (33 to 69) | 56 (36 to 76) | 67 (42 to 89) | 13.79 |
| Jordan | 2178 (1265 to 3021) | 73 (42 to 101) | 63 (38 to 90) | 74 (44 to 104) | 81 (45 to 108) | 12.33 |
| Kuwait | 2082 (1449 to 2664) | 69 (48 to 89) | 48 (37 to 68) | 50 (39 to 71) | 110 (69 to 127) | 44.93 |
| Lebanon | 999 (545 to 1413) | 33 (18 to 47) | 26 (16 to 41) | 31 (18 to 46) | 43 (21 to 54) | 25.76 |
| Oman | 2978 (2116 to 3805) | 99 (71 to 127) | 93 (66 to 119) | 97 (70 to 126) | 108 (76 to 135) | 7.58 |
| West Bank and Gaza | 1484 (896 to 2023) | 49 (30 to 67) | 38 (24 to 53) | 51 (31 to 70) | 59 (35 to 79) | 21.43 |
| Saudi Arabia | 27075 (19704 to 33911) | 902 (657 to 1130) | 715 (545 to 936) | 843 (621 to 1066) | 1149 (804 to 1388) | 24.06 |
| Syrian Arab Republic | 11542 (7761 to 14780) | 385 (259 to 493) | 288 (208 to 396) | 317 (210 to 399) | 549 (359 to 683) | 33.90 |
| Turkey | 20656 (12138 to 28421) | 689 (405 to 947) | 659 (425 to 985) | 614 (357 to 829) | 793 (432 to 1030) | 9.72 |
| Yemen, Rep. | 14783 (9176 to 20586) | 493 (306 to 686) | 302 (175 to 384) | 345 (234 to 524) | 832 (509 to 1151) | 53.75 |
| **Eastern Asia** | 705389 (469468 to 922382) | 23513 (15649 to 30746) | 25122 (17291 to 33913) | 23638 (15957 to 31126) | 21779 (13699 to 27198) | -7.11 |
| China | 651086 (438876 to 849328) | 21703 (14629 to 28311) | 23187 (16110 to 31132) | 22039 (15040 to 28903) | 19883 (12738 to 24898) | -7.61 |
| Japan | 28420 (18275 to 38794) | 947 (609 to 1293) | 970 (686 to 1453) | 822 (530 to 1114) | 1049 (611 to 1313) | 4.17 |
| Korea, Rep. | 14445 (8362 to 20645) | 482 (279 to 688) | 586 (354 to 877) | 421 (258 to 636) | 437 (224 to 552) | -15.46 |
| Mongolia | 747 (-503 to 1920) | 25 (-17 to 64) | 19 (-14 to 56) | 33 (-19 to 72) | 23 (-17 to 64) | 8.00 |
| Korea, Dem. People's Rep. | 10692 (4439 to 16260) | 356 (148 to 542) | 359 (157 to 572) | 323 (146 to 536) | 387 (140 to 518) | 3.93 |
| **South-eastern Asia** | 215927 (117566 to 308956) | 7198 (3919 to 10299) | 7696 (4327 to 10886) | 6246 (3535 to 9222) | 7651 (3876 to 10788) | -0.31 |
| Indonesia | 40660 (13436 to 65972) | 1355 (448 to 2199) | 1091 (358 to 1821) | 1251 (423 to 1970) | 1724 (565 to 2806) | 23.36 |
| Cambodia | 10032 (6071 to 14041) | 334 (202 to 468) | 395 (234 to 536) | 317 (196 to 453) | 291 (178 to 414) | -15.57 |
| Lao PDR | 4719 (2847 to 6507) | 157 (95 to 217) | 194 (117 to 267) | 131 (82 to 188) | 146 (85 to 196) | -15.29 |
| Myanmar | 39822 (25563 to 53699) | 1327 (852 to 1790) | 1830 (1175 to 2448) | 1014 (693 to 1484) | 1139 (686 to 1438) | -26.04 |
| Malaysia | 6194 (2353 to 9672) | 206 (78 to 322) | 181 (71 to 293) | 170 (68 to 272) | 269 (96 to 403) | 21.36 |
| Philippines | 30160 (12798 to 46273) | 1005 (427 to 1542) | 811 (346 to 1254) | 862 (388 to 1382) | 1344 (546 to 1992) | 26.52 |
| Singapore | 782 (253 to 1284) | 26 (8 to 43) | 23 (8 to 39) | 30 (10 to 49) | 25 (8 to 40) | 3.85 |
| Thailand | 36717 (23096 to 49684) | 1224 (770 to 1656) | 1424 (931 to 2006) | 1025 (681 to 1457) | 1222 (698 to 1505) | -8.25 |
| Vietnam | 46516 (30591 to 63175) | 1551 (1020 to 2106) | 1737 (1113 to 2274) | 1436 (976 to 2009) | 1479 (964 to 2034) | -8.32 |
| **Oceania** | 10984 (-1245 to 23526) | 366 (-42 to 784) | 400 (-34 to 848) | 334 (-44 to 696) | 364 (-46 to 800) | -4.92 |
| **Australia and New Zealand** | 6886 (-1282 to 15166) | 230 (-43 to 506) | 236 (-46 to 549) | 221 (-37 to 480) | 231 (-46 to 488) | -1.09 |
| Australia | 6435 (-124 to 13067) | 214 (-4 to 436) | 220 (-2 to 470) | 208 (-3 to 418) | 216 (-7 to 418) | -0.93 |
| New Zealand | 451 (-1147 to 2083) | 15 (-38 to 69) | 16 (-43 to 78) | 13 (-33 to 61) | 16 (-38 to 70) | 0.00 |
| **Other regions in Oceania** | 4098 (345 to 8281) | 137 (12 to 276) | 163 (21 to 305) | 113 (6 to 214) | 133 (10 to 309) | -10.95 |
| Fiji | 712 (-143 to 1517) | 24 (-5 to 51) | 16 (-3 to 37) | 27 (-6 to 62) | 28 (-6 to 53) | 25.00 |
| Papua New Guinea | 2986 (536 to 5899) | 100 (18 to 197) | 136 (23 to 241) | 77 (12 to 132) | 86 (19 to 217) | -25.00 |

^a^ $\%Change per decade=\frac{Change per decade}{The mean value in 1990-2019}\times100\%$. Change per decade is calculated using a linear regression.
