# Supplementary material for: Global, regional, and national burden of heatwave-related mortality from 1990 to 2019: A three-stage modelling study
Source: PLoS Med. 2024 May 14;21(5):e1004364. doi: 10.1371/journal.pmed.1004364 (PMC11093289; doi:10.1371/journal.pmed.1004364)
Supplement: S9 Table — (DOCX) [file pmed.1004364.s018.docx]

**S9 Table.** Average excess death ratio (%, based on the age structure of WHO standard population) associated with heatwaves per warm season from 1990–1999 to 2010–2019 by continent, region and countries. eCIs=empirical CIs. For country-specific data: To allow comparison, only countries in S6 Table were showed.

|  | **Average** | **1990-1999** | **2000-2009** | **2010–2019** | **%Change per decade ^a^** |
| --- | --- | --- | --- | --- | --- |
| **Global** | 0.87 (0.61 to 1.13) | 0.90 (0.64 to 1.18) | 0.82 (0.57 to 1.07) | 0.90 (0.61 to 1.15) | 0.00 |
| **Americas** | 0.41 (0.19 to 0.62) | 0.43 (0.20 to 0.65) | 0.38 (0.18 to 0.58) | 0.42 (0.20 to 0.62) | -1.22 |
| **Northern America** | 0.58 (0.33 to 0.82) | 0.61 (0.35 to 0.86) | 0.52 (0.30 to 0.76) | 0.60 (0.35 to 0.84) | -0.86 |
| Canada | 0.19 (-0.04 to 0.42) | 0.20 (-0.04 to 0.44) | 0.18 (-0.05 to 0.39) | 0.18 (-0.04 to 0.43) | -5.26 |
| United States | 0.61 (0.37 to 0.85) | 0.65 (0.39 to 0.90) | 0.55 (0.33 to 0.79) | 0.64 (0.38 to 0.88) | -0.82 |
| **Latin American and Caribbean** | 0.33 (0.13 to 0.53) | 0.34 (0.13 to 0.56) | 0.31 (0.12 to 0.50) | 0.33 (0.13 to 0.52) | -1.52 |
| Argentina | 0.38 (0.19 to 0.55) | 0.33 (0.18 to 0.52) | 0.38 (0.19 to 0.57) | 0.41 (0.19 to 0.57) | 10.53 |
| Bolivia | 0.19 (-0.16 to 0.56) | 0.22 (-0.16 to 0.62) | 0.17 (-0.14 to 0.49) | 0.18 (-0.17 to 0.58) | -10.53 |
| Brazil | 0.35 (0.15 to 0.52) | 0.34 (0.16 to 0.54) | 0.34 (0.14 to 0.50) | 0.36 (0.16 to 0.54) | 2.86 |
| Colombia | 0.14 (0.02 to 0.25) | 0.15 (0.03 to 0.28) | 0.11 (0.01 to 0.20) | 0.16 (0.02 to 0.28) | 3.57 |
| Costa Rica | 0.25 (0.07 to 0.43) | 0.21 (0.05 to 0.37) | 0.21 (0.05 to 0.36) | 0.32 (0.09 to 0.54) | 22.00 |
| Cuba | 0.37 (0.16 to 0.59) | 0.42 (0.17 to 0.62) | 0.34 (0.15 to 0.54) | 0.34 (0.16 to 0.60) | -10.81 |
| Dominican Republic | 0.22 (0.08 to 0.38) | 0.21 (0.07 to 0.32) | 0.27 (0.11 to 0.48) | 0.19 (0.06 to 0.33) | -4.55 |
| Ecuador | 0.12 (0.01 to 0.22) | 0.13 (0.01 to 0.23) | 0.08 (0.01 to 0.16) | 0.14 (0.01 to 0.27) | 4.17 |
| Guatemala | 0.16 (0.01 to 0.30) | 0.20 (0.01 to 0.38) | 0.14 (0.01 to 0.26) | 0.14 (0.00 to 0.26) | -18.75 |
| Honduras | 0.25 (0.10 to 0.40) | 0.30 (0.12 to 0.47) | 0.24 (0.09 to 0.38) | 0.22 (0.09 to 0.37) | -16.00 |
| Haiti | 0.22 (0.11 to 0.35) | 0.19 (0.09 to 0.27) | 0.33 (0.17 to 0.54) | 0.15 (0.07 to 0.24) | -9.09 |
| Jamaica | 0.36 (0.13 to 0.59) | 0.35 (0.13 to 0.58) | 0.39 (0.13 to 0.61) | 0.35 (0.12 to 0.57) | 0.00 |
| Mexico | 0.51 (0.27 to 0.75) | 0.60 (0.30 to 0.83) | 0.46 (0.27 to 0.71) | 0.49 (0.25 to 0.72) | -10.78 |
| Nicaragua | 0.44 (0.26 to 0.66) | 0.45 (0.24 to 0.61) | 0.45 (0.26 to 0.66) | 0.43 (0.28 to 0.71) | -2.27 |
| Panama | 0.32 (0.14 to 0.49) | 0.31 (0.15 to 0.49) | 0.25 (0.11 to 0.39) | 0.39 (0.17 to 0.57) | 12.50 |
| Peru | 0.21 (-0.20 to 0.63) | 0.26 (-0.21 to 0.72) | 0.17 (-0.16 to 0.54) | 0.18 (-0.21 to 0.61) | -19.05 |
| Paraguay | 0.58 (0.40 to 0.75) | 0.57 (0.40 to 0.76) | 0.56 (0.39 to 0.74) | 0.62 (0.40 to 0.74) | 4.31 |
| El Salvador | 0.25 (0.10 to 0.41) | 0.29 (0.12 to 0.52) | 0.27 (0.11 to 0.45) | 0.19 (0.07 to 0.26) | -20.00 |
| Uruguay | 0.36 (0.20 to 0.51) | 0.30 (0.17 to 0.46) | 0.38 (0.21 to 0.55) | 0.41 (0.20 to 0.52) | 15.28 |
| Venezuela, RB | 0.30 (0.14 to 0.45) | 0.32 (0.17 to 0.54) | 0.24 (0.11 to 0.35) | 0.34 (0.15 to 0.46) | 3.33 |
| **Europe** | 1.97 (1.71 to 2.22) | 1.95 (1.73 to 2.25) | 1.85 (1.60 to 2.09) | 2.13 (1.79 to 2.34) | 4.57 |
| **Northern Europe** | 1.35 (1.12 to 1.56) | 1.39 (1.17 to 1.62) | 1.33 (1.09 to 1.52) | 1.33 (1.09 to 1.52) | -2.22 |
| Denmark | 1.47 (1.25 to 1.68) | 1.54 (1.35 to 1.80) | 1.48 (1.25 to 1.68) | 1.35 (1.11 to 1.51) | -6.46 |
| Estonia | 1.59 (1.30 to 1.86) | 1.68 (1.39 to 1.98) | 1.27 (1.06 to 1.51) | 1.84 (1.48 to 2.12) | 5.03 |
| Finland | 1.58 (1.30 to 1.84) | 1.65 (1.38 to 1.96) | 1.31 (1.09 to 1.55) | 1.77 (1.41 to 2.00) | 3.80 |
| United Kingdom | 1.25 (1.04 to 1.44) | 1.26 (1.06 to 1.46) | 1.27 (1.04 to 1.44) | 1.23 (1.03 to 1.42) | -1.20 |
| Ireland | 0.99 (0.77 to 1.19) | 1.00 (0.80 to 1.23) | 1.01 (0.77 to 1.19) | 0.94 (0.74 to 1.14) | -3.03 |
| Lithuania | 1.78 (1.50 to 2.06) | 1.90 (1.60 to 2.20) | 1.58 (1.35 to 1.85) | 1.86 (1.52 to 2.09) | -1.12 |
| Latvia | 1.71 (1.42 to 1.99) | 1.78 (1.49 to 2.08) | 1.50 (1.26 to 1.76) | 1.87 (1.52 to 2.13) | 2.63 |
| Norway | 1.40 (1.12 to 1.70) | 1.52 (1.21 to 1.82) | 1.50 (1.18 to 1.79) | 1.14 (0.92 to 1.43) | -13.57 |
| Sweden | 1.54 (1.27 to 1.79) | 1.60 (1.37 to 1.92) | 1.56 (1.26 to 1.77) | 1.46 (1.17 to 1.65) | -4.55 |
| **Southern Europe** | 2.30 (2.03 to 2.56) | 2.21 (1.99 to 2.51) | 2.31 (2.03 to 2.56) | 2.40 (2.08 to 2.62) | 4.13 |
| Albania | 2.42 (2.11 to 2.70) | 2.29 (2.01 to 2.58) | 2.41 (2.13 to 2.73) | 2.61 (2.22 to 2.85) | 6.61 |
| Bosnia and Herzegovina | 2.09 (1.84 to 2.35) | 1.87 (1.71 to 2.19) | 2.23 (1.93 to 2.48) | 2.29 (1.94 to 2.49) | 10.05 |
| Spain | 2.19 (1.94 to 2.44) | 2.18 (1.98 to 2.49) | 2.16 (1.91 to 2.41) | 2.23 (1.93 to 2.43) | 1.14 |
| Greece | 2.56 (2.29 to 2.82) | 2.41 (2.19 to 2.69) | 2.43 (2.17 to 2.67) | 2.86 (2.51 to 3.10) | 8.79 |
| Croatia | 2.28 (2.05 to 2.53) | 2.06 (1.91 to 2.36) | 2.43 (2.15 to 2.66) | 2.44 (2.12 to 2.62) | 8.33 |
| Italy | 2.46 (2.16 to 2.75) | 2.39 (2.13 to 2.71) | 2.53 (2.19 to 2.79) | 2.48 (2.16 to 2.76) | 1.83 |
| North Macedonia | 2.25 (1.98 to 2.52) | 2.23 (1.95 to 2.49) | 2.03 (1.86 to 2.36) | 2.55 (2.16 to 2.75) | 7.11 |
| Malta | 2.53 (2.22 to 2.81) | 2.51 (2.25 to 2.84) | 2.54 (2.20 to 2.77) | 2.56 (2.21 to 2.81) | 0.99 |
| Montenegro | 2.35 (2.02 to 2.64) | 2.21 (1.93 to 2.52) | 2.32 (2.00 to 2.62) | 2.53 (2.15 to 2.81) | 6.81 |
| Portugal | 1.93 (1.73 to 2.13) | 1.94 (1.77 to 2.18) | 1.88 (1.70 to 2.09) | 1.96 (1.71 to 2.10) | 0.52 |
| Serbia | 2.20 (1.91 to 2.47) | 2.01 (1.78 to 2.30) | 2.18 (1.93 to 2.49) | 2.50 (2.09 to 2.70) | 11.14 |
| Slovenia | 2.05 (1.83 to 2.28) | 1.92 (1.76 to 2.19) | 2.05 (1.85 to 2.30) | 2.25 (1.93 to 2.40) | 8.05 |
| **Western Europe** | 1.75 (1.53 to 1.96) | 1.80 (1.61 to 2.06) | 1.73 (1.49 to 1.92) | 1.72 (1.46 to 1.89) | -2.29 |
| Austria | 1.96 (1.63 to 2.31) | 1.85 (1.60 to 2.27) | 1.89 (1.55 to 2.19) | 2.19 (1.75 to 2.48) | 8.67 |
| Belgium | 1.59 (1.41 to 1.77) | 1.67 (1.52 to 1.90) | 1.60 (1.40 to 1.75) | 1.49 (1.29 to 1.62) | -5.66 |
| Switzerland | 2.16 (1.67 to 2.66) | 2.11 (1.71 to 2.69) | 2.21 (1.65 to 2.63) | 2.16 (1.65 to 2.64) | 1.16 |
| Germany | 1.68 (1.48 to 1.87) | 1.74 (1.57 to 1.98) | 1.63 (1.42 to 1.79) | 1.65 (1.43 to 1.80) | -2.68 |
| France | 1.87 (1.64 to 2.07) | 1.90 (1.71 to 2.15) | 1.88 (1.64 to 2.07) | 1.81 (1.56 to 1.97) | -2.41 |
| Luxembourg | 1.84 (1.56 to 2.12) | 1.95 (1.69 to 2.30) | 1.78 (1.48 to 2.02) | 1.79 (1.49 to 2.03) | -4.35 |
| Netherlands | 1.55 (1.35 to 1.75) | 1.64 (1.46 to 1.89) | 1.54 (1.35 to 1.75) | 1.45 (1.23 to 1.59) | -6.13 |
| **Eastern Europe** | 2.06 (1.77 to 2.35) | 2.04 (1.79 to 2.38) | 1.85 (1.60 to 2.13) | 2.36 (1.95 to 2.60) | 7.77 |
| Bulgaria | 2.13 (1.88 to 2.40) | 1.88 (1.71 to 2.19) | 2.13 (1.88 to 2.42) | 2.52 (2.11 to 2.71) | 15.02 |
| Belarus | 1.98 (1.69 to 2.30) | 2.07 (1.84 to 2.49) | 1.71 (1.45 to 1.97) | 2.22 (1.80 to 2.44) | 3.79 |
| Czech Republic | 1.73 (1.51 to 1.93) | 1.62 (1.48 to 1.89) | 1.71 (1.48 to 1.88) | 1.89 (1.61 to 2.04) | 7.80 |
| Hungary | 2.19 (1.96 to 2.42) | 1.98 (1.84 to 2.27) | 2.23 (1.97 to 2.44) | 2.46 (2.13 to 2.63) | 10.96 |
| Moldova | 2.31 (2.02 to 2.61) | 2.27 (2.04 to 2.64) | 2.03 (1.77 to 2.28) | 2.74 (2.33 to 3.00) | 10.17 |
| Poland | 1.70 (1.49 to 1.93) | 1.66 (1.50 to 1.94) | 1.61 (1.41 to 1.82) | 1.87 (1.56 to 2.02) | 6.18 |
| Romania | 2.22 (1.94 to 2.51) | 2.04 (1.83 to 2.36) | 2.13 (1.88 to 2.42) | 2.64 (2.21 to 2.85) | 13.51 |
| Russian Federation | 2.02 (1.71 to 2.31) | 2.02 (1.74 to 2.36) | 1.78 (1.52 to 2.06) | 2.34 (1.92 to 2.60) | 7.92 |
| Slovak Republic | 1.99 (1.73 to 2.25) | 1.82 (1.64 to 2.13) | 1.95 (1.72 to 2.23) | 2.26 (1.88 to 2.44) | 11.06 |
| Ukraine | 2.35 (2.01 to 2.68) | 2.42 (2.13 to 2.83) | 2.03 (1.75 to 2.33) | 2.65 (2.19 to 2.91) | 4.89 |
| **Africa** | 0.67 (0.38 to 0.94) | 0.73 (0.42 to 1.06) | 0.60 (0.33 to 0.83) | 0.70 (0.40 to 0.96) | -2.24 |
| **Northern Africa** | 1.20 (0.83 to 1.53) | 1.14 (0.81 to 1.50) | 1.14 (0.79 to 1.45) | 1.29 (0.89 to 1.63) | 6.25 |
| Algeria | 1.12 (0.74 to 1.47) | 1.02 (0.71 to 1.41) | 1.18 (0.76 to 1.52) | 1.17 (0.75 to 1.48) | 6.70 |
| Egypt, Arab Rep. | 1.15 (0.78 to 1.48) | 1.12 (0.79 to 1.48) | 1.05 (0.72 to 1.36) | 1.27 (0.84 to 1.59) | 6.52 |
| Libya | 1.05 (0.74 to 1.35) | 1.12 (0.82 to 1.50) | 1.00 (0.68 to 1.25) | 1.05 (0.73 to 1.33) | -3.33 |
| Morocco | 0.90 (0.52 to 1.24) | 0.82 (0.52 to 1.22) | 0.97 (0.53 to 1.28) | 0.90 (0.50 to 1.21) | 4.44 |
| Sudan | 1.55 (1.20 to 1.92) | 1.57 (1.20 to 1.91) | 1.39 (1.07 to 1.72) | 1.67 (1.30 to 2.09) | 3.23 |
| Tunisia | 1.21 (0.82 to 1.59) | 1.12 (0.79 to 1.53) | 1.30 (0.85 to 1.65) | 1.22 (0.82 to 1.59) | 4.13 |
| **Sub-Saharan Africa** | 0.59 (0.31 to 0.85) | 0.67 (0.36 to 0.98) | 0.52 (0.27 to 0.74) | 0.60 (0.32 to 0.85) | -5.93 |
| Angola | 0.45 (0.26 to 0.62) | 0.57 (0.34 to 0.83) | 0.35 (0.20 to 0.47) | 0.45 (0.25 to 0.59) | -13.33 |
| Burundi | 0.11 (-0.03 to 0.24) | 0.14 (-0.03 to 0.22) | 0.13 (-0.05 to 0.31) | 0.04 (-0.03 to 0.19) | -45.45 |
| Benin | 0.93 (0.51 to 1.33) | 1.02 (0.57 to 1.49) | 0.77 (0.41 to 1.06) | 1.03 (0.56 to 1.46) | 0.54 |
| Burkina Faso | 1.40 (0.97 to 1.81) | 1.39 (1.02 to 1.90) | 1.38 (0.92 to 1.70) | 1.41 (0.98 to 1.84) | 0.71 |
| Botswana | 0.91 (0.57 to 1.23) | 1.08 (0.68 to 1.48) | 0.81 (0.54 to 1.15) | 0.88 (0.51 to 1.08) | -10.99 |
| Central African Republic | 0.58 (0.31 to 0.84) | 0.67 (0.36 to 1.00) | 0.51 (0.27 to 0.74) | 0.58 (0.30 to 0.82) | -7.76 |
| Cote d'Ivoire | 0.71 (0.37 to 1.03) | 0.74 (0.40 to 1.11) | 0.67 (0.33 to 0.93) | 0.72 (0.37 to 1.06) | -1.41 |
| Cameroon | 0.68 (0.38 to 0.95) | 0.72 (0.42 to 1.08) | 0.59 (0.33 to 0.81) | 0.74 (0.40 to 1.00) | 1.47 |
| Congo, Dem. Rep. | 0.26 (0.11 to 0.42) | 0.33 (0.13 to 0.51) | 0.21 (0.09 to 0.35) | 0.26 (0.11 to 0.42) | -13.46 |
| Congo, Rep. | 0.32 (0.13 to 0.50) | 0.34 (0.15 to 0.56) | 0.25 (0.10 to 0.38) | 0.37 (0.14 to 0.57) | 4.69 |
| Djibouti | 1.43 (1.11 to 1.78) | 1.59 (1.19 to 1.93) | 1.09 (0.83 to 1.34) | 1.61 (1.29 to 2.06) | 0.70 |
| Eritrea | 0.54 (0.29 to 0.78) | 0.44 (0.20 to 0.71) | 0.54 (0.29 to 0.74) | 0.64 (0.36 to 0.88) | 18.52 |
| Ethiopia | 0.32 (0.11 to 0.54) | 0.34 (0.11 to 0.57) | 0.28 (0.09 to 0.48) | 0.36 (0.12 to 0.56) | 3.12 |
| Gabon | 0.37 (0.14 to 0.58) | 0.41 (0.16 to 0.68) | 0.38 (0.13 to 0.57) | 0.32 (0.12 to 0.52) | -12.16 |
| Ghana | 0.57 (0.32 to 0.81) | 0.72 (0.41 to 1.06) | 0.47 (0.25 to 0.65) | 0.56 (0.30 to 0.78) | -14.04 |
| Guinea | 0.69 (0.42 to 0.95) | 0.64 (0.42 to 0.94) | 0.73 (0.42 to 0.97) | 0.69 (0.41 to 0.93) | 3.62 |
| Gambia, The | 0.55 (0.36 to 0.70) | 0.53 (0.38 to 0.74) | 0.40 (0.27 to 0.53) | 0.68 (0.43 to 0.83) | 13.64 |
| Guinea-Bissau | 0.73 (0.47 to 1.01) | 0.73 (0.52 to 1.14) | 0.45 (0.29 to 0.62) | 1.04 (0.60 to 1.30) | 21.23 |
| Kenya | 0.34 (0.02 to 0.64) | 0.33 (0.02 to 0.65) | 0.30 (0.02 to 0.59) | 0.39 (0.02 to 0.69) | 8.82 |
| Liberia | 0.42 (0.24 to 0.61) | 0.60 (0.33 to 0.85) | 0.27 (0.15 to 0.39) | 0.41 (0.23 to 0.60) | -22.62 |
| Lesotho | 0.31 (0.03 to 0.57) | 0.34 (0.06 to 0.72) | 0.27 (0.03 to 0.53) | 0.32 (0.01 to 0.52) | -3.23 |
| Madagascar | 0.37 (0.11 to 0.65) | 0.43 (0.11 to 0.67) | 0.36 (0.12 to 0.69) | 0.34 (0.10 to 0.59) | -12.16 |
| Mali | 1.33 (0.92 to 1.69) | 1.40 (0.98 to 1.83) | 1.02 (0.73 to 1.32) | 1.54 (1.04 to 1.88) | 5.26 |
| Mozambique | 0.55 (0.26 to 0.82) | 0.66 (0.32 to 1.00) | 0.58 (0.27 to 0.84) | 0.46 (0.22 to 0.70) | -18.18 |
| Mauritania | 0.81 (0.61 to 1.00) | 0.84 (0.65 to 1.05) | 0.74 (0.55 to 0.90) | 0.87 (0.65 to 1.04) | 1.85 |
| Mauritius | 0.56 (0.11 to 0.99) | 0.57 (0.11 to 1.02) | 0.46 (0.10 to 0.90) | 0.65 (0.12 to 1.07) | 7.14 |
| Malawi | 0.57 (0.26 to 0.88) | 0.78 (0.35 to 1.16) | 0.54 (0.24 to 0.83) | 0.39 (0.20 to 0.67) | -34.21 |
| Namibia | 0.71 (0.43 to 1.00) | 0.75 (0.50 to 1.16) | 0.67 (0.42 to 0.97) | 0.74 (0.40 to 0.92) | -0.70 |
| Niger | 1.76 (1.29 to 2.19) | 1.80 (1.35 to 2.29) | 1.71 (1.21 to 2.05) | 1.77 (1.31 to 2.23) | -0.85 |
| Nigeria | 0.80 (0.48 to 1.10) | 1.10 (0.66 to 1.54) | 0.68 (0.40 to 0.91) | 0.67 (0.40 to 0.92) | -26.88 |
| Rwanda | 0.10 (-0.06 to 0.22) | 0.14 (-0.06 to 0.24) | 0.05 (-0.05 to 0.21) | 0.03 (-0.05 to 0.21) | -55.00 |
| Senegal | 0.75 (0.51 to 0.97) | 0.80 (0.57 to 1.07) | 0.58 (0.39 to 0.75) | 0.87 (0.57 to 1.09) | 4.67 |
| Sierra Leone | 0.68 (0.41 to 0.92) | 0.65 (0.41 to 0.92) | 0.74 (0.43 to 0.97) | 0.64 (0.39 to 0.88) | -0.74 |
| Somalia | 1.02 (0.72 to 1.31) | 1.11 (0.79 to 1.42) | 0.92 (0.63 to 1.16) | 1.04 (0.74 to 1.36) | -3.43 |
| Eswatini | 0.48 (0.26 to 0.67) | 0.53 (0.30 to 0.76) | 0.54 (0.28 to 0.74) | 0.38 (0.20 to 0.54) | -15.63 |
| Chad | 1.48 (1.05 to 1.87) | 1.58 (1.15 to 2.05) | 1.32 (0.93 to 1.65) | 1.56 (1.10 to 1.96) | -0.68 |
| Togo | 0.77 (0.42 to 1.11) | 0.87 (0.49 to 1.30) | 0.62 (0.33 to 0.86) | 0.84 (0.45 to 1.21) | -1.95 |
| Tanzania | 0.52 (0.19 to 0.80) | 0.41 (0.17 to 0.72) | 0.46 (0.17 to 0.71) | 0.67 (0.24 to 0.95) | 25.00 |
| Uganda | 0.23 (-0.03 to 0.50) | 0.26 (-0.04 to 0.52) | 0.17 (-0.02 to 0.41) | 0.28 (-0.03 to 0.58) | 4.35 |
| South Africa | 0.47 (0.20 to 0.71) | 0.49 (0.23 to 0.79) | 0.42 (0.19 to 0.66) | 0.51 (0.21 to 0.72) | 2.13 |
| Zambia | 0.52 (0.25 to 0.78) | 0.73 (0.33 to 1.01) | 0.43 (0.23 to 0.69) | 0.42 (0.22 to 0.66) | -29.81 |
| Zimbabwe | 0.44 (0.21 to 0.66) | 0.55 (0.26 to 0.84) | 0.43 (0.20 to 0.63) | 0.36 (0.18 to 0.54) | -21.59 |
| **Asia** | 0.87 (0.60 to 1.13) | 0.86 (0.60 to 1.13) | 0.83 (0.58 to 1.09) | 0.91 (0.61 to 1.17) | 2.87 |
| **Central Asia** | 0.96 (0.62 to 1.27) | 0.86 (0.58 to 1.18) | 0.92 (0.61 to 1.23) | 1.09 (0.68 to 1.39) | 11.98 |
| Kazakhstan | 0.80 (0.48 to 1.09) | 0.75 (0.48 to 1.07) | 0.73 (0.45 to 1.02) | 0.94 (0.53 to 1.19) | 11.87 |
| Kyrgyz Republic | 0.59 (0.23 to 0.93) | 0.48 (0.21 to 0.85) | 0.58 (0.23 to 0.93) | 0.71 (0.24 to 1.01) | 19.49 |
| Tajikistan | 0.88 (0.52 to 1.22) | 0.81 (0.48 to 1.13) | 0.89 (0.51 to 1.21) | 0.92 (0.55 to 1.31) | 6.25 |
| Turkmenistan | 1.19 (0.81 to 1.52) | 1.04 (0.74 to 1.38) | 1.13 (0.78 to 1.46) | 1.40 (0.92 to 1.73) | 15.13 |
| Uzbekistan | 1.09 (0.75 to 1.40) | 0.99 (0.71 to 1.31) | 1.05 (0.74 to 1.38) | 1.20 (0.80 to 1.50) | 9.63 |
| **Southern Asia** | 1.06 (0.75 to 1.36) | 1.08 (0.77 to 1.38) | 1.02 (0.72 to 1.31) | 1.08 (0.77 to 1.39) | 0.00 |
| Afghanistan | 1.01 (0.68 to 1.28) | 0.82 (0.60 to 1.09) | 0.98 (0.66 to 1.24) | 1.13 (0.74 to 1.43) | 15.35 |
| Bangladesh | 0.46 (0.30 to 0.61) | 0.44 (0.29 to 0.60) | 0.45 (0.29 to 0.60) | 0.50 (0.31 to 0.66) | 6.52 |
| India | 1.12 (0.80 to 1.44) | 1.17 (0.84 to 1.50) | 1.06 (0.75 to 1.37) | 1.13 (0.80 to 1.44) | -1.79 |
| Iran, Islamic Rep. | 1.03 (0.73 to 1.32) | 0.90 (0.63 to 1.15) | 1.03 (0.72 to 1.31) | 1.18 (0.83 to 1.53) | 13.59 |
| Sri Lanka | 0.32 (0.14 to 0.47) | 0.31 (0.16 to 0.50) | 0.24 (0.10 to 0.34) | 0.42 (0.18 to 0.58) | 17.19 |
| Nepal | 0.50 (0.27 to 0.72) | 0.55 (0.33 to 0.79) | 0.44 (0.22 to 0.66) | 0.49 (0.24 to 0.72) | -6.00 |
| Pakistan | 1.24 (0.93 to 1.57) | 1.18 (0.87 to 1.47) | 1.30 (0.96 to 1.61) | 1.23 (0.95 to 1.60) | 2.02 |
| **Western Asia** | 1.00 (0.70 to 1.28) | 0.81 (0.58 to 1.09) | 0.97 (0.68 to 1.23) | 1.18 (0.81 to 1.48) | 18.50 |
| United Arab Emirates | 1.77 (1.21 to 2.26) | 1.81 (1.26 to 2.35) | 1.42 (1.02 to 1.90) | 2.03 (1.35 to 2.52) | 6.21 |
| Armenia | 0.42 (0.12 to 0.71) | 0.33 (0.11 to 0.61) | 0.45 (0.12 to 0.68) | 0.52 (0.16 to 0.88) | 22.62 |
| Azerbaijan | 0.74 (0.42 to 1.02) | 0.51 (0.35 to 0.84) | 0.72 (0.40 to 0.98) | 0.98 (0.51 to 1.25) | 31.76 |
| Cyprus | 0.76 (0.47 to 1.04) | 0.70 (0.46 to 1.02) | 0.81 (0.48 to 1.06) | 0.79 (0.47 to 1.04) | 5.92 |
| Georgia | 0.44 (0.17 to 0.71) | 0.35 (0.15 to 0.62) | 0.46 (0.17 to 0.68) | 0.55 (0.20 to 0.85) | 22.73 |
| Iraq | 1.93 (1.50 to 2.37) | 1.63 (1.29 to 2.04) | 1.99 (1.48 to 2.34) | 2.07 (1.65 to 2.61) | 11.40 |
| Israel | 0.61 (0.38 to 0.81) | 0.57 (0.37 to 0.78) | 0.59 (0.38 to 0.79) | 0.65 (0.41 to 0.87) | 6.56 |
| Jordan | 0.71 (0.41 to 0.99) | 0.66 (0.40 to 0.94) | 0.68 (0.40 to 0.96) | 0.79 (0.44 to 1.06) | 9.15 |
| Kuwait | 2.02 (1.41 to 2.58) | 1.57 (1.20 to 2.21) | 1.48 (1.14 to 2.10) | 2.86 (1.81 to 3.32) | 31.93 |
| Lebanon | 0.45 (0.24 to 0.63) | 0.38 (0.24 to 0.61) | 0.43 (0.25 to 0.64) | 0.51 (0.24 to 0.64) | 14.44 |
| Oman | 1.43 (1.02 to 1.83) | 1.48 (1.05 to 1.89) | 1.37 (0.99 to 1.78) | 1.46 (1.02 to 1.83) | -0.70 |
| West Bank and Gaza | 0.58 (0.35 to 0.78) | 0.54 (0.33 to 0.75) | 0.54 (0.33 to 0.75) | 0.64 (0.37 to 0.85) | 8.62 |
| Saudi Arabia | 1.53 (1.12 to 1.92) | 1.44 (1.10 to 1.89) | 1.43 (1.05 to 1.81) | 1.69 (1.18 to 2.04) | 8.17 |
| Syrian Arab Republic | 0.83 (0.56 to 1.07) | 0.73 (0.53 to 1.00) | 0.83 (0.55 to 1.05) | 0.90 (0.59 to 1.12) | 10.24 |
| Turkey | 0.53 (0.31 to 0.73) | 0.46 (0.30 to 0.69) | 0.53 (0.31 to 0.71) | 0.61 (0.33 to 0.80) | 14.15 |
| Yemen, Rep. | 0.74 (0.46 to 1.02) | 0.55 (0.32 to 0.70) | 0.53 (0.36 to 0.81) | 1.02 (0.63 to 1.41) | 31.76 |
| **Eastern Asia** | 0.75 (0.50 to 0.98) | 0.72 (0.49 to 0.97) | 0.75 (0.50 to 0.98) | 0.80 (0.50 to 0.99) | 5.33 |
| China | 0.77 (0.52 to 1.00) | 0.73 (0.51 to 0.98) | 0.77 (0.52 to 1.01) | 0.80 (0.52 to 1.01) | 4.55 |
| Japan | 0.62 (0.40 to 0.85) | 0.58 (0.41 to 0.86) | 0.56 (0.36 to 0.75) | 0.75 (0.43 to 0.93) | 13.71 |
| Korea, Rep. | 0.66 (0.38 to 0.94) | 0.63 (0.38 to 0.94) | 0.58 (0.36 to 0.88) | 0.81 (0.42 to 1.03) | 13.64 |
| Mongolia | 0.25 (-0.17 to 0.64) | 0.18 (-0.14 to 0.54) | 0.31 (-0.18 to 0.68) | 0.25 (-0.19 to 0.70) | 14.00 |
| Korea, Dem. People's Rep. | 0.52 (0.22 to 0.80) | 0.49 (0.21 to 0.78) | 0.45 (0.20 to 0.74) | 0.66 (0.24 to 0.88) | 16.35 |
| **South-eastern Asia** | 0.44 (0.24 to 0.63) | 0.48 (0.27 to 0.68) | 0.37 (0.21 to 0.55) | 0.47 (0.24 to 0.67) | -1.14 |
| Indonesia | 0.19 (0.06 to 0.32) | 0.16 (0.05 to 0.27) | 0.17 (0.06 to 0.27) | 0.24 (0.08 to 0.40) | 21.05 |
| Cambodia | 0.68 (0.41 to 0.95) | 0.79 (0.47 to 1.08) | 0.61 (0.38 to 0.88) | 0.64 (0.39 to 0.91) | -11.03 |
| Lao PDR | 0.66 (0.40 to 0.92) | 0.77 (0.46 to 1.05) | 0.53 (0.33 to 0.76) | 0.69 (0.40 to 0.93) | -6.06 |
| Myanmar | 0.65 (0.42 to 0.88) | 0.79 (0.51 to 1.06) | 0.46 (0.32 to 0.67) | 0.72 (0.43 to 0.90) | -5.38 |
| Malaysia | 0.34 (0.13 to 0.52) | 0.32 (0.13 to 0.52) | 0.27 (0.11 to 0.44) | 0.41 (0.15 to 0.61) | 13.24 |
| Philippines | 0.43 (0.18 to 0.66) | 0.42 (0.18 to 0.64) | 0.36 (0.16 to 0.58) | 0.50 (0.20 to 0.74) | 9.30 |
| Singapore | 0.42 (0.14 to 0.69) | 0.34 (0.12 to 0.59) | 0.48 (0.15 to 0.78) | 0.43 (0.14 to 0.70) | 10.71 |
| Thailand | 0.88 (0.55 to 1.19) | 0.92 (0.60 to 1.29) | 0.74 (0.49 to 1.05) | 0.99 (0.56 to 1.22) | 3.98 |
| Vietnam | 0.73 (0.48 to 0.99) | 0.83 (0.53 to 1.08) | 0.68 (0.46 to 0.96) | 0.68 (0.45 to 0.94) | -10.27 |
| **Oceania** | 0.57 (-0.06 to 1.21) | 0.68 (-0.06 to 1.44) | 0.52 (-0.07 to 1.07) | 0.52 (-0.07 to 1.14) | -14.04 |
| **Australia and New Zealand** | 0.66 (-0.12 to 1.46) | 0.66 (-0.13 to 1.54) | 0.65 (-0.11 to 1.42) | 0.67 (-0.13 to 1.42) | 0.76 |
| Australia | 0.76 (-0.01 to 1.53) | 0.76 (-0.01 to 1.62) | 0.75 (-0.01 to 1.51) | 0.76 (-0.02 to 1.47) | 0.00 |
| New Zealand | 0.24 (-0.62 to 1.12) | 0.25 (-0.66 to 1.20) | 0.22 (-0.55 to 0.99) | 0.26 (-0.64 to 1.16) | 2.08 |
| **Other regions in Oceania** | 0.46 (0.04 to 0.92) | 0.70 (0.09 to 1.31) | 0.37 (0.02 to 0.69) | 0.37 (0.03 to 0.87) | -35.87 |
| Fiji | 0.89 (-0.18 to 1.90) | 0.67 (-0.13 to 1.56) | 0.99 (-0.20 to 2.26) | 0.97 (-0.19 to 1.83) | 16.85 |
| Papua New Guinea | 0.42 (0.08 to 0.83) | 0.75 (0.13 to 1.34) | 0.31 (0.05 to 0.54) | 0.30 (0.07 to 0.77) | -53.57 |

^a^ $\%Change per decade=\frac{Change per decade}{The mean value in 1990-2019}\times100\%$. Change per decade is calculated using a linear regression.
