# Supplementary material for: Global, regional, and national burden of heatwave-related mortality from 1990 to 2019: A three-stage modelling study
Source: PLoS Med. 2024 May 14;21(5):e1004364. doi: 10.1371/journal.pmed.1004364 (PMC11093289; doi:10.1371/journal.pmed.1004364)
Supplement: S10 Table — (DOCX) [file pmed.1004364.s019.docx]

**S10 Table.** Average excess deaths per ten million residents (based on country-specific population structure) associated with heatwaves per warm season from 1990–1999 to 2010–2019 by continent, region and countries. eCIs=empirical CIs. For country-specific data: To allow comparison, only countries in S6 Table were showed.

|  | **Average** | **1990-1999** | **2000-2009** | **2010–2019** | **%Change per decade ^a^** |
| --- | --- | --- | --- | --- | --- |
| **Global** | 236 (170 to 300) | 261 (191 to 337) | 224 (162 to 285) | 227 (159 to 283) | -7.20 |
| **Americas** | 94 (47 to 139) | 100 (50 to 150) | 84 (42 to 127) | 97 (48 to 143) | -1.60 |
| **Northern America** | 147 (85 to 208) | 158 (90 to 222) | 131 (76 to 190) | 153 (88 to 214) | -1.70 |
| Canada | 41 (-10 to 92) | 43 (-9 to 95) | 39 (-11 to 85) | 41 (-9 to 96) | -2.44 |
| United States | 159 (95 to 221) | 170 (101 to 236) | 141 (86 to 201) | 166 (99 to 227) | -1.26 |
| **Latin American and Caribbean** | 62 (24 to 99) | 65 (24 to 106) | 57 (23 to 91) | 64 (25 to 102) | -0.81 |
| Argentina | 89 (45 to 131) | 78 (42 to 122) | 90 (46 to 133) | 98 (46 to 135) | 11.24 |
| Bolivia | 44 (-36 to 128) | 60 (-45 to 168) | 36 (-30 to 106) | 38 (-35 to 119) | -25.00 |
| Brazil | 70 (31 to 105) | 68 (32 to 109) | 66 (28 to 97) | 74 (33 to 109) | 4.29 |
| Colombia | 23 (3 to 41) | 26 (5 to 48) | 18 (2 to 32) | 25 (3 to 45) | -2.17 |
| Costa Rica | 36 (10 to 61) | 27 (7 to 47) | 27 (7 to 46) | 49 (15 to 84) | 30.56 |
| Cuba | 89 (41 to 146) | 95 (40 to 142) | 79 (36 to 127) | 95 (46 to 169) | 0.00 |
| Dominican Republic | 43 (15 to 72) | 37 (12 to 55) | 50 (20 to 89) | 42 (14 to 71) | 5.81 |
| Ecuador | 20 (2 to 38) | 21 (2 to 38) | 14 (2 to 28) | 25 (2 to 47) | 10.00 |
| Guatemala | 32 (2 to 61) | 51 (3 to 99) | 27 (1 to 50) | 25 (1 to 46) | -40.63 |
| Honduras | 45 (18 to 73) | 57 (23 to 91) | 41 (15 to 64) | 40 (16 to 68) | -18.89 |
| Haiti | 81 (40 to 127) | 77 (36 to 113) | 113 (56 to 183) | 55 (27 to 88) | -13.58 |
| Jamaica | 73 (25 to 118) | 66 (23 to 108) | 76 (26 to 121) | 76 (26 to 124) | 6.85 |
| Mexico | 81 (43 to 119) | 92 (46 to 128) | 68 (40 to 104) | 83 (44 to 124) | -5.56 |
| Nicaragua | 64 (38 to 96) | 68 (37 to 94) | 62 (36 to 90) | 63 (40 to 103) | -3.91 |
| Panama | 48 (21 to 74) | 45 (21 to 71) | 37 (16 to 58) | 61 (27 to 90) | 16.67 |
| Peru | 32 (-31 to 97) | 48 (-39 to 131) | 24 (-23 to 78) | 27 (-31 to 89) | -32.81 |
| Paraguay | 82 (56 to 105) | 74 (53 to 100) | 76 (54 to 102) | 92 (59 to 111) | 10.98 |
| El Salvador | 49 (20 to 80) | 58 (25 to 103) | 51 (21 to 85) | 40 (14 to 55) | -18.37 |
| Uruguay | 108 (58 to 151) | 90 (51 to 135) | 111 (63 to 162) | 122 (60 to 155) | 14.81 |
| Venezuela, RB | 52 (24 to 77) | 51 (28 to 86) | 39 (18 to 58) | 63 (28 to 86) | 11.54 |
| **Europe** | 655 (568 to 739) | 648 (576 to 750) | 630 (547 to 712) | 687 (581 to 756) | 2.98 |
| **Northern Europe** | 407 (337 to 469) | 456 (382 to 532) | 396 (324 to 453) | 373 (307 to 428) | -10.20 |
| Denmark | 458 (389 to 524) | 534 (469 to 627) | 463 (390 to 525) | 383 (315 to 427) | -16.48 |
| Estonia | 617 (505 to 722) | 685 (566 to 808) | 499 (414 to 591) | 659 (530 to 758) | -2.11 |
| Finland | 462 (379 to 537) | 488 (409 to 580) | 374 (311 to 441) | 522 (416 to 590) | 3.68 |
| United Kingdom | 371 (309 to 427) | 415 (349 to 482) | 367 (302 to 419) | 334 (280 to 386) | -10.92 |
| Ireland | 213 (166 to 257) | 260 (206 to 318) | 212 (161 to 249) | 178 (141 to 217) | -19.25 |
| Lithuania | 688 (576 to 792) | 671 (566 to 779) | 617 (525 to 723) | 778 (636 to 876) | 7.78 |
| Latvia | 739 (613 to 856) | 761 (634 to 886) | 651 (546 to 764) | 803 (655 to 916) | 2.84 |
| Norway | 391 (311 to 473) | 480 (382 to 573) | 418 (329 to 501) | 280 (225 to 350) | -25.58 |
| Sweden | 469 (386 to 543) | 520 (445 to 624) | 478 (387 to 544) | 414 (333 to 469) | -11.30 |
| **Southern Europe** | 668 (588 to 741) | 628 (565 to 711) | 661 (580 to 731) | 713 (618 to 778) | 6.36 |
| Albania | 440 (383 to 491) | 355 (310 to 399) | 411 (364 to 467) | 571 (487 to 625) | 24.55 |
| Bosnia and Herzegovina | 566 (494 to 633) | 456 (416 to 533) | 560 (484 to 621) | 708 (599 to 769) | 22.26 |
| Spain | 570 (503 to 634) | 572 (518 to 652) | 557 (492 to 620) | 581 (501 to 633) | 0.79 |
| Greece | 760 (677 to 834) | 654 (594 to 731) | 681 (607 to 748) | 935 (822 to 1013) | 18.49 |
| Croatia | 791 (707 to 873) | 679 (632 to 780) | 833 (739 to 913) | 875 (760 to 938) | 12.39 |
| Italy | 725 (635 to 809) | 694 (620 to 789) | 735 (636 to 810) | 744 (649 to 828) | 3.45 |
| North Macedonia | 676 (592 to 756) | 596 (522 to 668) | 620 (567 to 723) | 825 (696 to 887) | 16.94 |
| Malta | 579 (508 to 641) | 545 (491 to 619) | 570 (493 to 623) | 620 (537 to 679) | 6.48 |
| Montenegro | 666 (574 to 750) | 510 (447 to 582) | 672 (583 to 760) | 823 (700 to 915) | 23.50 |
| Portugal | 582 (521 to 639) | 588 (537 to 659) | 552 (499 to 613) | 605 (527 to 647) | 1.46 |
| Serbia | 826 (717 to 925) | 678 (601 to 776) | 833 (738 to 951) | 988 (826 to 1066) | 18.77 |
| Slovenia | 585 (521 to 648) | 545 (500 to 623) | 572 (516 to 642) | 636 (545 to 679) | 7.78 |
| **Western Europe** | 507 (441 to 567) | 534 (478 to 613) | 488 (421 to 542) | 500 (427 to 549) | -3.35 |
| Austria | 569 (472 to 668) | 569 (494 to 699) | 527 (433 to 611) | 611 (488 to 693) | 3.69 |
| Belgium | 479 (423 to 530) | 522 (474 to 594) | 477 (417 to 522) | 440 (382 to 479) | -8.56 |
| Switzerland | 544 (420 to 668) | 567 (459 to 722) | 549 (410 to 655) | 515 (393 to 628) | -4.78 |
| Germany | 536 (472 to 595) | 564 (510 to 643) | 502 (438 to 552) | 543 (469 to 591) | -1.96 |
| France | 488 (428 to 540) | 512 (459 to 579) | 482 (421 to 531) | 472 (408 to 514) | -4.10 |
| Luxembourg | 444 (375 to 512) | 556 (480 to 656) | 429 (356 to 488) | 375 (313 to 426) | -20.38 |
| Netherlands | 403 (350 to 453) | 436 (387 to 500) | 398 (348 to 450) | 379 (320 to 415) | -7.07 |
| **Eastern Europe** | 820 (702 to 933) | 783 (689 to 914) | 778 (672 to 892) | 904 (748 to 995) | 7.38 |
| Bulgaria | 1045 (915 to 1173) | 847 (772 to 985) | 1046 (926 to 1190) | 1294 (1085 to 1393) | 21.39 |
| Belarus | 796 (677 to 917) | 774 (690 to 935) | 728 (619 to 839) | 887 (722 to 978) | 7.10 |
| Czech Republic | 564 (493 to 628) | 560 (510 to 650) | 539 (465 to 593) | 592 (504 to 641) | 2.84 |
| Hungary | 890 (794 to 980) | 828 (770 to 950) | 877 (778 to 960) | 967 (836 to 1032) | 7.81 |
| Moldova | 789 (689 to 888) | 733 (660 to 851) | 711 (620 to 798) | 945 (804 to 1035) | 13.43 |
| Poland | 515 (448 to 580) | 506 (457 to 592) | 470 (411 to 532) | 570 (476 to 616) | 6.21 |
| Romania | 820 (714 to 921) | 709 (638 to 823) | 766 (675 to 869) | 1016 (851 to 1099) | 18.72 |
| Russian Federation | 837 (706 to 956) | 804 (692 to 936) | 813 (692 to 938) | 897 (734 to 995) | 5.56 |
| Slovak Republic | 600 (520 to 676) | 545 (490 to 637) | 584 (513 to 665) | 669 (557 to 724) | 10.33 |
| Ukraine | 1050 (899 to 1196) | 1025 (901 to 1198) | 947 (820 to 1090) | 1188 (982 to 1307) | 7.76 |
| **Africa** | 229 (131 to 320) | 314 (181 to 450) | 218 (123 to 303) | 185 (107 to 252) | -28.17 |
| **Northern Africa** | 239 (166 to 305) | 268 (192 to 352) | 225 (155 to 286) | 230 (158 to 289) | -7.95 |
| Algeria | 178 (117 to 233) | 180 (125 to 249) | 180 (116 to 232) | 174 (111 to 221) | -1.69 |
| Egypt, Arab Rep. | 233 (159 to 300) | 260 (182 to 344) | 207 (142 to 268) | 236 (156 to 296) | -5.15 |
| Libya | 139 (98 to 178) | 150 (109 to 200) | 116 (79 to 144) | 150 (105 to 191) | 0.00 |
| Morocco | 183 (106 to 252) | 177 (112 to 266) | 191 (105 to 252) | 180 (101 to 241) | 0.82 |
| Sudan | 384 (296 to 474) | 563 (429 to 686) | 365 (281 to 451) | 310 (242 to 388) | -32.94 |
| Tunisia | 205 (139 to 268) | 181 (128 to 248) | 212 (139 to 269) | 218 (147 to 283) | 9.02 |
| **Sub-Saharan Africa** | 227 (122 to 324) | 327 (178 to 478) | 217 (115 to 308) | 173 (94 to 243) | -33.92 |
| Angola | 161 (95 to 225) | 296 (179 to 432) | 133 (77 to 181) | 109 (62 to 144) | -58.07 |
| Burundi | 49 (-16 to 107) | 90 (-18 to 140) | 64 (-23 to 146) | 11 (-8 to 53) | -80.61 |
| Benin | 327 (179 to 464) | 439 (248 to 644) | 280 (149 to 385) | 297 (162 to 420) | -21.71 |
| Burkina Faso | 578 (401 to 750) | 747 (546 to 1019) | 598 (397 to 738) | 473 (328 to 614) | -23.70 |
| Botswana | 359 (226 to 486) | 420 (267 to 579) | 402 (267 to 571) | 276 (160 to 342) | -20.06 |
| Central African Republic | 337 (178 to 491) | 425 (233 to 641) | 316 (166 to 455) | 296 (151 to 418) | -19.14 |
| Cote d'Ivoire | 265 (138 to 385) | 328 (178 to 497) | 281 (140 to 389) | 211 (110 to 310) | -22.08 |
| Cameroon | 240 (135 to 338) | 286 (165 to 426) | 232 (131 to 323) | 217 (119 to 294) | -14.38 |
| Congo, Dem. Rep. | 96 (39 to 154) | 157 (62 to 243) | 87 (36 to 142) | 71 (29 to 115) | -44.79 |
| Congo, Rep. | 111 (45 to 174) | 156 (67 to 260) | 92 (35 to 140) | 98 (38 to 150) | -26.13 |
| Djibouti | 371 (288 to 462) | 467 (349 to 566) | 302 (231 to 371) | 362 (291 to 464) | -14.15 |
| Eritrea | 163 (85 to 235) | 188 (86 to 303) | 161 (86 to 221) | 149 (85 to 206) | -11.96 |
| Ethiopia | 106 (35 to 176) | 173 (56 to 290) | 97 (32 to 166) | 72 (24 to 115) | -47.64 |
| Gabon | 113 (42 to 178) | 146 (57 to 239) | 124 (44 to 185) | 81 (30 to 130) | -28.76 |
| Ghana | 170 (94 to 241) | 240 (137 to 352) | 150 (81 to 208) | 143 (78 to 199) | -28.53 |
| Guinea | 312 (189 to 430) | 358 (231 to 524) | 343 (199 to 454) | 252 (151 to 343) | -16.99 |
| Gambia, The | 146 (97 to 189) | 170 (121 to 238) | 115 (78 to 154) | 157 (98 to 190) | -4.45 |
| Guinea-Bissau | 300 (193 to 418) | 396 (285 to 623) | 193 (126 to 269) | 325 (187 to 405) | -11.83 |
| Kenya | 94 (5 to 179) | 105 (7 to 208) | 96 (5 to 188) | 86 (4 to 153) | -10.11 |
| Liberia | 160 (90 to 230) | 348 (193 to 492) | 102 (57 to 148) | 105 (60 to 153) | -75.94 |
| Lesotho | 160 (15 to 295) | 128 (22 to 269) | 166 (17 to 330) | 179 (8 to 286) | 15.94 |
| Madagascar | 105 (30 to 182) | 159 (40 to 249) | 102 (33 to 194) | 76 (22 to 133) | -39.52 |
| Mali | 579 (402 to 736) | 820 (577 to 1070) | 464 (331 to 602) | 538 (363 to 659) | -24.35 |
| Mozambique | 244 (116 to 363) | 338 (163 to 514) | 267 (125 to 386) | 168 (81 to 252) | -34.84 |
| Mauritania | 196 (148 to 241) | 271 (211 to 339) | 180 (134 to 221) | 163 (121 to 195) | -27.55 |
| Mauritius | 133 (26 to 234) | 123 (24 to 220) | 105 (22 to 206) | 171 (30 to 279) | 18.05 |
| Malawi | 248 (114 to 383) | 467 (212 to 694) | 266 (120 to 410) | 103 (52 to 176) | -73.39 |
| Namibia | 233 (141 to 327) | 239 (159 to 367) | 265 (165 to 382) | 203 (109 to 255) | -7.73 |
| Niger | 763 (560 to 951) | 1116 (838 to 1421) | 782 (553 to 939) | 571 (424 to 721) | -35.71 |
| Nigeria | 318 (191 to 439) | 548 (331 to 766) | 290 (172 to 391) | 201 (120 to 279) | -54.56 |
| Rwanda | 42 (-25 to 95) | 126 (-55 to 207) | 18 (-19 to 72) | 7 (-10 to 41) | -141.67 |
| Senegal | 205 (139 to 264) | 280 (199 to 378) | 164 (109 to 210) | 190 (125 to 238) | -21.95 |
| Sierra Leone | 312 (190 to 428) | 390 (243 to 550) | 365 (215 to 482) | 225 (138 to 311) | -26.44 |
| Somalia | 416 (293 to 536) | 551 (392 to 707) | 381 (261 to 482) | 356 (253 to 464) | -23.44 |
| Eswatini | 202 (108 to 285) | 158 (87 to 227) | 300 (158 to 413) | 149 (80 to 213) | -2.23 |
| Chad | 655 (466 to 830) | 844 (615 to 1096) | 637 (446 to 795) | 563 (398 to 708) | -21.45 |
| Togo | 250 (135 to 358) | 317 (176 to 472) | 221 (115 to 306) | 230 (123 to 328) | -17.40 |
| Tanzania | 176 (65 to 273) | 190 (78 to 330) | 174 (63 to 272) | 169 (60 to 242) | -5.97 |
| Uganda | 87 (-11 to 186) | 146 (-21 to 290) | 69 (-10 to 168) | 69 (-8 to 144) | -44.25 |
| South Africa | 174 (75 to 264) | 144 (67 to 236) | 194 (86 to 303) | 179 (72 to 249) | 10.06 |
| Zambia | 211 (102 to 314) | 390 (174 to 538) | 200 (105 to 321) | 111 (57 to 174) | -66.11 |
| Zimbabwe | 174 (83 to 260) | 193 (91 to 294) | 215 (101 to 316) | 115 (57 to 173) | -22.41 |
| **Asia** | 192 (131 to 249) | 205 (143 to 270) | 182 (125 to 238) | 190 (126 to 242) | -3.91 |
| **Central Asia** | 217 (140 to 289) | 209 (139 to 289) | 219 (143 to 295) | 222 (137 to 283) | 3.00 |
| Kazakhstan | 234 (141 to 317) | 223 (142 to 319) | 235 (146 to 329) | 242 (136 to 305) | 4.06 |
| Kyrgyz Republic | 127 (49 to 201) | 117 (51 to 206) | 135 (53 to 217) | 129 (43 to 183) | 4.72 |
| Tajikistan | 175 (103 to 245) | 201 (120 to 283) | 171 (99 to 233) | 158 (94 to 225) | -12.29 |
| Turkmenistan | 255 (174 to 326) | 241 (171 to 321) | 241 (166 to 311) | 279 (183 to 344) | 7.45 |
| Uzbekistan | 229 (158 to 294) | 214 (152 to 283) | 235 (165 to 306) | 235 (156 to 293) | 4.59 |
| **Southern Asia** | 257 (182 to 330) | 308 (219 to 394) | 246 (174 to 316) | 230 (163 to 294) | -15.18 |
| Afghanistan | 301 (205 to 384) | 363 (266 to 482) | 325 (219 to 409) | 256 (168 to 323) | -17.77 |
| Bangladesh | 98 (63 to 130) | 122 (81 to 166) | 91 (58 to 120) | 85 (53 to 112) | -18.88 |
| India | 279 (198 to 357) | 339 (242 to 434) | 261 (186 to 337) | 249 (176 to 317) | -16.13 |
| Iran, Islamic Rep. | 151 (106 to 193) | 138 (97 to 177) | 145 (101 to 184) | 166 (117 to 215) | 9.27 |
| Sri Lanka | 66 (30 to 97) | 62 (31 to 101) | 50 (22 to 72) | 85 (36 to 118) | 17.42 |
| Nepal | 113 (61 to 165) | 164 (98 to 234) | 93 (46 to 137) | 98 (48 to 143) | -29.20 |
| Pakistan | 334 (251 to 421) | 380 (281 to 474) | 360 (265 to 447) | 281 (217 to 364) | -14.82 |
| **Western Asia** | 151 (103 to 194) | 142 (100 to 191) | 138 (94 to 177) | 167 (111 to 210) | 8.28 |
| United Arab Emirates | 144 (99 to 184) | 164 (113 to 212) | 109 (78 to 146) | 160 (106 to 199) | -1.39 |
| Armenia | 111 (32 to 185) | 76 (25 to 141) | 114 (29 to 172) | 145 (44 to 246) | 31.08 |
| Azerbaijan | 178 (101 to 245) | 130 (87 to 211) | 167 (94 to 227) | 226 (117 to 288) | 26.97 |
| Cyprus | 161 (99 to 219) | 163 (107 to 235) | 168 (101 to 223) | 154 (92 to 204) | -2.80 |
| Georgia | 167 (64 to 266) | 111 (49 to 201) | 176 (64 to 262) | 225 (82 to 348) | 34.13 |
| Iraq | 312 (243 to 384) | 309 (244 to 386) | 333 (248 to 392) | 298 (238 to 375) | -1.76 |
| Israel | 102 (65 to 138) | 107 (68 to 144) | 100 (64 to 134) | 102 (64 to 136) | -2.45 |
| Jordan | 70 (40 to 96) | 78 (46 to 111) | 69 (41 to 97) | 65 (37 to 87) | -9.29 |
| Kuwait | 151 (105 to 193) | 125 (96 to 176) | 120 (92 to 170) | 191 (121 to 222) | 21.85 |
| Lebanon | 89 (48 to 124) | 71 (44 to 114) | 84 (49 to 125) | 105 (50 to 131) | 19.10 |
| Oman | 174 (124 to 223) | 212 (151 to 271) | 177 (127 to 230) | 147 (103 to 185) | -18.68 |
| West Bank and Gaza | 76 (46 to 104) | 83 (51 to 116) | 81 (50 to 112) | 67 (39 to 90) | -10.53 |
| Saudi Arabia | 192 (140 to 240) | 207 (158 to 270) | 181 (133 to 229) | 192 (134 to 232) | -3.91 |
| Syrian Arab Republic | 134 (90 to 171) | 111 (80 to 153) | 87 (58 to 110) | 188 (123 to 233) | 28.73 |
| Turkey | 89 (52 to 121) | 84 (54 to 126) | 77 (45 to 104) | 102 (56 to 133) | 10.11 |
| Yemen, Rep. | 158 (98 to 219) | 164 (95 to 208) | 118 (80 to 180) | 183 (112 to 254) | 6.01 |
| **Eastern Asia** | 161 (106 to 209) | 148 (102 to 200) | 155 (105 to 204) | 178 (112 to 222) | 9.32 |
| China | 163 (109 to 212) | 152 (106 to 205) | 160 (109 to 210) | 176 (113 to 220) | 7.36 |
| Japan | 163 (103 to 219) | 123 (87 to 183) | 138 (89 to 187) | 227 (132 to 285) | 31.90 |
| Korea, Rep. | 109 (62 to 152) | 101 (61 to 151) | 91 (55 to 137) | 134 (69 to 169) | 15.14 |
| Mongolia | 61 (-41 to 157) | 52 (-39 to 154) | 75 (-44 to 164) | 55 (-41 to 154) | 2.46 |
| Korea, Dem. People's Rep. | 149 (61 to 224) | 145 (63 to 231) | 123 (55 to 203) | 177 (64 to 237) | 10.74 |
| **South-eastern Asia** | 101 (55 to 143) | 114 (65 to 160) | 84 (48 to 123) | 106 (55 to 147) | -3.96 |
| Indonesia | 44 (15 to 71) | 38 (13 to 64) | 39 (13 to 62) | 53 (17 to 86) | 17.05 |
| Cambodia | 184 (112 to 258) | 266 (157 to 361) | 163 (100 to 233) | 144 (88 to 205) | -33.15 |
| Lao PDR | 200 (120 to 275) | 316 (191 to 434) | 156 (98 to 223) | 151 (88 to 203) | -41.25 |
| Myanmar | 218 (140 to 293) | 309 (198 to 414) | 157 (108 to 230) | 193 (116 to 243) | -26.61 |
| Malaysia | 54 (20 to 84) | 49 (19 to 80) | 40 (16 to 64) | 69 (25 to 103) | 18.52 |
| Philippines | 80 (34 to 123) | 76 (32 to 117) | 66 (30 to 105) | 95 (39 to 141) | 11.87 |
| Singapore | 54 (17 to 89) | 49 (16 to 84) | 59 (19 to 96) | 53 (17 to 86) | 3.70 |
| Thailand | 180 (112 to 240) | 181 (119 to 255) | 146 (97 to 207) | 210 (120 to 258) | 8.06 |
| Vietnam | 142 (93 to 193) | 158 (101 to 207) | 128 (87 to 179) | 141 (92 to 195) | -5.99 |
| **Oceania** | 133 (-21 to 286) | 150 (-21 to 331) | 125 (-18 to 264) | 126 (-22 to 272) | -9.02 |
| **Australia and New Zealand** | 137 (-25 to 300) | 142 (-27 to 328) | 134 (-21 to 289) | 137 (-26 to 288) | -1.82 |
| Australia | 154 (-3 to 311) | 159 (-2 to 339) | 151 (-2 to 305) | 152 (-5 to 295) | -2.27 |
| New Zealand | 53 (-134 to 244) | 56 (-151 to 273) | 46 (-116 to 210) | 57 (-137 to 251) | 0.94 |
| **Other regions in Oceania** | 118 (10 to 237) | 181 (26 to 336) | 96 (5 to 181) | 95 (7 to 219) | -36.44 |
| Fiji | 210 (-42 to 443) | 139 (-27 to 324) | 228 (-46 to 520) | 247 (-49 to 465) | 25.71 |
| Papua New Guinea | 108 (19 to 212) | 194 (33 to 345) | 81 (12 to 140) | 74 (16 to 185) | -55.56 |

^a^ $\%Change per decade=\frac{Change per decade}{The mean value in 1990-2019}\times100\%$. Change per decade is calculated using a linear regression.
