# Supplementary material for: Global, regional, and national burden of heatwave-related mortality from 1990 to 2019: A three-stage modelling study
Source: PLoS Med. 2024 May 14;21(5):e1004364. doi: 10.1371/journal.pmed.1004364 (PMC11093289; doi:10.1371/journal.pmed.1004364)
Supplement: S11 Table — (DOCX) [file pmed.1004364.s020.docx]

**S11 Table.** Average excess deaths per ten million residents (based on the age structure of WHO standard population) associated with heatwaves per warm season from 1990–1999 to 2010–2019 by continent, region and countries. eCIs=empirical CIs. For country-specific data: To allow comparison, only countries in S6 Table were showed.

|  | **Average** | **1990-1999** | **2000-2009** | **2010–2019** | **%Change per decade ^a^** |
| --- | --- | --- | --- | --- | --- |
| **Global** | 241 (168 to 312) | 283 (201 to 371) | 231 (161 to 300) | 216 (147 to 276) | -13.90 |
| **Americas** | 83 (39 to 125) | 97 (45 to 147) | 76 (36 to 116) | 77 (36 to 115) | -12.05 |
| **Northern America** | 101 (59 to 143) | 117 (67 to 165) | 92 (53 to 132) | 97 (56 to 135) | -9.90 |
| Canada | 27 (-6 to 60) | 33 (-7 to 72) | 25 (-7 to 55) | 23 (-5 to 54) | -18.52 |
| United States | 109 (66 to 152) | 126 (75 to 175) | 99 (60 to 141) | 105 (63 to 144) | -9.63 |
| **Latin American and Caribbean** | 72 (28 to 115) | 84 (32 to 137) | 67 (27 to 107) | 66 (25 to 104) | -12.50 |
| Argentina | 78 (39 to 114) | 74 (40 to 116) | 79 (40 to 117) | 81 (38 to 111) | 4.49 |
| Bolivia | 56 (-46 to 164) | 77 (-57 to 215) | 46 (-39 to 138) | 48 (-44 to 150) | -25.89 |
| Brazil | 79 (35 to 120) | 88 (42 to 142) | 77 (32 to 112) | 73 (32 to 109) | -9.49 |
| Colombia | 26 (4 to 48) | 35 (7 to 65) | 21 (2 to 38) | 24 (3 to 43) | -21.15 |
| Costa Rica | 41 (11 to 70) | 39 (9 to 67) | 33 (9 to 56) | 49 (14 to 83) | 12.20 |
| Cuba | 70 (32 to 114) | 88 (37 to 131) | 63 (29 to 101) | 60 (29 to 108) | -20.00 |
| Dominican Republic | 51 (18 to 86) | 45 (14 to 68) | 61 (24 to 107) | 47 (16 to 80) | 1.96 |
| Ecuador | 24 (2 to 46) | 28 (3 to 51) | 17 (3 to 34) | 28 (2 to 53) | 0.00 |
| Guatemala | 44 (2 to 84) | 70 (4 to 135) | 38 (2 to 70) | 34 (1 to 63) | -40.91 |
| Honduras | 67 (26 to 108) | 82 (33 to 129) | 62 (23 to 98) | 60 (24 to 101) | -16.42 |
| Haiti | 105 (52 to 165) | 97 (45 to 141) | 150 (74 to 242) | 72 (35 to 116) | -11.90 |
| Jamaica | 75 (26 to 121) | 73 (26 to 120) | 79 (27 to 127) | 72 (24 to 118) | -0.67 |
| Mexico | 103 (55 to 152) | 133 (67 to 185) | 89 (52 to 137) | 93 (49 to 138) | -19.42 |
| Nicaragua | 99 (58 to 148) | 105 (56 to 144) | 102 (58 to 148) | 93 (59 to 153) | -6.06 |
| Panama | 54 (24 to 82) | 56 (27 to 90) | 43 (18 to 66) | 62 (27 to 91) | 5.56 |
| Peru | 38 (-36 to 114) | 61 (-49 to 165) | 28 (-27 to 92) | 29 (-33 to 94) | -42.11 |
| Paraguay | 107 (73 to 137) | 103 (73 to 138) | 102 (72 to 136) | 114 (74 to 138) | 5.14 |
| El Salvador | 58 (23 to 95) | 73 (31 to 129) | 61 (25 to 101) | 41 (15 to 57) | -27.59 |
| Uruguay | 74 (40 to 105) | 68 (39 to 103) | 77 (44 to 113) | 77 (38 to 99) | 6.08 |
| Venezuela, RB | 65 (31 to 97) | 75 (40 to 126) | 51 (24 to 76) | 69 (30 to 95) | -4.62 |
| **Europe** | 414 (359 to 468) | 466 (414 to 539) | 394 (342 to 446) | 381 (321 to 419) | -10.27 |
| **Northern Europe** | 230 (190 to 266) | 277 (232 to 323) | 224 (183 to 256) | 193 (159 to 221) | -18.26 |
| Denmark | 258 (220 to 296) | 318 (279 to 373) | 266 (224 to 302) | 195 (161 to 218) | -23.84 |
| Estonia | 390 (319 to 457) | 508 (420 to 600) | 309 (256 to 366) | 339 (272 to 390) | -21.67 |
| Finland | 256 (211 to 299) | 316 (265 to 376) | 208 (173 to 245) | 246 (196 to 278) | -13.67 |
| United Kingdom | 207 (172 to 238) | 243 (204 to 282) | 206 (169 to 235) | 175 (147 to 203) | -16.43 |
| Ireland | 160 (125 to 193) | 205 (162 to 251) | 162 (124 to 191) | 122 (96 to 149) | -25.94 |
| Lithuania | 451 (378 to 521) | 533 (450 to 619) | 405 (345 to 474) | 407 (333 to 458) | -13.97 |
| Latvia | 463 (384 to 537) | 561 (467 to 653) | 405 (340 to 475) | 409 (334 to 466) | -16.41 |
| Norway | 219 (174 to 265) | 272 (217 to 324) | 234 (184 to 280) | 154 (124 to 193) | -26.94 |
| Sweden | 228 (188 to 264) | 264 (226 to 317) | 233 (189 to 265) | 190 (153 to 216) | -16.23 |
| **Southern Europe** | 365 (322 to 406) | 410 (369 to 465) | 358 (314 to 396) | 330 (285 to 360) | -10.96 |
| Albania | 421 (367 to 471) | 456 (398 to 512) | 405 (359 to 460) | 399 (340 to 437) | -6.77 |
| Bosnia and Herzegovina | 437 (383 to 491) | 459 (418 to 536) | 428 (371 to 475) | 419 (355 to 455) | -4.58 |
| Spain | 311 (275 to 347) | 361 (327 to 412) | 300 (265 to 334) | 279 (240 to 304) | -13.18 |
| Greece | 392 (350 to 431) | 412 (374 to 460) | 360 (321 to 396) | 405 (356 to 439) | -0.89 |
| Croatia | 469 (421 to 520) | 495 (460 to 568) | 481 (427 to 527) | 424 (368 to 455) | -7.57 |
| Italy | 349 (306 to 390) | 395 (353 to 449) | 349 (302 to 385) | 305 (266 to 339) | -12.89 |
| North Macedonia | 570 (501 to 639) | 611 (535 to 684) | 513 (470 to 598) | 590 (498 to 634) | -1.84 |
| Malta | 366 (321 to 405) | 424 (381 to 481) | 361 (313 to 395) | 316 (273 to 346) | -14.75 |
| Montenegro | 506 (436 to 569) | 480 (419 to 546) | 508 (439 to 574) | 530 (451 to 589) | 4.94 |
| Portugal | 322 (289 to 355) | 391 (357 to 438) | 305 (276 to 339) | 273 (238 to 292) | -18.32 |
| Serbia | 547 (477 to 615) | 558 (495 to 639) | 537 (476 to 614) | 546 (456 to 589) | -1.10 |
| Slovenia | 357 (319 to 398) | 411 (377 to 469) | 347 (313 to 389) | 315 (270 to 336) | -13.45 |
| **Western Europe** | 273 (239 to 307) | 328 (294 to 377) | 263 (227 to 292) | 232 (198 to 255) | -17.58 |
| Austria | 310 (257 to 364) | 346 (300 to 424) | 292 (240 to 339) | 293 (234 to 333) | -8.55 |
| Belgium | 260 (230 to 289) | 315 (286 to 358) | 253 (221 to 277) | 217 (188 to 236) | -18.85 |
| Switzerland | 297 (230 to 366) | 341 (276 to 434) | 300 (224 to 357) | 252 (192 to 307) | -14.98 |
| Germany | 273 (241 to 304) | 332 (300 to 378) | 257 (224 to 282) | 230 (199 to 250) | -18.68 |
| France | 277 (244 to 307) | 331 (297 to 374) | 270 (235 to 297) | 236 (204 to 257) | -17.15 |
| Luxembourg | 283 (239 to 325) | 375 (326 to 443) | 269 (225 to 306) | 225 (187 to 254) | -26.50 |
| Netherlands | 242 (211 to 273) | 294 (261 to 337) | 242 (211 to 274) | 196 (165 to 214) | -20.25 |
| **Eastern Europe** | 584 (501 to 666) | 631 (555 to 737) | 548 (473 to 629) | 572 (473 to 630) | -5.05 |
| Bulgaria | 595 (523 to 670) | 566 (516 to 658) | 586 (519 to 666) | 642 (538 to 691) | 6.39 |
| Belarus | 563 (481 to 652) | 622 (555 to 751) | 510 (433 to 587) | 556 (453 to 613) | -5.86 |
| Czech Republic | 346 (304 to 387) | 398 (363 to 462) | 327 (282 to 359) | 315 (268 to 341) | -11.99 |
| Hungary | 540 (484 to 597) | 579 (539 to 665) | 530 (470 to 580) | 510 (441 to 544) | -6.39 |
| Moldova | 656 (575 to 741) | 717 (645 to 832) | 584 (510 to 656) | 662 (563 to 725) | -4.19 |
| Poland | 363 (317 to 410) | 427 (386 to 499) | 323 (283 to 366) | 338 (282 to 366) | -12.26 |
| Romania | 550 (481 to 621) | 579 (520 to 672) | 509 (449 to 578) | 563 (472 to 609) | -1.45 |
| Russian Federation | 632 (534 to 723) | 677 (583 to 789) | 607 (517 to 700) | 611 (500 to 678) | -5.22 |
| Slovak Republic | 437 (380 to 494) | 460 (414 to 538) | 424 (373 to 483) | 427 (355 to 462) | -3.78 |
| Ukraine | 709 (609 to 810) | 761 (668 to 889) | 635 (549 to 731) | 731 (604 to 804) | -2.12 |
| **Africa** | 287 (163 to 403) | 362 (207 to 520) | 273 (153 to 382) | 252 (144 to 346) | -19.16 |
| **Northern Africa** | 335 (233 to 428) | 358 (256 to 471) | 319 (219 to 405) | 333 (229 to 418) | -3.73 |
| Algeria | 246 (162 to 323) | 267 (186 to 369) | 258 (165 to 331) | 220 (141 to 280) | -9.55 |
| Egypt, Arab Rep. | 355 (242 to 457) | 375 (264 to 496) | 323 (222 to 418) | 367 (243 to 461) | -1.13 |
| Libya | 208 (146 to 267) | 234 (170 to 312) | 189 (128 to 235) | 205 (144 to 261) | -6.97 |
| Morocco | 235 (136 to 325) | 239 (151 to 358) | 248 (137 to 328) | 220 (124 to 296) | -4.04 |
| Sudan | 513 (396 to 635) | 631 (481 to 768) | 480 (370 to 593) | 479 (373 to 598) | -14.81 |
| Tunisia | 236 (160 to 310) | 239 (169 to 327) | 252 (165 to 320) | 219 (148 to 285) | -4.24 |
| **Sub-Saharan Africa** | 275 (145 to 396) | 363 (193 to 534) | 261 (136 to 376) | 232 (123 to 328) | -23.82 |
| Angola | 207 (121 to 287) | 338 (204 to 494) | 168 (97 to 229) | 164 (93 to 217) | -42.03 |
| Burundi | 64 (-21 to 142) | 111 (-23 to 173) | 85 (-31 to 194) | 17 (-11 to 79) | -73.44 |
| Benin | 403 (221 to 573) | 491 (278 to 721) | 346 (185 to 477) | 397 (216 to 561) | -11.66 |
| Burkina Faso | 667 (463 to 864) | 786 (573 to 1071) | 683 (454 to 842) | 593 (410 to 769) | -14.47 |
| Botswana | 511 (322 to 692) | 620 (394 to 855) | 558 (370 to 793) | 390 (226 to 482) | -22.50 |
| Central African Republic | 447 (236 to 649) | 538 (294 to 811) | 420 (221 to 605) | 407 (208 to 576) | -14.65 |
| Cote d'Ivoire | 358 (186 to 521) | 420 (227 to 636) | 375 (187 to 520) | 304 (158 to 447) | -16.20 |
| Cameroon | 321 (180 to 451) | 353 (204 to 526) | 305 (171 to 424) | 313 (172 to 425) | -6.23 |
| Congo, Dem. Rep. | 124 (50 to 199) | 181 (71 to 280) | 109 (46 to 179) | 105 (43 to 170) | -30.65 |
| Congo, Rep. | 161 (65 to 254) | 217 (94 to 362) | 132 (51 to 200) | 152 (58 to 231) | -20.19 |
| Djibouti | 525 (408 to 655) | 636 (477 to 773) | 424 (324 to 521) | 532 (429 to 685) | -9.90 |
| Eritrea | 262 (138 to 377) | 268 (123 to 433) | 263 (140 to 360) | 257 (146 to 356) | -2.10 |
| Ethiopia | 145 (47 to 239) | 216 (70 to 362) | 132 (43 to 225) | 111 (36 to 175) | -36.21 |
| Gabon | 156 (58 to 245) | 191 (74 to 314) | 172 (61 to 258) | 118 (43 to 189) | -23.40 |
| Ghana | 235 (130 to 333) | 316 (181 to 464) | 206 (112 to 286) | 209 (113 to 291) | -22.77 |
| Guinea | 344 (208 to 473) | 342 (221 to 500) | 372 (216 to 493) | 321 (193 to 436) | -3.05 |
| Gambia, The | 214 (141 to 276) | 223 (158 to 312) | 162 (110 to 217) | 248 (155 to 301) | 5.84 |
| Guinea-Bissau | 416 (264 to 573) | 475 (342 to 747) | 262 (170 to 366) | 506 (291 to 630) | 3.73 |
| Kenya | 144 (8 to 272) | 147 (10 to 291) | 144 (7 to 282) | 142 (7 to 251) | -1.74 |
| Liberia | 192 (108 to 277) | 350 (194 to 495) | 125 (71 to 182) | 158 (90 to 231) | -50.00 |
| Lesotho | 206 (19 to 381) | 167 (29 to 352) | 211 (22 to 419) | 232 (10 to 370) | 15.78 |
| Madagascar | 150 (43 to 262) | 200 (50 to 314) | 149 (49 to 284) | 121 (35 to 214) | -26.33 |
| Mali | 613 (425 to 776) | 779 (549 to 1017) | 477 (341 to 619) | 623 (420 to 763) | -12.72 |
| Mozambique | 305 (146 to 454) | 365 (177 to 556) | 332 (155 to 480) | 246 (119 to 369) | -19.51 |
| Mauritania | 263 (198 to 322) | 338 (263 to 423) | 238 (177 to 292) | 236 (175 to 283) | -19.39 |
| Mauritius | 141 (27 to 251) | 162 (31 to 289) | 115 (25 to 225) | 147 (26 to 241) | -5.32 |
| Malawi | 312 (145 to 485) | 527 (240 to 784) | 345 (155 to 531) | 158 (80 to 272) | -59.13 |
| Namibia | 325 (196 to 456) | 334 (223 to 514) | 368 (228 to 531) | 284 (152 to 356) | -7.69 |
| Niger | 780 (572 to 972) | 995 (747 to 1267) | 783 (554 to 941) | 669 (497 to 846) | -20.90 |
| Nigeria | 352 (211 to 486) | 559 (338 to 782) | 321 (191 to 433) | 251 (150 to 348) | -43.75 |
| Rwanda | 54 (-33 to 127) | 156 (-68 to 256) | 26 (-26 to 100) | 11 (-16 to 64) | -134.26 |
| Senegal | 271 (184 to 350) | 340 (242 to 458) | 216 (144 to 275) | 274 (181 to 343) | -12.18 |
| Sierra Leone | 350 (213 to 479) | 382 (239 to 539) | 405 (239 to 535) | 289 (177 to 399) | -13.29 |
| Somalia | 562 (396 to 724) | 701 (499 to 899) | 505 (346 to 639) | 516 (367 to 674) | -16.46 |
| Eswatini | 296 (159 to 417) | 245 (137 to 354) | 432 (227 to 594) | 214 (115 to 306) | -5.24 |
| Chad | 729 (518 to 923) | 844 (614 to 1095) | 702 (491 to 876) | 687 (486 to 864) | -10.77 |
| Togo | 343 (185 to 493) | 410 (228 to 610) | 302 (158 to 419) | 334 (179 to 477) | -11.08 |
| Tanzania | 230 (85 to 356) | 224 (91 to 388) | 224 (81 to 350) | 239 (84 to 342) | 3.26 |
| Uganda | 118 (-15 to 253) | 175 (-25 to 348) | 94 (-13 to 228) | 105 (-13 to 219) | -29.66 |
| South Africa | 208 (90 to 316) | 183 (85 to 298) | 231 (103 to 361) | 207 (83 to 288) | 5.77 |
| Zambia | 294 (143 to 440) | 500 (223 to 688) | 282 (148 to 453) | 180 (92 to 282) | -54.42 |
| Zimbabwe | 253 (120 to 379) | 280 (132 to 427) | 304 (143 to 448) | 177 (87 to 266) | -20.36 |
| **Asia** | 234 (161 to 305) | 271 (190 to 357) | 227 (157 to 296) | 211 (142 to 270) | -12.82 |
| **Central Asia** | 319 (207 to 423) | 291 (195 to 400) | 330 (217 to 442) | 332 (208 to 423) | 6.43 |
| Kazakhstan | 263 (159 to 357) | 266 (170 to 380) | 262 (162 to 366) | 261 (147 to 329) | -0.95 |
| Kyrgyz Republic | 171 (66 to 270) | 151 (66 to 267) | 182 (72 to 293) | 177 (60 to 251) | 7.60 |
| Tajikistan | 280 (165 to 391) | 271 (162 to 381) | 280 (162 to 381) | 287 (170 to 407) | 2.86 |
| Turkmenistan | 355 (242 to 454) | 353 (251 to 470) | 349 (241 to 451) | 361 (237 to 445) | 1.13 |
| Uzbekistan | 386 (266 to 496) | 329 (234 to 436) | 408 (286 to 532) | 410 (273 to 511) | 10.49 |
| **Southern Asia** | 342 (242 to 438) | 418 (298 to 536) | 333 (236 to 428) | 292 (207 to 374) | -18.42 |
| Afghanistan | 443 (299 to 564) | 419 (307 to 556) | 463 (311 to 583) | 440 (287 to 555) | 2.37 |
| Bangladesh | 132 (85 to 177) | 165 (110 to 225) | 133 (84 to 175) | 107 (67 to 141) | -21.97 |
| India | 367 (261 to 470) | 468 (334 to 599) | 350 (249 to 452) | 307 (217 to 391) | -21.93 |
| Iran, Islamic Rep. | 203 (143 to 261) | 206 (146 to 265) | 202 (141 to 257) | 202 (142 to 261) | -0.99 |
| Sri Lanka | 74 (34 to 110) | 82 (41 to 133) | 58 (25 to 83) | 83 (35 to 115) | 0.68 |
| Nepal | 149 (79 to 217) | 205 (122 to 293) | 127 (64 to 188) | 131 (65 to 191) | -24.83 |
| Pakistan | 455 (341 to 574) | 470 (349 to 587) | 497 (366 to 616) | 409 (316 to 530) | -6.70 |
| **Western Asia** | 233 (163 to 297) | 220 (158 to 294) | 220 (154 to 277) | 251 (173 to 316) | 6.65 |
| United Arab Emirates | 440 (301 to 562) | 520 (360 to 675) | 399 (286 to 533) | 442 (294 to 549) | -8.86 |
| Armenia | 99 (29 to 168) | 90 (29 to 168) | 101 (26 to 152) | 108 (33 to 183) | 9.09 |
| Azerbaijan | 228 (129 to 315) | 173 (116 to 281) | 223 (125 to 303) | 275 (143 to 352) | 22.37 |
| Cyprus | 125 (77 to 170) | 139 (91 to 203) | 135 (81 to 178) | 106 (64 to 140) | -13.20 |
| Georgia | 118 (46 to 189) | 96 (43 to 173) | 123 (45 to 184) | 138 (50 to 214) | 17.80 |
| Iraq | 503 (390 to 618) | 471 (372 to 589) | 546 (406 to 642) | 487 (389 to 615) | 1.59 |
| Israel | 86 (55 to 116) | 98 (63 to 132) | 84 (54 to 114) | 81 (50 to 108) | -9.88 |
| Jordan | 130 (76 to 181) | 155 (93 to 221) | 133 (78 to 187) | 114 (64 to 153) | -15.77 |
| Kuwait | 277 (193 to 354) | 245 (187 to 345) | 210 (162 to 297) | 349 (220 to 404) | 18.77 |
| Lebanon | 90 (49 to 127) | 86 (53 to 138) | 82 (48 to 123) | 99 (47 to 123) | 7.22 |
| Oman | 398 (283 to 509) | 468 (331 to 599) | 389 (280 to 506) | 360 (253 to 452) | -13.57 |
| West Bank and Gaza | 139 (84 to 189) | 146 (90 to 202) | 141 (87 to 195) | 133 (78 to 177) | -4.68 |
| Saudi Arabia | 372 (271 to 466) | 396 (302 to 518) | 357 (263 to 451) | 371 (259 to 448) | -3.36 |
| Syrian Arab Republic | 208 (140 to 266) | 199 (143 to 274) | 169 (112 to 213) | 246 (161 to 306) | 11.30 |
| Turkey | 101 (59 to 139) | 111 (72 to 167) | 90 (52 to 121) | 103 (56 to 134) | -3.96 |
| Yemen, Rep. | 236 (146 to 328) | 209 (121 to 265) | 170 (115 to 259) | 297 (182 to 411) | 18.64 |
| **Eastern Asia** | 157 (105 to 206) | 179 (123 to 241) | 156 (106 to 206) | 139 (87 to 173) | -12.74 |
| China | 167 (113 to 218) | 191 (133 to 257) | 168 (115 to 221) | 145 (93 to 182) | -13.77 |
| Japan | 76 (49 to 104) | 79 (56 to 118) | 65 (42 to 89) | 84 (49 to 105) | 3.29 |
| Korea, Rep. | 101 (58 to 144) | 128 (77 to 191) | 87 (53 to 131) | 90 (46 to 113) | -18.81 |
| Mongolia | 95 (-64 to 243) | 81 (-61 to 239) | 125 (-73 to 274) | 78 (-58 to 219) | -1.58 |
| Korea, Dem. People's Rep. | 150 (62 to 228) | 162 (71 to 257) | 133 (60 to 220) | 156 (56 to 209) | -2.00 |
| **South-eastern Asia** | 127 (69 to 182) | 154 (86 to 218) | 108 (61 to 160) | 123 (62 to 173) | -12.20 |
| Indonesia | 60 (20 to 97) | 55 (18 to 91) | 54 (18 to 85) | 69 (23 to 112) | 11.67 |
| Cambodia | 253 (153 to 355) | 358 (212 to 486) | 232 (143 to 332) | 195 (120 to 279) | -32.21 |
| Lao PDR | 267 (161 to 368) | 388 (234 to 533) | 217 (136 to 310) | 221 (128 to 296) | -31.27 |
| Myanmar | 269 (173 to 363) | 387 (249 to 518) | 196 (134 to 286) | 232 (140 to 293) | -28.81 |
| Malaysia | 79 (30 to 123) | 84 (33 to 136) | 64 (26 to 102) | 88 (31 to 131) | 2.53 |
| Philippines | 115 (49 to 177) | 112 (48 to 174) | 98 (44 to 157) | 132 (54 to 196) | 8.70 |
| Singapore | 58 (19 to 94) | 64 (21 to 109) | 66 (21 to 106) | 46 (15 to 75) | -15.52 |
| Thailand | 185 (116 to 250) | 233 (152 to 328) | 155 (103 to 220) | 172 (98 to 212) | -16.49 |
| Vietnam | 181 (119 to 246) | 224 (144 to 294) | 164 (112 to 230) | 160 (105 to 221) | -17.68 |
| **Oceania** | 112 (-13 to 240) | 144 (-12 to 306) | 103 (-14 to 214) | 97 (-12 to 212) | -20.98 |
| **Australia and New Zealand** | 92 (-17 to 203) | 109 (-21 to 253) | 89 (-15 to 193) | 82 (-16 to 172) | -14.67 |
| Australia | 103 (-2 to 209) | 122 (-1 to 260) | 100 (-1 to 202) | 91 (-3 to 176) | -15.05 |
| New Zealand | 37 (-93 to 170) | 44 (-119 to 216) | 33 (-82 to 149) | 34 (-83 to 152) | -13.51 |
| **Other regions in Oceania** | 178 (15 to 359) | 270 (35 to 504) | 148 (8 to 279) | 143 (11 to 331) | -35.67 |
| Fiji | 306 (-61 to 651) | 243 (-47 to 564) | 347 (-70 to 789) | 316 (-63 to 595) | 11.93 |
| Papua New Guinea | 161 (29 to 318) | 281 (47 to 498) | 124 (19 to 214) | 114 (26 to 287) | -51.86 |

^a^ $\%Change per decade=\frac{Change per decade}{The mean value in 1990-2019}\times100\%$. Change per decade is calculated using a linear regression.
