# Supplementary material for: Global, regional, and national burden of heatwave-related mortality from 1990 to 2019: A three-stage modelling study
Source: PLoS Med. 2024 May 14;21(5):e1004364. doi: 10.1371/journal.pmed.1004364 (PMC11093289; doi:10.1371/journal.pmed.1004364)
Supplement: S12 Table — (DOCX) [file pmed.1004364.s021.docx]

**S12 Table.** Average excess deaths (based on country-specific population structure) associated with heatwaves per warm season from 1990–1999 to 2010–2019 by the indicators of Köppen-Geiger climate classification and World Bank income groups. eCIs=empirical CIs.

|  | **Average; Proportion%** | **1990-1999** | **2000-2009** | **2010–2019** | **%Change per decade ^a^** |
| --- | --- | --- | --- | --- | --- |
| **Climate zones** |  |  |  |  |  |
| Group A: Tropical climate | 31351 (18201 to 43904); 20.48 | 32539 (19279 to 46121) | 29233 (16930 to 40853) | 32280 (18395 to 44737) | -0.41 |
| Group B: Dry climate | 29486 (20543 to 38070); 19.26 | 28395 (19916 to 36908) | 28350 (19716 to 36462) | 31713 (21998 to 40840) | 5.63 |
| Group C: Temperate climates | 68306 (52812 to 82447); 44.62 | 65414 (51643 to 80575) | 65832 (51205 to 79861) | 73672 (55588 to 86906) | 6.04 |
| Group D: Continental climates | 23364 (17465 to 28648); 15.26 | 22302 (17442 to 28080) | 21952 (16638 to 27311) | 25838 (18314 to 30552) | 7.57 |
| Group E: Polar and alpine climates | 571 (-88 to 1301); 0.37 | 519 (-71 to 1187) | 561 (-83 to 1273) | 634 (-111 to 1443) | 10.07 |
| **Income groups** |  |  |  |  |  |
| Low income | 13244 (7346 to 18867); 8.65 | 13872 (7732 to 20095) | 12450 (6847 to 17855) | 13410 (7458 to 18588) | -1.74 |
| Lower-middle income | 58588 (40612 to 75898); 38.27 | 59928 (41845 to 78026) | 55124 (38230 to 71354) | 60712 (41825 to 78296) | 0.67 |
| Upper-middle income | 47229 (33712 to 59447); 30.85 | 42669 (31615 to 55195) | 46009 (33360 to 58470) | 53011 (36161 to 64677) | 10.95 |
| High income | 34016 (27179 to 40319); 22.22 | 32701 (26961 to 39625) | 32345 (25910 to 38240) | 37003 (28668 to 43092) | 6.32 |

^a^ $\%Change per decade=\frac{Change per decade}{The mean value in 1990-2019}\times100\%$. Change per decade is calculated using a linear regression.
