# Supplementary material for: Global, regional, and national burden of heatwave-related mortality from 1990 to 2019: A three-stage modelling study
Source: PLoS Med. 2024 May 14;21(5):e1004364. doi: 10.1371/journal.pmed.1004364 (PMC11093289; doi:10.1371/journal.pmed.1004364)
Supplement: S13 Table — (DOCX) [file pmed.1004364.s022.docx]

**S13 Table.** Average excess deaths (based on the age structure of WHO standard population) associated with heatwaves per warm season from 1990–1999 to 2010–2019 by the indicators of Köppen-Geiger climate classification and World Bank income groups. eCIs=empirical CIs.

|  | **Average; Proportion%** | **1990-1999** | **2000-2009** | **2010–2019** | **%Change per decade ^a^** |
| --- | --- | --- | --- | --- | --- |
| **Climate zones** |  |  |  |  |  |
| Group A: Tropical climate | 39538 (22909 to 55448); 25.32 | 41316 (24592 to 58439) | 37508 (21695 to 52464) | 39790 (22439 to 55442) | -1.93 |
| Group B: Dry climate | 38556 (26989 to 49710); 24.69 | 36292 (25413 to 47213) | 37423 (26141 to 48078) | 41953 (29412 to 53838) | 7.34 |
| Group C: Temperate climate | 59340 (44339 to 73572); 38.00 | 64198 (48919 to 80616) | 57740 (43310 to 71994) | 56082 (40789 to 68106) | -6.84 |
| Group D: Continental climate | 18267 (13313 to 22841); 11.70 | 19600 (14912 to 25189) | 17261 (12675 to 21905) | 17940 (12352 to 21429) | -4.54 |
| Group E: Polar and alpine climates | 451 (-160 to 1128); 0.29 | 461 (-150 to 1147) | 444 (-158 to 1113) | 447 (-170 to 1124) | -1.55 |
| **Income groups** |  |  |  |  |  |
| Low income | 16709 (9098 to 23969); 10.70 | 16203 (8891 to 23562) | 15793 (8535 to 22844) | 18130 (9867 to 25378) | 5.77 |
| Lower-middle income | 74203 (51058 to 96631); 47.52 | 76231 (52861 to 99512) | 70991 (48840 to 92356) | 75387 (51472 to 98024) | -0.57 |
| Upper-middle income | 44656 (31104 to 57309); 28.60 | 47079 (33894 to 61949) | 44109 (31046 to 56964) | 42778 (28373 to 53015) | -4.82 |
| High income | 20584 (16255 to 24741); 13.18 | 22353 (18180 to 27396) | 19483 (15402 to 23375) | 19916 (15150 to 23452) | -5.92 |

^a^ $\%Change per decade=\frac{Change per decade}{The mean value in 1990-2019}\times100\%$. Change per decade is calculated using a linear regression.
