# Supplementary material for: Global, regional, and national burden of heatwave-related mortality from 1990 to 2019: A three-stage modelling study
Source: PLoS Med. 2024 May 14;21(5):e1004364. doi: 10.1371/journal.pmed.1004364 (PMC11093289; doi:10.1371/journal.pmed.1004364)
Supplement: S14 Table — (DOCX) [file pmed.1004364.s023.docx]

**S14 Table.** Average excess deaths per ten million residents (based on country-specific population structure) associated with heatwaves per warm season from 1990–1999 to 2010–2019 by the indicators of Köppen-Geiger climate classification and World Bank income groups. eCIs=empirical CIs.

|  | **Average** | **1990-1999** | **2000-2009** | **2010–2019** | **%Change per decade ^a^** |
| --- | --- | --- | --- | --- | --- |
| **Climate zones** |  |  |  |  |  |
| Group A: Tropical climate | 214 (124 to 299) | 199 (118 to 283) | 149 (86 to 208) | 142 (81 to 197) | -13.32 |
| Group B: Dry climate | 393 (274 to 508) | 344 (242 to 448) | 285 (198 to 366) | 269 (187 to 347) | -9.54 |
| Group C: Temperate climate | 328 (253 to 396) | 257 (203 to 317) | 235 (183 to 286) | 246 (185 to 290) | -1.68 |
| Group D: Continental climate | 424 (317 to 520) | 317 (248 to 399) | 297 (225 to 369) | 340 (241 to 401) | 2.71 |
| Group E: Polar and alpine climate | 509 (-79 to 1158) | 420 (-57 to 960) | 369 (-55 to 838) | 365 (-64 to 831) | -5.40 |
| **Income groups** |  |  |  |  |  |
| Low income | 243 (135 to 346) | 241 (134 to 349) | 171 (94 to 246) | 153 (85 to 211) | -18.11 |
| Lower-middle income | 334 (232 to 433) | 304 (213 to 396) | 236 (163 to 305) | 225 (155 to 290) | -11.83 |
| Upper-middle income | 266 (190 to 335) | 195 (145 to 252) | 193 (140 to 245) | 210 (143 to 256) | 2.82 |
| High income | 431 (345 to 511) | 333 (274 to 403) | 307 (246 to 363) | 331 (257 to 386) | -0.23 |

^a^ $\%Change per decade=\frac{Change per decade}{The mean value in 1990-2019}\times100\%$. Change per decade is calculated using a linear regression.
