# Supplementary material for: Global, regional, and national burden of heatwave-related mortality from 1990 to 2019: A three-stage modelling study
Source: PLoS Med. 2024 May 14;21(5):e1004364. doi: 10.1371/journal.pmed.1004364 (PMC11093289; doi:10.1371/journal.pmed.1004364)
Supplement: S15 Table — (DOCX) [file pmed.1004364.s024.docx]

**S15 Table.** Average excess deaths per ten million residents (based on the age structure of WHO standard population) associated with heatwaves per warm season from 1990–1999 to 2010–2019 by the indicators of Köppen-Geiger climate classification and World Bank income groups. eCIs=empirical CIs.

|  | **Average** | **1990-1999** | **2000-2009** | **2010–2019** | **%Change per decade ^a^** |
| --- | --- | --- | --- | --- | --- |
| **Climate zones** |  |  |  |  |  |
| Group A: Tropical climate | 269 (156 to 378) | 253 (151 to 358) | 191 (111 to 267) | 175 (99 to 244) | -14.50 |
| Group B: Dry climate | 514 (360 to 663) | 440 (308 to 573) | 376 (262 to 483) | 356 (250 to 457) | -8.17 |
| Group C: Temperate climate | 285 (213 to 353) | 253 (192 to 317) | 206 (155 to 257) | 187 (136 to 227) | -11.58 |
| Group D: Continental climate | 331 (242 to 414) | 278 (212 to 358) | 233 (171 to 296) | 236 (162 to 282) | -6.34 |
| Group E: Polar and alpine climate | 401 (-142 to 1004) | 373 (-121 to 928) | 292 (-104 to 733) | 257 (-98 to 647) | -14.46 |
| **Income groups** |  |  |  |  |  |
| Low income | 306 (167 to 439) | 281 (154 to 409) | 217 (118 to 315) | 206 (112 to 289) | -12.25 |
| Lower-middle income | 423 (291 to 551) | 387 (269 to 505) | 303 (209 to 395) | 279 (190 to 363) | -12.77 |
| Upper-middle income | 251 (175 to 323) | 215 (155 to 283) | 185 (130 to 238) | 169 (112 to 210) | -9.16 |
| High income | 261 (206 to 314) | 227 (185 to 279) | 185 (146 to 222) | 178 (136 to 210) | -9.39 |

^a^ $\%Change per decade=\frac{Change per decade}{The mean value in 1990-2019}\times100\%$. Change per decade is calculated using a linear regression.
