# Supplementary material for: Global, regional, and national burden of heatwave-related mortality from 1990 to 2019: A three-stage modelling study
Source: PLoS Med. 2024 May 14;21(5):e1004364. doi: 10.1371/journal.pmed.1004364 (PMC11093289; doi:10.1371/journal.pmed.1004364)
Supplement: S16 Table — (DOCX) [file pmed.1004364.s025.docx]

**S16 Table.** Average excess death ratio (based on country-specific population structure) associated with heatwaves per warm season from 1990–1999 to 2010–2019 by the indicators of Köppen-Geiger climate classification and World Bank income groups. eCIs=empirical CIs.

|  | **Average** | **1990-1999** | **2000-2009** | **2010–2019** | **%Change per decade ^a^** |
| --- | --- | --- | --- | --- | --- |
| **Climate zones** |  |  |  |  |  |
| Group A: Tropical climate | 0.81 (0.47 to 1.14) | 0.66 (0.39 to 0.94) | 0.56 (0.32 to 0.78) | 0.61 (0.35 to 0.85) | -3.70 |
| Group B: Dry climate | 1.65 (1.15 to 2.13) | 1.25 (0.88 to 1.62) | 1.18 (0.82 to 1.51) | 1.29 (0.90 to 1.66) | 1.21 |
| Group C: Temperate climate | 1.36 (1.05 to 1.64) | 1.02 (0.81 to 1.26) | 0.98 (0.76 to 1.19) | 1.05 (0.79 to 1.24) | 0.74 |
| Group D: Continental climate | 1.54 (1.15 to 1.89) | 1.15 (0.90 to 1.45) | 1.05 (0.80 to 1.31) | 1.26 (0.89 to 1.49) | 3.25 |
| Group E: Polar and alpine climate | 2.21 (-0.34 to 5.04) | 1.69 (-0.23 to 3.86) | 1.65 (-0.24 to 3.74) | 1.65 (-0.29 to 3.75) | -0.90 |
| **Income groups** |  |  |  |  |  |
| Low income | 0.76 (0.42 to 1.08) | 0.58 (0.32 to 0.84) | 0.51 (0.28 to 0.73) | 0.62 (0.35 to 0.86) | 2.63 |
| Lower-middle income | 1.31 (0.91 to 1.70) | 1.04 (0.73 to 1.36) | 0.92 (0.63 to 1.18) | 0.99 (0.68 to 1.28) | -2.29 |
| Upper-middle income | 1.20 (0.86 to 1.51) | 0.89 (0.66 to 1.15) | 0.87 (0.63 to 1.10) | 0.94 (0.64 to 1.15) | 1.67 |
| High income | 1.65 (1.32 to 1.96) | 1.26 (1.04 to 1.53) | 1.20 (0.96 to 1.42) | 1.26 (0.98 to 1.47) | 0.00 |

^a^ $\%Change per decade=\frac{Change per decade}{The mean value in 1990-2019}\times100\%$. Change per decade is calculated using a linear regression.
