# Supplementary material for: Global, regional, and national burden of heatwave-related mortality from 1990 to 2019: A three-stage modelling study
Source: PLoS Med. 2024 May 14;21(5):e1004364. doi: 10.1371/journal.pmed.1004364 (PMC11093289; doi:10.1371/journal.pmed.1004364)
Supplement: S17 Table — (DOCX) [file pmed.1004364.s026.docx]

**S17 Table.** Average excess death ratio (based on the age structure of WHO standard population) associated with heatwaves per warm season from 1990–1999 to 2010–2019 by the indicators of Köppen-Geiger climate classification and World Bank income groups. eCIs=empirical CIs.

|  | **Average** | **1990-1999** | **2000-2009** | **2010–2019** | **%Change per decade ^a^** |
| --- | --- | --- | --- | --- | --- |
| **Climate zones** |  |  |  |  |  |
| Group A: Tropical climate | 0.81 (0.47 to 1.13) | 0.65 (0.39 to 0.93) | 0.56 (0.32 to 0.78) | 0.60 (0.34 to 0.84) | -3.70 |
| Group B: Dry climate | 1.66 (1.16 to 2.14) | 1.24 (0.87 to 1.62) | 1.18 (0.83 to 1.52) | 1.31 (0.92 to 1.68) | 1.81 |
| Group C: Temperate climate | 1.22 (0.91 to 1.51) | 0.94 (0.71 to 1.17) | 0.88 (0.66 to 1.09) | 0.93 (0.68 to 1.13) | -0.82 |
| Group D: Continental climate | 1.40 (1.02 to 1.75) | 1.03 (0.78 to 1.32) | 0.96 (0.71 to 1.22) | 1.17 (0.81 to 1.40) | 5.00 |
| Group E: Polar and alpine climate | 1.76 (-0.62 to 4.41) | 1.38 (-0.45 to 3.42) | 1.30 (-0.46 to 3.25) | 1.30 (-0.49 to 3.26) | -2.27 |
| **Income groups** |  |  |  |  |  |
| Low income | 0.73 (0.40 to 1.05) | 0.56 (0.31 to 0.81) | 0.50 (0.27 to 0.72) | 0.60 (0.33 to 0.84) | 2.74 |
| Lower-middle income | 1.28 (0.88 to 1.66) | 1.01 (0.70 to 1.32) | 0.90 (0.62 to 1.17) | 0.97 (0.66 to 1.26) | -1.56 |
| Upper-middle income | 1.10 (0.77 to 1.42) | 0.82 (0.59 to 1.07) | 0.80 (0.56 to 1.03) | 0.87 (0.58 to 1.08) | 1.82 |
| High income | 1.59 (1.26 to 1.92) | 1.21 (0.99 to 1.49) | 1.15 (0.91 to 1.37) | 1.23 (0.93 to 1.44) | 0.63 |

^a^ $\%Change per decade=\frac{Change per decade}{The mean value in 1990-2019}\times100\%$. Change per decade is calculated using a linear regression.
