# Supplementary material for: Global, regional, and national burden of heatwave-related mortality from 1990 to 2019: A three-stage modelling study
Source: PLoS Med. 2024 May 14;21(5):e1004364. doi: 10.1371/journal.pmed.1004364 (PMC11093289; doi:10.1371/journal.pmed.1004364)
Supplement: S18 Table — (DOCX) [file pmed.1004364.s027.docx]

**S18 Table.** Relative risk (RR) of heatwave-related mortality with 95%confidence interval (CI) for each country from 1990–1999 to 2010–2019. To allow comparison, only countries in S6 Table were showed. The country-specific RRs were pooled with meta-analysis from the effect estimates of grid cells.

|  | **1990-1999** | **2000-2009** | **2010–2019** |
| --- | --- | --- | --- |
| **Global** |  |  |  |
| **Americas** |  |  |  |
| **Northern America** |  |  |  |
| Canada | 1.0043 (1.0039 to 1.0048) | 1.0049 (1.0045 to 1.0053) | 1.0056 (1.0052 to 1.006) |
| United States | 1.0397 (1.0386 to 1.0407) | 1.0397 (1.0387 to 1.0406) | 1.0403 (1.0394 to 1.0413) |
| **Latin American and Caribbean** |  |  |  |
| Argentina | 1.0369 (1.0352 to 1.0386) | 1.0409 (1.0391 to 1.0427) | 1.041 (1.0393 to 1.0428) |
| Bolivia | 1.0442 (1.0415 to 1.0469) | 1.0452 (1.0424 to 1.0481) | 1.0444 (1.0416 to 1.0472) |
| Brazil | 1.0411 (1.0405 to 1.0416) | 1.0461 (1.0455 to 1.0467) | 1.0464 (1.0457 to 1.047) |
| Colombia | 1.0363 (1.034 to 1.0386) | 1.0391 (1.0367 to 1.0415) | 1.0382 (1.0357 to 1.0406) |
| Costa Rica | 1.043 (1.0344 to 1.0516) | 1.0408 (1.0332 to 1.0485) | 1.0391 (1.032 to 1.0464) |
| Cuba | 1.0454 (1.0427 to 1.0482) | 1.0439 (1.0412 to 1.0466) | 1.0389 (1.0362 to 1.0416) |
| Dominican Republic | 1.0367 (1.0322 to 1.0412) | 1.0323 (1.0283 to 1.0364) | 1.0326 (1.0286 to 1.0366) |
| Ecuador | 1.0304 (1.0259 to 1.0349) | 1.0282 (1.0236 to 1.0328) | 1.0244 (1.0209 to 1.0279) |
| Guatemala | 1.0376 (1.032 to 1.0433) | 1.036 (1.0308 to 1.0412) | 1.0374 (1.0316 to 1.0432) |
| Honduras | 1.0421 (1.0375 to 1.0468) | 1.0428 (1.0387 to 1.0468) | 1.0421 (1.0373 to 1.0469) |
| Haiti | 1.0434 (1.0382 to 1.0486) | 1.0388 (1.0337 to 1.044) | 1.0387 (1.0335 to 1.0439) |
| Jamaica | 1.0382 (1.0309 to 1.0456) | 1.0393 (1.032 to 1.0466) | 1.0386 (1.0314 to 1.0459) |
| Mexico | 1.0651 (1.0628 to 1.0675) | 1.0601 (1.0578 to 1.0624) | 1.062 (1.0597 to 1.0643) |
| Nicaragua | 1.0545 (1.0516 to 1.0573) | 1.0523 (1.0495 to 1.0551) | 1.0491 (1.0462 to 1.052) |
| Panama | 1.0358 (1.0327 to 1.0389) | 1.0368 (1.0336 to 1.0401) | 1.0387 (1.0353 to 1.0421) |
| Peru | 1.0306 (1.0284 to 1.0328) | 1.0302 (1.0279 to 1.0325) | 1.0284 (1.0263 to 1.0304) |
| Paraguay | 1.0678 (1.0658 to 1.0699) | 1.0749 (1.0723 to 1.0775) | 1.0761 (1.0744 to 1.0778) |
| El Salvador | 1.0375 (1.0278 to 1.0473) | 1.0401 (1.0322 to 1.048) | 1.0466 (1.0374 to 1.0558) |
| Uruguay | 1.0385 (1.0365 to 1.0405) | 1.0391 (1.0371 to 1.0411) | 1.0448 (1.0426 to 1.0469) |
| Venezuela, RB | 1.0409 (1.0386 to 1.0433) | 1.0456 (1.0432 to 1.0479) | 1.0469 (1.0445 to 1.0494) |
| **Europe** |  |  |  |
| **Northern Europe** |  |  |  |
| Denmark | 1.1101 (1.1058 to 1.1143) | 1.1158 (1.1107 to 1.1208) | 1.1243 (1.1177 to 1.131) |
| Estonia | 1.1276 (1.1244 to 1.1309) | 1.1278 (1.1247 to 1.1309) | 1.1328 (1.1298 to 1.1359) |
| Finland | 1.1097 (1.1082 to 1.1113) | 1.1132 (1.1119 to 1.1145) | 1.1158 (1.1143 to 1.1173) |
| United Kingdom | 1.0945 (1.0909 to 1.0982) | 1.0978 (1.0943 to 1.1012) | 1.0943 (1.0907 to 1.0979) |
| Ireland | 1.0856 (1.0832 to 1.0879) | 1.088 (1.0859 to 1.0902) | 1.0846 (1.0824 to 1.0869) |
| Lithuania | 1.1391 (1.136 to 1.1423) | 1.1387 (1.1357 to 1.1418) | 1.1441 (1.1411 to 1.147) |
| Latvia | 1.1324 (1.1294 to 1.1354) | 1.1337 (1.1308 to 1.1366) | 1.1388 (1.136 to 1.1417) |
| Norway | 1.087 (1.0853 to 1.0887) | 1.0903 (1.0888 to 1.0918) | 1.0859 (1.0845 to 1.0874) |
| Sweden | 1.1014 (1.1002 to 1.1027) | 1.1079 (1.1068 to 1.1091) | 1.1087 (1.1076 to 1.1099) |
| **Southern Europe** |  |  |  |
| Albania | 1.1622 (1.1531 to 1.1714) | 1.1586 (1.1495 to 1.1679) | 1.1663 (1.1575 to 1.1752) |
| Bosnia and Herzegovina | 1.1475 (1.1425 to 1.1525) | 1.1552 (1.1502 to 1.1603) | 1.1585 (1.1538 to 1.1633) |
| Spain | 1.165 (1.1618 to 1.1682) | 1.1693 (1.166 to 1.1726) | 1.1705 (1.1671 to 1.1739) |
| Greece | 1.1824 (1.1786 to 1.1864) | 1.1835 (1.1799 to 1.1871) | 1.1882 (1.1848 to 1.1915) |
| Croatia | 1.1594 (1.1547 to 1.1641) | 1.1688 (1.1637 to 1.1739) | 1.1708 (1.1663 to 1.1755) |
| Italy | 1.1645 (1.161 to 1.168) | 1.1709 (1.1676 to 1.1742) | 1.1693 (1.1661 to 1.1725) |
| North Macedonia | 1.1524 (1.1448 to 1.1601) | 1.1494 (1.1411 to 1.1578) | 1.1593 (1.152 to 1.1666) |
| Malta | 1.1813 (1.1698 to 1.1929) | 1.1884 (1.1768 to 1.2002) | 1.1876 (1.176 to 1.1994) |
| Montenegro | 1.1478 (1.1357 to 1.1601) | 1.1498 (1.1371 to 1.1627) | 1.1529 (1.141 to 1.165) |
| Portugal | 1.1661 (1.1621 to 1.1701) | 1.1678 (1.1635 to 1.1721) | 1.1713 (1.1667 to 1.1759) |
| Serbia | 1.154 (1.1504 to 1.1576) | 1.1554 (1.1512 to 1.1596) | 1.1644 (1.1602 to 1.1686) |
| Slovenia | 1.1416 (1.1358 to 1.1475) | 1.1461 (1.14 to 1.1521) | 1.1528 (1.1473 to 1.1582) |
| **Western Europe** |  |  |  |
| Austria | 1.1268 (1.1226 to 1.1311) | 1.1356 (1.1315 to 1.1397) | 1.1376 (1.1335 to 1.1417) |
| Belgium | 1.1225 (1.1198 to 1.1251) | 1.1273 (1.1247 to 1.1298) | 1.1272 (1.1247 to 1.1298) |
| Switzerland | 1.1223 (1.1143 to 1.1304) | 1.1312 (1.1223 to 1.14) | 1.1279 (1.1193 to 1.1364) |
| Germany | 1.1252 (1.1241 to 1.1263) | 1.1297 (1.1285 to 1.131) | 1.1304 (1.1292 to 1.1317) |
| France | 1.141 (1.139 to 1.143) | 1.145 (1.143 to 1.147) | 1.1462 (1.1442 to 1.1481) |
| Luxembourg | 1.1268 (1.1181 to 1.1356) | 1.1307 (1.1209 to 1.1406) | 1.1303 (1.1198 to 1.1409) |
| Netherlands | 1.1382 (1.1284 to 1.148) | 1.1391 (1.1296 to 1.1486) | 1.1432 (1.1338 to 1.1528) |
| **Eastern Europe** |  |  |  |
| Bulgaria | 1.1597 (1.1544 to 1.165) | 1.1654 (1.1595 to 1.1712) | 1.1735 (1.1682 to 1.1788) |
| Belarus | 1.1479 (1.146 to 1.1499) | 1.1553 (1.1533 to 1.1572) | 1.1615 (1.1596 to 1.1634) |
| Czech Republic | 1.1259 (1.1231 to 1.1287) | 1.1329 (1.1304 to 1.1355) | 1.1349 (1.1321 to 1.1378) |
| Hungary | 1.1536 (1.1516 to 1.1555) | 1.1612 (1.1588 to 1.1636) | 1.1653 (1.1632 to 1.1674) |
| Moldova | 1.1734 (1.1694 to 1.1774) | 1.1762 (1.1722 to 1.1802) | 1.1821 (1.1781 to 1.186) |
| Poland | 1.1315 (1.1302 to 1.1328) | 1.1345 (1.1333 to 1.1357) | 1.1412 (1.1399 to 1.1426) |
| Romania | 1.159 (1.1557 to 1.1623) | 1.1626 (1.1585 to 1.1667) | 1.1709 (1.1676 to 1.1741) |
| Russian Federation | 1.1349 (1.1341 to 1.1356) | 1.1368 (1.136 to 1.1375) | 1.138 (1.1373 to 1.1388) |
| Slovak Republic | 1.1395 (1.1343 to 1.1446) | 1.1424 (1.1371 to 1.1478) | 1.1509 (1.146 to 1.1558) |
| Ukraine | 1.1684 (1.1667 to 1.1701) | 1.1715 (1.1699 to 1.173) | 1.1799 (1.1783 to 1.1815) |
| **Africa** |  |  |  |
| **Northern Africa** |  |  |  |
| Algeria | 1.1385 (1.135 to 1.142) | 1.1421 (1.1387 to 1.1455) | 1.1453 (1.1418 to 1.1488) |
| Egypt, Arab Rep. | 1.1177 (1.115 to 1.1203) | 1.1178 (1.1151 to 1.1205) | 1.1208 (1.1181 to 1.1235) |
| Libya | 1.1213 (1.1183 to 1.1242) | 1.1293 (1.1257 to 1.133) | 1.1262 (1.1228 to 1.1296) |
| Morocco | 1.0854 (1.0812 to 1.0896) | 1.0942 (1.09 to 1.0985) | 1.093 (1.0888 to 1.0973) |
| Sudan | 1.137 (1.1346 to 1.1394) | 1.1425 (1.1403 to 1.1448) | 1.1391 (1.1369 to 1.1413) |
| Tunisia | 1.114 (1.1086 to 1.1195) | 1.1209 (1.1158 to 1.126) | 1.12 (1.1143 to 1.1257) |
| **Sub-Saharan Africa** |  |  |  |
| Angola | 1.0542 (1.0524 to 1.0559) | 1.0555 (1.0534 to 1.0576) | 1.0586 (1.0567 to 1.0605) |
| Burundi | 1.0413 (1.0335 to 1.0491) | 1.0278 (1.0204 to 1.0353) | 1.0123 (1.005 to 1.0196) |
| Benin | 1.0957 (1.0908 to 1.1005) | 1.1048 (1.0996 to 1.11) | 1.1005 (1.0956 to 1.1054) |
| Burkina Faso | 1.1276 (1.1235 to 1.1317) | 1.1402 (1.1357 to 1.1447) | 1.135 (1.1309 to 1.1392) |
| Botswana | 1.0946 (1.0926 to 1.0967) | 1.0951 (1.0931 to 1.0972) | 1.1041 (1.102 to 1.1062) |
| Central African Republic | 1.0796 (1.0769 to 1.0822) | 1.0848 (1.0823 to 1.0873) | 1.0838 (1.0815 to 1.0861) |
| Cote d'Ivoire | 1.0804 (1.0773 to 1.0834) | 1.0852 (1.0822 to 1.0883) | 1.0834 (1.0803 to 1.0865) |
| Cameroon | 1.0672 (1.0626 to 1.0717) | 1.0718 (1.0672 to 1.0764) | 1.0725 (1.068 to 1.077) |
| Congo, Dem. Rep. | 1.0592 (1.058 to 1.0603) | 1.0538 (1.0526 to 1.0549) | 1.0543 (1.0531 to 1.0554) |
| Congo, Rep. | 1.0509 (1.0481 to 1.0537) | 1.0533 (1.0505 to 1.0562) | 1.0549 (1.0521 to 1.0577) |
| Djibouti | 1.1611 (1.1522 to 1.17) | 1.1595 (1.1507 to 1.1683) | 1.1576 (1.1483 to 1.1669) |
| Eritrea | 1.116 (1.108 to 1.124) | 1.1223 (1.1143 to 1.1303) | 1.1235 (1.1148 to 1.1322) |
| Ethiopia | 1.0697 (1.0659 to 1.0735) | 1.0705 (1.0667 to 1.0744) | 1.0721 (1.0683 to 1.0759) |
| Gabon | 1.0502 (1.0469 to 1.0534) | 1.0552 (1.0519 to 1.0585) | 1.0532 (1.0499 to 1.0565) |
| Ghana | 1.089 (1.0855 to 1.0925) | 1.0949 (1.0914 to 1.0985) | 1.0942 (1.0906 to 1.0977) |
| Guinea | 1.097 (1.0935 to 1.1005) | 1.1055 (1.1019 to 1.1091) | 1.1047 (1.1008 to 1.1085) |
| Gambia, The | 1.1177 (1.1073 to 1.1283) | 1.1235 (1.1121 to 1.1351) | 1.1354 (1.1224 to 1.1485) |
| Guinea-Bissau | 1.0986 (1.0912 to 1.1061) | 1.1104 (1.1027 to 1.1181) | 1.1225 (1.1146 to 1.1306) |
| Kenya | 1.0623 (1.0574 to 1.0672) | 1.0631 (1.0582 to 1.068) | 1.0668 (1.062 to 1.0716) |
| Liberia | 1.0755 (1.0706 to 1.0805) | 1.0753 (1.0704 to 1.0803) | 1.0743 (1.0694 to 1.0792) |
| Lesotho | 1.0151 (1.0089 to 1.0213) | 1.0177 (1.0107 to 1.0247) | 1.0223 (1.0142 to 1.0306) |
| Madagascar | 1.0579 (1.0556 to 1.0603) | 1.0553 (1.0524 to 1.0582) | 1.0605 (1.0569 to 1.0641) |
| Mali | 1.1552 (1.1522 to 1.1583) | 1.1623 (1.1591 to 1.1654) | 1.1636 (1.1605 to 1.1666) |
| Mozambique | 1.0668 (1.0647 to 1.0688) | 1.0697 (1.0677 to 1.0717) | 1.0676 (1.0655 to 1.0698) |
| Mauritania | 1.1664 (1.1616 to 1.1713) | 1.1741 (1.1696 to 1.1787) | 1.1813 (1.1757 to 1.187) |
| Mauritius | 1.0448 (1.0212 to 1.069) | 1.0408 (1.0169 to 1.0653) | 1.0487 (1.0246 to 1.0733) |
| Malawi | 1.0516 (1.0478 to 1.0553) | 1.0503 (1.0467 to 1.0539) | 1.0468 (1.0433 to 1.0504) |
| Namibia | 1.0704 (1.0677 to 1.0731) | 1.0807 (1.0781 to 1.0834) | 1.0864 (1.0835 to 1.0892) |
| Niger | 1.1597 (1.1574 to 1.162) | 1.1654 (1.163 to 1.1677) | 1.1592 (1.1568 to 1.1616) |
| Nigeria | 1.1112 (1.1081 to 1.1143) | 1.1132 (1.1101 to 1.1164) | 1.1102 (1.1072 to 1.1132) |
| Rwanda | 1.0365 (1.0289 to 1.0442) | 1.0156 (1.0082 to 1.0231) | 1.0098 (1.0024 to 1.0172) |
| Senegal | 1.1302 (1.1248 to 1.1357) | 1.1373 (1.132 to 1.1427) | 1.1473 (1.1409 to 1.1537) |
| Sierra Leone | 1.1011 (1.095 to 1.1072) | 1.1066 (1.1004 to 1.1128) | 1.1016 (1.0955 to 1.1077) |
| Somalia | 1.1101 (1.1079 to 1.1124) | 1.1135 (1.1112 to 1.1158) | 1.1116 (1.1094 to 1.1137) |
| Eswatini | 1.0561 (1.0445 to 1.0678) | 1.0587 (1.0467 to 1.0708) | 1.057 (1.0453 to 1.0689) |
| Chad | 1.142 (1.1395 to 1.1445) | 1.1445 (1.1422 to 1.1468) | 1.1423 (1.14 to 1.1447) |
| Togo | 1.0885 (1.0824 to 1.0947) | 1.0959 (1.0897 to 1.1022) | 1.0936 (1.0874 to 1.0999) |
| Tanzania | 1.04 (1.0383 to 1.0418) | 1.0411 (1.0393 to 1.0429) | 1.0411 (1.0392 to 1.0429) |
| Uganda | 1.044 (1.0406 to 1.0474) | 1.0346 (1.0312 to 1.0379) | 1.0393 (1.0359 to 1.0427) |
| South Africa | 1.0609 (1.0588 to 1.0631) | 1.068 (1.0657 to 1.0704) | 1.0704 (1.068 to 1.0728) |
| Zambia | 1.0588 (1.0571 to 1.0605) | 1.0531 (1.0514 to 1.0548) | 1.0535 (1.0517 to 1.0553) |
| Zimbabwe | 1.0736 (1.0703 to 1.077) | 1.0736 (1.0705 to 1.0767) | 1.073 (1.0698 to 1.0762) |
| **Asia** |  |  |  |
| **Central Asia** |  |  |  |
| Kazakhstan | 1.0541 (1.0526 to 1.0555) | 1.05 (1.0485 to 1.0516) | 1.0531 (1.0515 to 1.0547) |
| Kyrgyz Republic | 1.0149 (1.0102 to 1.0196) | 1.0175 (1.0127 to 1.0224) | 1.022 (1.0167 to 1.0273) |
| Tajikistan | 1.039 (1.0304 to 1.0475) | 1.0398 (1.031 to 1.0487) | 1.0407 (1.0323 to 1.0491) |
| Turkmenistan | 1.1032 (1.1009 to 1.1054) | 1.1049 (1.1027 to 1.107) | 1.1028 (1.1006 to 1.105) |
| Uzbekistan | 1.0866 (1.0826 to 1.0907) | 1.086 (1.0822 to 1.0898) | 1.0837 (1.0803 to 1.087) |
| **Southern Asia** |  |  |  |
| Afghanistan | 1.0747 (1.069 to 1.0804) | 1.0777 (1.0716 to 1.0839) | 1.0753 (1.0698 to 1.0808) |
| Bangladesh | 1.0588 (1.0563 to 1.0613) | 1.0583 (1.056 to 1.0607) | 1.0583 (1.0557 to 1.0609) |
| India | 1.0802 (1.0782 to 1.0822) | 1.0799 (1.0779 to 1.0819) | 1.0807 (1.0786 to 1.0827) |
| Iran, Islamic Rep. | 1.0981 (1.0949 to 1.1012) | 1.0987 (1.0958 to 1.1016) | 1.0923 (1.0896 to 1.0951) |
| Sri Lanka | 1.045 (1.0399 to 1.0501) | 1.0506 (1.0451 to 1.0561) | 1.0508 (1.0454 to 1.0562) |
| Nepal | 1.0536 (1.0447 to 1.0625) | 1.0538 (1.0451 to 1.0627) | 1.0569 (1.0473 to 1.0666) |
| Pakistan | 1.1044 (1.0998 to 1.109) | 1.1053 (1.1007 to 1.1099) | 1.1019 (1.0974 to 1.1064) |
| **Western Asia** |  |  |  |
| United Arab Emirates | 1.1459 (1.1409 to 1.1509) | 1.14 (1.1348 to 1.1452) | 1.1484 (1.143 to 1.1538) |
| Armenia | 1.0241 (1.0166 to 1.0316) | 1.0302 (1.0221 to 1.0383) | 1.0271 (1.0187 to 1.0356) |
| Azerbaijan | 1.0396 (1.0346 to 1.0445) | 1.0487 (1.0436 to 1.0539) | 1.0514 (1.0456 to 1.0571) |
| Cyprus | 1.0504 (1.0405 to 1.0604) | 1.0548 (1.0443 to 1.0654) | 1.055 (1.0456 to 1.0645) |
| Georgia | 1.0177 (1.012 to 1.0235) | 1.021 (1.0147 to 1.0273) | 1.0212 (1.0149 to 1.0275) |
| Iraq | 1.1432 (1.138 to 1.1485) | 1.1482 (1.1433 to 1.153) | 1.1413 (1.1368 to 1.1459) |
| Israel | 1.0607 (1.0545 to 1.067) | 1.0615 (1.0548 to 1.0682) | 1.064 (1.0554 to 1.0726) |
| Jordan | 1.0778 (1.0736 to 1.082) | 1.0793 (1.0751 to 1.0837) | 1.0843 (1.0797 to 1.0889) |
| Kuwait | 1.1586 (1.1493 to 1.1681) | 1.1599 (1.1498 to 1.1701) | 1.1881 (1.1776 to 1.1987) |
| Lebanon | 1.0355 (1.0272 to 1.0439) | 1.0357 (1.0281 to 1.0434) | 1.0419 (1.0353 to 1.0486) |
| Oman | 1.1171 (1.1095 to 1.1248) | 1.1172 (1.1095 to 1.1249) | 1.1204 (1.1127 to 1.1282) |
| West Bank and Gaza | 1.0532 (1.0472 to 1.0592) | 1.054 (1.0477 to 1.0603) | 1.0547 (1.0483 to 1.0611) |
| Saudi Arabia | 1.1214 (1.1193 to 1.1236) | 1.1259 (1.1237 to 1.1281) | 1.1303 (1.1282 to 1.1324) |
| Syrian Arab Republic | 1.0864 (1.0811 to 1.0917) | 1.0922 (1.0864 to 1.098) | 1.0925 (1.0871 to 1.0979) |
| Turkey | 1.0301 (1.0275 to 1.0328) | 1.0352 (1.0326 to 1.0378) | 1.0366 (1.034 to 1.0392) |
| Yemen, Rep. | 1.1014 (1.0971 to 1.1058) | 1.0963 (1.0921 to 1.1005) | 1.099 (1.0943 to 1.1037) |
| **Eastern Asia** |  |  |  |
| China | 1.0388 (1.0379 to 1.0398) | 1.0397 (1.0388 to 1.0406) | 1.0427 (1.0417 to 1.0437) |
| Japan | 1.0307 (1.0285 to 1.0329) | 1.0335 (1.0312 to 1.0358) | 1.0371 (1.035 to 1.0393) |
| Korea, Rep. | 1.0387 (1.0368 to 1.0406) | 1.0395 (1.0377 to 1.0414) | 1.0452 (1.0434 to 1.0471) |
| Mongolia | 1.0131 (1.0116 to 1.0147) | 1.0193 (1.0176 to 1.021) | 1.0162 (1.0146 to 1.0178) |
| Korea, Dem. People's Rep. | 1.0264 (1.0229 to 1.0299) | 1.0253 (1.0218 to 1.0288) | 1.0319 (1.028 to 1.0359) |
| **South-eastern Asia** |  |  |  |
| Indonesia | 1.0319 (1.0313 to 1.0325) | 1.0332 (1.0326 to 1.0339) | 1.0325 (1.0319 to 1.0331) |
| Cambodia | 1.0519 (1.0492 to 1.0546) | 1.0496 (1.047 to 1.0522) | 1.0528 (1.0498 to 1.0558) |
| Lao PDR | 1.0494 (1.0471 to 1.0516) | 1.0455 (1.0434 to 1.0477) | 1.0492 (1.047 to 1.0514) |
| Myanmar | 1.0551 (1.0528 to 1.0573) | 1.054 (1.0518 to 1.0561) | 1.0563 (1.054 to 1.0587) |
| Malaysia | 1.0283 (1.0267 to 1.0299) | 1.0285 (1.0269 to 1.0301) | 1.0311 (1.0295 to 1.0326) |
| Philippines | 1.0332 (1.0319 to 1.0345) | 1.0334 (1.0321 to 1.0347) | 1.0357 (1.0344 to 1.0369) |
| Singapore | 1.0401 (1.0227 to 1.0578) | 1.0417 (1.0229 to 1.0608) | 1.0419 (1.02 to 1.0642) |
| Thailand | 1.0543 (1.0524 to 1.0562) | 1.0525 (1.0508 to 1.0542) | 1.0609 (1.0588 to 1.063) |
| Vietnam | 1.0533 (1.0506 to 1.056) | 1.0491 (1.0467 to 1.0516) | 1.0506 (1.0483 to 1.0528) |
| **Oceania** |  |  |  |
| **Australia and New Zealand** |  |  |  |
| Australia | 1.075 (1.0713 to 1.0788) | 1.08 (1.0763 to 1.0838) | 1.0817 (1.078 to 1.0854) |
| New Zealand | 1.0114 (1.0056 to 1.0171) | 1.0116 (1.0059 to 1.0173) | 1.0113 (1.0056 to 1.017) |
| **Other regions in Oceania** |  |  |  |
| Fiji | 1.067 (1.053 to 1.0811) | 1.069 (1.0551 to 1.0832) | 1.0788 (1.0646 to 1.0931) |
| Papua New Guinea | 1.0966 (1.0916 to 1.1017) | 1.1058 (1.1007 to 1.111) | 1.0801 (1.0752 to 1.0851) |
