# Supplementary material for: Global, regional, and national burden of heatwave-related mortality from 1990 to 2019: A three-stage modelling study
Source: PLoS Med. 2024 May 14;21(5):e1004364. doi: 10.1371/journal.pmed.1004364 (PMC11093289; doi:10.1371/journal.pmed.1004364)
Supplement: S19 Table — (DOCX) [file pmed.1004364.s028.docx]

**S19 Table.** Results of sensitivity analyses on the effect size of heatwave event in comparison to non-heatwave days.

| Models | Coefficient | Standard error | P for difference |
| --- | --- | --- | --- |
| 1. Different mortality data (175 locations)^a^ |  |  |  |
| Using all-cause mortality | 0.063 | 0.007 | Reference |
| Using non-external cause mortality | 0.064 | 0.007 | 0.90 |
| 2. Different parameters (750 locations)^b^ |  |  |  |
| Primary model^c^ | 0.073 | 0.003 | Reference |
| Maximum lag days = 13 | 0.073 | 0.003 | 1.0 |
| Maximum lag days = 14 | 0.071 | 0.004 | 0.67 |
| Maximum lag days = 15 | 0.069 | 0.004 | 0.43 |
| Degree of freedom of lag days = 3 | 0.078 | 0.003 | 0.31 |
| Degree of freedom of lag days = 5 | 0.079 | 0.003 | 0.20 |
| Degree of freedom of seasonality = 3 | 0.079 | 0.003 | 0.22 |
| Degree of freedom of seasonality = 5 | 0.074 | 0.003 | 0.82 |
| Degree of freedom of seasonality = 6 | 0.075 | 0.004 | 0.68 |
| Degree of freedom of seasonality = 7 | 0.075 | 0.004 | 0.77 |
| 3. Adjustment for relative humidity (500 locations)^d^ |  |  |  |
| Without relative humidity | 0.064 | 0.004 | Reference |
| With relative humidity | 0.061 | 0.004 | 0.47 |

^a^ 175 locations in the MCC dataset both collected data on all-cause mortality and non-external cause mortality

^b^ Sensitivity analyses of changing parameters of modeling were performed based on the all 750 MCC locations.

^c^ The primary model was fitted using maximum lag 10 days, 4 degree of freedom of lag days, and 4 degree of freedom of seasonality.

^d^ 500 locations in the MCC dataset collected data on relative humidity.
