# Supplementary material for: Global, regional, and national burden of heatwave-related mortality from 1990 to 2019: A three-stage modelling study
Source: PLoS Med. 2024 May 14;21(5):e1004364. doi: 10.1371/journal.pmed.1004364 (PMC11093289; doi:10.1371/journal.pmed.1004364)
Supplement: S1 Fig — Average and the change per decade of excess deaths (A, B), death ratio (%, C, D), and deaths per 10 million residents (E, F) (based on the age structure of WHO standard population) associated with heatwaves per warm season during 1990–2019 at a spatial resolution of 0.5°×0.5°. (DOCX) [file pmed.1004364.s029.docx]

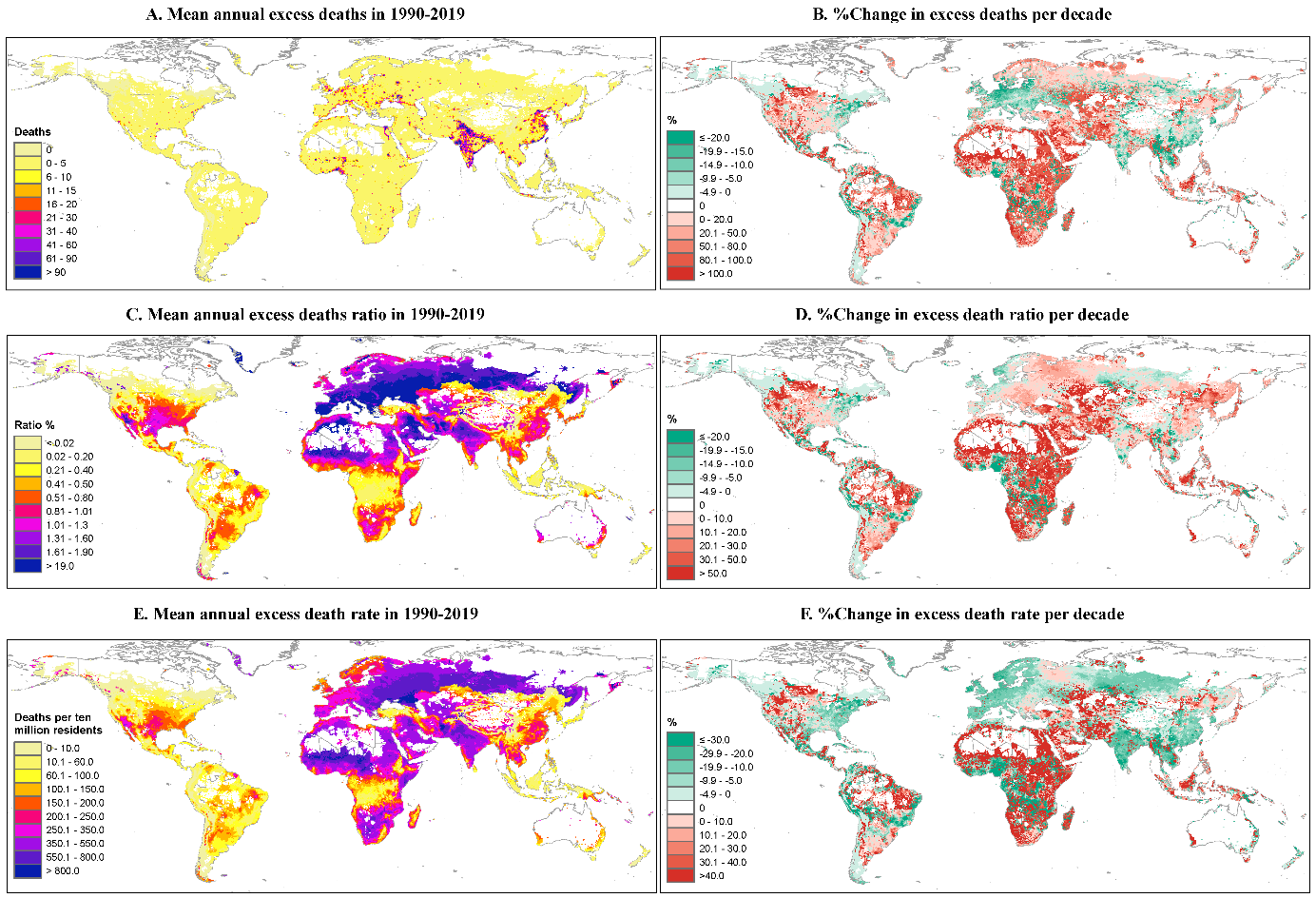


**S1 Fig.** Average and the change per decade of excess deaths (A-B), death ratio (%, C-D), and deaths per ten million residents (E-F) (based on the age structure of WHO standard population) associated with heatwaves per warm season during 1990-2019 at a spatial resolution of 0.5˚×0.5˚. Only grid cells with at least one annual death were included. $\%Change per decade=\frac{Change per decade}{The mean value in 1990-2019}\times100\%$. Change per decade is calculated using a linear regression. The base layer of the world map was imported from the public domain Natural Earth project (source: https://www.naturalearthdata.com/downloads/; terms of use: [www.naturalearthdata.com/about/terms-of-use/](http://www.naturalearthdata.com/about/terms-of-use/)). Summarized data at the regional level are provided in S7 Table, S9 Table, and S11 Table.
