# Supplementary material for: Global, regional, and national burden of heatwave-related mortality from 1990 to 2019: A three-stage modelling study
Source: PLoS Med. 2024 May 14;21(5):e1004364. doi: 10.1371/journal.pmed.1004364 (PMC11093289; doi:10.1371/journal.pmed.1004364)
Supplement: S3 Fig — (DOCX) [file pmed.1004364.s031.docx]

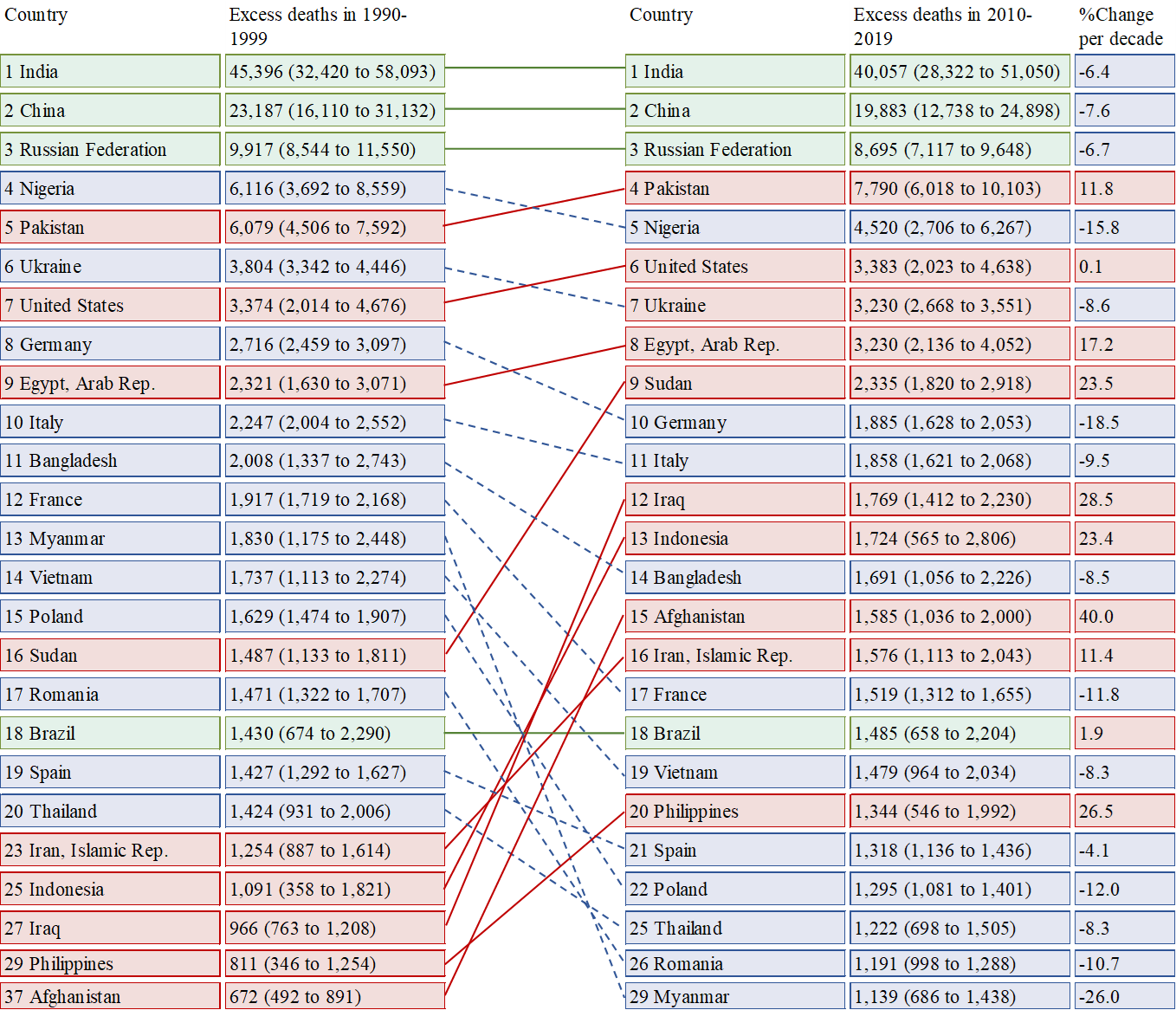


**S3 Fig.** Leading 20 countries for excess deaths (based on the age structure of WHO standard population) associated with heatwaves per warm season from 1990–1999 to 2010–2019. $\%Change per decade=\frac{Change per decade}{The mean value in 1990-2019}\times100\%$. Change per decade is calculated using a linear regression. Some countries might drop out or new countries may appear from the first to the last decade due to the changing burden. Underlying data are provided in S7 Table.
